# Mechanistic basis for protection against fatty liver disease by *CIDEB* loss-of-function mutations

**DOI:** 10.1016/j.jhep.2025.06.021

**Published:** 2025-07-04

**Authors:** Qiyu Zeng, Satish Patel, Xun Wang, Meng-Hsiung Hsieh, Zhijie Li, Xiongzhao Ren, Jingjing Wang, Dohun Kim, Shili Li, Xinping Gu, Greg Mannino, Gianna Maggiore, Xiangyi Fang, Lin Li, Min Zhu, Mengmeng Wang, Boyuan Li, Amaey Bellary, Koini Lim, Zhetuo Qi, Pushpa Pushpa, Mandour Omer Mandour, Vladimir Saudek, Tripti Sharma, Yu Zhang, Gerta Hoxhaj, Prashant Mishra, Purva Gopal, Peter Campbell, Matthew Hoare, David B. Savage, Hao Zhu

**Affiliations:** 1 Children’s Research Institute, Departments of Pediatrics and Internal Medicine, Center for Regenerative Science and Medicine, Children’s Research Institute Mouse Genome Engineering Core, University of Texas Southwestern Medical Center, Dallas, TX 75390, USA; 2 University of Cambridge Metabolic Research Laboratories, Wellcome Trust-MRC Institute of Metabolic Science, Cambridge, CB2 0QQ, UK; 3 Department of Pathology, University of Texas Southwestern Medical Center, Dallas, TX 75390, USA; 4 Cancer Genome Project, Wellcome Sanger Institute, Hinxton, Cambridgeshire CB10 1SA, UK; 5 University of Cambridge Department of Medicine, Cambridge Biomedical Campus, Cambridge, CB2 0QQ, UK and University of Cambridge Early Cancer Institute, Hutchison Research Centre, Cambridge Biomedical Campus, Cambridge, CB2 0XZ, UK

**Keywords:** somatic mutations, CIDEB, fatty liver disease, in vivo screening, β-oxidation

## Abstract

**Background & Aims:**

Somatic and germline *CIDEB* mutations are associated with protection from chronic liver diseases. The mechanistic basis and whether *CIDEB* suppression would be an effective therapy against fatty liver disease remain unclear.

**Methods:**

21 *CIDEB* somatic mutations were introduced into cells to assess functionality. In vivo screening was used to trace *Cideb* mutant clones in mice fed normal chow, western (WD), and choline-deficient, L-amino acid-defined, high-fat (CDA-HFD) diets. Constitutive and conditional *Cideb* knockout mice were generated to study *Cideb* in liver disease. Isotope tracing was used to evaluate fatty acid oxidation and de novo lipogenesis. Transcriptomics, lipidomics, and metabolic analyses were utilized to explore molecular mechanisms. Double knockout models (*Cideb/Atgl* and *Cideb/Ppara*) tested mechanisms underlying *Cideb* loss.

**Results:**

Most *CIDEB* mutations showed that they impair function, and lineage-tracing showed that loss-of-function clones were positively selected with CDA-HFD, but not all fatty liver inducing diets. *Cideb* KO mice were protected from WD, CDA-HFD, and alcohol diets, but had the greatest impact on CDA-HFD induced liver disease. Hepatocyte-specific *Cideb* deletion could ameliorate disease after MASLD establishment, modeling the impact of therapeutic siRNAs. *Cideb* loss protected livers via increased β-oxidation, specifically through ATGL and PPARa activation.

**Conclusions:**

*Cideb* deletion is more protective in some types of fatty liver disease. β-oxidation is an important component of the *Cideb* protective mechanism. *CIDEB* inhibition represents a promising approach, and somatic mutations in *CIDEB* might predict the patient populations that might benefit the most.

## Graphical abstract

**Graphical Abstract:**
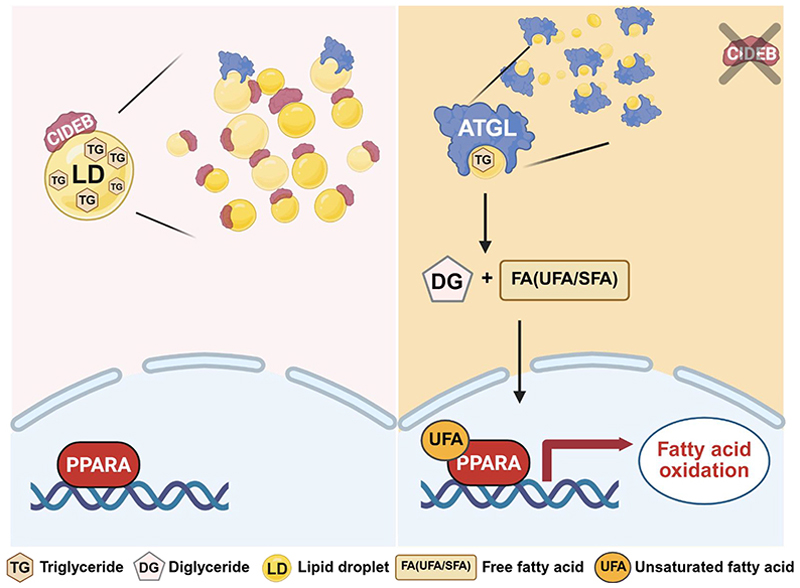

## Introduction

Recently, somatic genomic sequencing of liver disease samples from patients with alcohol related liver disease (ALD) and metabolic dysfunction-associated steatotic liver disease (MASLD) identified *Cell Death-Inducing DFF45-like Effector Protein B* (*CIDEB*) mutations that promote clonal fitness, likely through the reduction of hepatic lipid overload ^1^. In addition, human genetic studies showed that heterozygous germline mutations in *CIDEB* protect carriers from ALD and MASLD ^2^. Collectively, these studies suggest that *CIDEB* mutations are protective against lipotoxic liver disease. Consistently, whole-body *CIDEB* deletion in mice reduces body weight, improves insulin sensitivity and alleviates MASLD ^3^. In keeping with the reported action of all CIDE-family proteins (CIDEA, CIDEB, CIDEC), mechanistic studies show that CIDEB directly mediates lipid droplet (LD) growth ^4^. CIDEB has also been reported to affect hepatic lipid metabolism in other ways, including de novo lipogenesis (DNL) and VLDL particle lipidation ^5–7^.

The recent human genetic data have generated considerable interest in *CIDEB* as a therapeutic target for MASLD because of its growing prevalence and association with end-stage liver disease ^8^. Although whole-body *Cideb* deletion in mice appears to be beneficial, it is unclear if and how *CIDEB* somatic mutant clones are selected for within fatty livers, and if liver-specific loss of *CIDEB* would also protect against established MASLD. Furthermore, the molecular basis for CIDEB's effects on liver biology remain incompletely understood ^9^.

Here, we sought to understand the molecular basis for the selection of human *CIDEB* somatic mutations by exploring structure/function relationships. In addition to demonstrating that these mutants impair LD growth to at least some extent, these studies suggest that CIDEB oligomerization can result in the formation of a hydrophobic channel between adjacent LDs. We then asked if *Cideb* deficient clone selection was more pronounced in certain types of liver disease, and determined if this information might provide clues about the molecular basis for CIDEB's effects. We asked if the degree of positive clone selection is correlated with the therapeutic efficacy of liver-wide *Cideb* deletion. These studies showed that CIDEB predominantly facilitates unsaturated fatty acid accumulation in LDs, whereas its deficiency enhances fatty acid oxidation (FAO) in the liver. Finally, therapeutic modeling shows that liver-specific *CIDEB* suppression can ameliorate late stage manifestations of the disease such as fibrosis and liver cancer. These phenotypic and mechanistic findings pave the way for biomarker-based therapeutic studies using liver-specific targeting of *CIDEB*.

## Results

### Functional characterisation of somatic *CIDEB* mutations

**Fig. 1:**
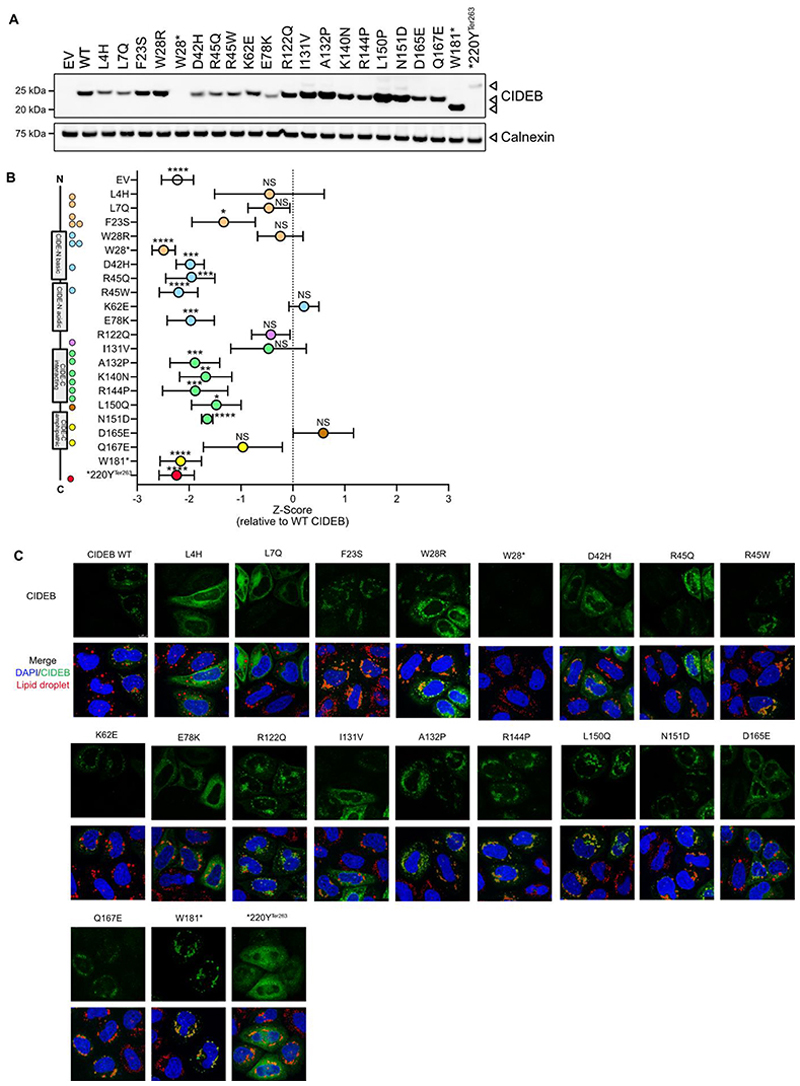
Impact of somatic *CIDEB* mutations on expression and LD enlargement. **(A)** Western blot analysis in oleate-loaded HeLa cells transfected with *CIDEB*. This blot is representative of 3 independent experiments. **(B)** Quantification of LD volume in oleate-loaded HeLa cells transfected with *CIDEB* mutants. Enlargement activity is represented as a Z-score where 0 is WT *CIDEB* **(C)** Representative images of LDs (red) and CIDEB (green) in oleate-loaded HeLa cells transfected with *CIDEB*. Nuclei were stained with DAPI (blue). Data are presented as mean ± SEM. Statistical analysis was performed using one-way ANOVA followed by Tukey's post hoc test. *p < 0.05, **p < 0.01, ***p < 0.001, ****p < 0.0001, **p < 0.01, N.S., not significant.

As all CIDE-family proteins facilitate LD growth via formation of contact sites between the surface phospholipid monolayers of adjacent LDs ^4,9–12^, we sought to measure the effects of *CIDEB* mutants on LD size *in vitro. CIDEB* missense mutations detected by somatic sequencing spanned all structurally validated and AlphaFold predicted domains (Fig. S1) ^1^. Among the mutations, two (W28* and W181*) were predicted to result in premature stop codons and likely nonsense mediated decay, whereas one distal frameshift mutant (Y220fs) was not predicted to undergo nonsense mediated RNA decay. When expressed in oleate loaded HeLa cells, expression of W28* and Y220fs were severely reduced whereas W181* was highly expressed (Fig. 1A). However, all three inhibited LD growth (Fig. 1B,C). All 18 tested missense variants were expressed at the protein level in HeLa cells, though some mutations, such as the E78K mutant, did reduce protein expression (Fig. 1A). While there was substantial variation in the impact on LD size, none significantly enhanced LD enlargement and 10 out of 18 missense variants significantly reduced LD size compared with wild-type (WT) *CIDEB* (Fig. 1B). LD targeting for most variants remained unperturbed, but 3 that did not reduce LD size (L4H, L7Q, K62E) showed more cytosolic distribution and reduced protein expression (Fig. 1A,C).

**Fig. 2:**
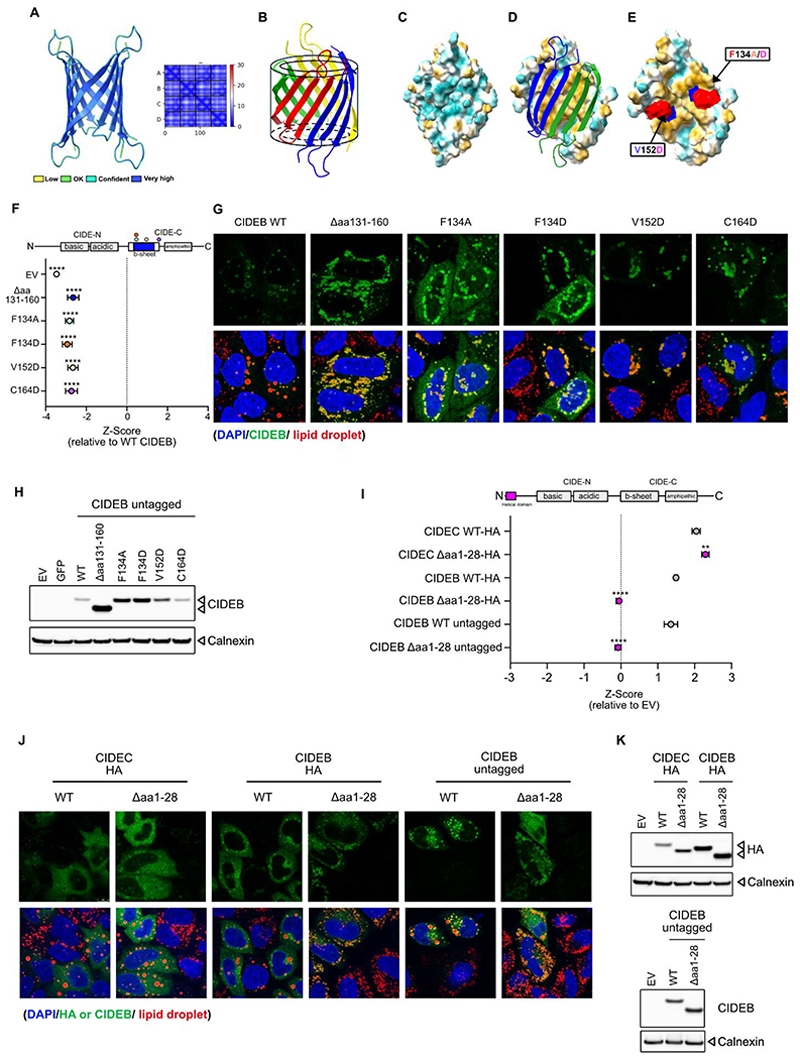
Disruption of the C-terminal β-sheet and N-terminus helix affects CIDEB-mediated LD enlargement. **(D)** AlphaFold prediction of the CIDEB β-sheet aa 127-170 tetramer (left panel). Predicted structure coloured according to predicted local distance difference (Quality parameters displayed below). Predicted aligned error (Å) between residues of the four protomers A to D, coloured according to scale on the left (right panel). Black lines separate the sequences of the monomeric units. **(E)** Model of CIDEB aa 127-170 forming a hollow β-barrel. The four protomers are rendered in different colors. **(F)** Surface representation of the external hydrophilic surface of the CIDEB putative channel. **(G)** Surface representation of the inner hydrophobic surface of the CIDEB putative channel. The two protomers are presented as ribbons to render the interior visible. **(H)** Surface representation of CIDEB putative channel. F145 (red) and V152 (blue) at the inner surface (two protomers shown). Lipid transfer is suppressed when mutated to D. The residues between the two V152 from two neighbouring protomers are C164 (green contour, Fig. S2). The Intensity of yellow and cyan corresponds to the degree of hydrophobicity and hydrophilicity, respectively. **(I)** Quantification of LD volume in oleate-loaded HeLa cells transfected with CIDEB β-sheet deletion and point mutants.Enlargement activity is represented as a Z-score where 0 is WT *CIDEB* **(J)** Representative images of LDs (red) and CIDEB (green) in oleate-loaded HeLa cells transfected with CIDEB β-sheet deletion and point mutants. Nuclei were stained with DAPI (blue). **(K)** Western blot analysis in oleate-loaded HeLa cells transfected with CIDEB β-sheet deletion and point mutants. **(L)** Quantification of LD volume in oleate-loaded HeLa cells transfected with wildtype (WT) or n-terminal helix deleted mutants (aa1-29) of HA-tagged CIDEC, HA-CIDEB or untagged CIDEB. LD enlargement activity is represented as a Z-score where 0 is empty vector (EV). **(M)** Representative images of LDs (red) and HA (green) or CIDEB (green) in oleate-loaded HeLa cells transfected with wildtype (WT) or n-terminal helix deleted mutants (aa1-29) of HA-tagged CIDEC, HA-CIDEB or untagged CIDEB. Nuclei were stained with DAPI (blue). **(N)** Western blot analysis in oleate-loaded HeLa cells transfected with HA tagged CIDEC, HA tagged CIDEB, or untagged CIDEB n-terminal helix deleted mutants. Data are presented as mean ± SEM. Statistical analysis was performed using one-way ANOVA followed by Tukey's post hoc test. **p < 0.01, ****p < 0.0001.

The structure of the globular CIDE-N domain (amino terminus of CIDE proteins) was previously resolved ^13^ and recent studies have characterized the amphipathic helical domain ^11^. Because the predicted β-sheet region of the CIDE-C domain (carboxy terminus) is less well studied, we next focused on this domain. Interestingly, AlphaFold predicted that it can fold into a tetrameric β-barrel with a central hydrophobic tunnel (Fig. 2A). This would lead to the formation of a channel (Fig. 2B) with a hydrophilic outer surface (Fig. 2C) and a hydrophobic pore (Fig. 2D), potentially enabling lipid transfer between LDs. To test this, we designed two ‘artificial’ missense mutants (F134D, V152D) to replace hydrophobic residues inside the putative channel with charged amino acids (Fig. 2E). Consistent with the predicted model, both mutations impaired LD growth (Fig. 2F-H). Parallel mutagenesis experiments with CIDEC showed similar results (Fig. S2A-C). The proposed channel is hydrophobic on the inside, which would allow the transfer of neutral lipids. Analysis of the evolution of all CIDE proteins showed that none of them have charged residues inside this putative channel (Fig. S2D). We further evaluated the stability of the inter-protomer β-barrel, which appears to be stabilized by π-π aromatic interactions inside the channel (Fig. S2E). In keeping with this observation, a missense F134A mutant that interrupts the aromatic interaction reduced LD growth (Fig. 2F-H). In addition, the model places C164 in two antiparallel protomers in an ideal position to form a stabilizing disulfide bond (Fig. 2E and Fig. S2F), and introducing a mutation C164D prevented CIDEB from enlarging LDs (Fig. 2F-H).

Four (K140N, R144P, L150Q, N151D) of the human somatic CIDEB variants in the β-sheet region substantially impaired LD growth whereas two (D165E and Q167E) did not (Fig. 1B). K140 and L150 are conserved in all CIDE proteins, whereas R144 is only conserved in CIDEA and CIDEB. Our tetrameric β-barrel model places K140 and R144 in the loop connecting the first and second β-strand of the putative channel, two at each entry. They could interact with the negative phospholipid surface of LDs. The mutations remove the positive charge and thus could abolish the interaction. L150 is a hydrophobic residue inside the channel. The mutation L150Q introduces a polar residue that could hinder the passage of lipids similarly to our designed mutations F134D and V152D. The tetrameric β-barrel model does not suggest an explanation for N151D introducing a positive charge at the external surface of the channel. N151 could be a glycosylation site and glycosylation is known to be important for CIDE protein function ^14^, although the specific sites have not yet been reported. The neutral mutations D165E (conserved) and Q167E (variable) at the external surface are predicted to bring about very mild changes.

Another region highlighted by the human genetic data was the extreme amino-terminus of CIDEB. In CIDEC, this region (amino acids 2-33) is predicted to fold into a short helix with no known function (Fig. S2G). However, deleting it entirely failed to perturb CIDEC action on LD growth. In CIDEB, both the somatic missense variants affecting this ‘domain’ (L4H and L7Q) very modestly reduced LD growth (Fig. 1B) but, in contrast to CIDEC, deletion of this region severely reduced CIDEB mediated LD growth (Fig. 2I-K). The predicted structure of this helix in CIDEB displays a continuous hydrophobic ridge on one side (L12, L13, V16, I19, F23, V27) with all charged residues on the other side (R14, R25, R26, E22) (Fig. S2H). The F23S mutation interrupted this bridge and significantly reduced LD size (Fig. 1B). The hydrophobicity of the residues in the ridge, though not the specific amino acid identity, is evolutionarily conserved (Fig. S2). The charged residues are conserved, with R14 replaced by K in some species. The hydrophobic ridge is also seen in CIDEA and CIDEC. While E22 and R26 conservation is unique to CIDEB, R14 in CIDEC and R25 in CIDEA are conserved. The pattern of conservation suggests a functional role for this helix, which could interact with other proteins. Collectively, these data suggest that the somatic CIDEB mutants are loss-of-function and that there is considerable variation in the extent of functional impairment. Specific mutations also deepen understanding of the fundamental mechanisms by which CIDE-family proteins facilitate LD growth.

### *Cideb* loss-of-function clones are positively selected in some fatty liver models

**Fig. 3:**
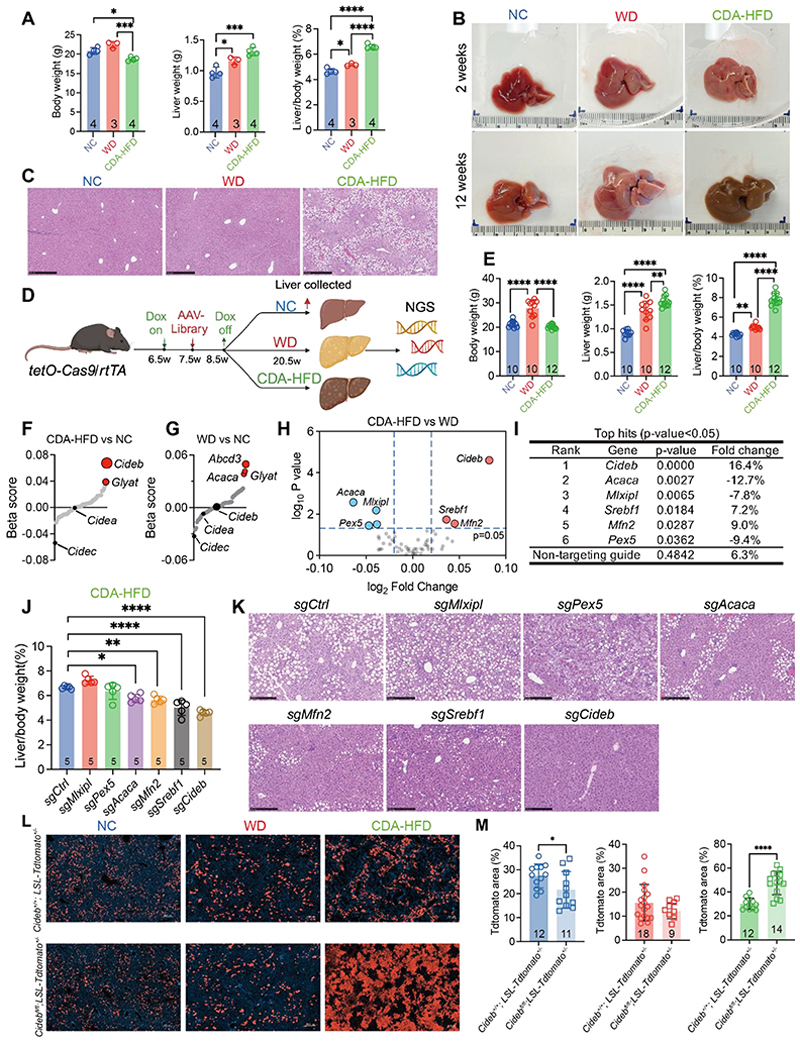
Positive selection of *Cideb* loss-of-function clones in two fatty liver models. **(A)** Body weight, liver weight, and liver/body weight ratio in WT mice fed with NC, WD, or CDA-HFD for 2 weeks. **(B)** Representative gross livers treated for 2 and 12 weeks on various diets. **(C)** Representative H&E staining of livers from Fig. 3A. **(D)** Schematic of the MOSAICS system. Cas9 was induced in iCas9 male mice using dox (1 mg/mL) from 6.5-8.5 weeks of age. AAV-sgRNA libraries targeting 55 genes were injected at 7.5 weeks of age. Mice were fed with NC, WD, or CDA-HFD for 3 months (n = 10, 10, and 12 mice). Livers were collected for genomic DNA and amplicon sequencing. **(E)** Body weight, liver weight, and liver/body weight ratio of screening mice from Fig. 3D. **(F)** β-scores from mice on CDA-HFD versus NC. Positive β-scores indicate sgRNA enrichment in the CDA-HFD group; negative scores indicate sgRNA non-enrichment relative to NC. **(G)** β-scores from mice on WD versus NC. **(H)** Volcano plot comparing WD and CDA-HFD, revealing enrichment of sgRNAs against *Acaca, Mlxipl*, and *Pex5* in WD, and *Cideb, Srebf1*, and *Mfn2* in CDA-HFD. **(I)** Genes associated with sgRNAs in **h** with significant enrichment (p < 0.05). **(J)** Cas9 was induced from 6.5-8.5 weeks of age. At 7.5 weeks, mice received a high dose of AAV-sgRNAs to delete genes in the liver. At 8.5 weeks, mice were fed CDA-HFD for 3 weeks. Liver/body weight ratios shown. **(K)** H&E staining of livers from Fig. 3J. **(L)** Low dose AAV8-TBG-Cre was used to induce mosaic Tomato labeling and *Cideb* deletion in hepatocytes. Mice were fed with NC, WD, or CDA-HFD for 3 months. Livers were collected 1 week after AAV injection and after 3 months of diets. Tomato images of liver sections are shown. **(M)** Tomato+ cell frequency in Fig. 3L. Data are presented as mean ± SEM. Statistical analysis was performed using one-way ANOVA followed by Tukey's post hoc test. *p < 0.05, **p < 0.01, ***p < 0.001, ****p < 0.0001.

In order to understand the conditions under which *CIDEB* loss-of-function clones are positively selected for in liver tissues, we used two MASLD diets: a western diet (WD), and a choline-deficient, L-amino acid-defined, high-fat diet (CDA-HFD) ^15^. Compared to mice fed with a WD for 2 weeks or 12 weeks, those fed with a CDA-HFD manifested higher liver/body weight ratios (Fig. 3A,B and Fig. S3A) and increased steatosis (Fig. 3C and Fig. S3B) despite lower body weights. *Cideb* mRNA levels in the liver remained relatively stable across the various diets (Fig. S3C,D), while *Cidea* and *Cidec* increased in both MASLD diets (Fig. S3E).

To compare *Cideb* deficient clones with others involved in metabolism, we employed the Method Of Somatic AAV-transposon *In vivo* Clonal Screening (MOSAICS) that can be used to track the fates of somatic mutations within the liver (Fig. 3D). MOSAICS can monitor the selection of mutant clones under different environmental conditions ^16^. We designed an AAV-sgRNA library targeting 55 genes including LD, DNL, FAO, and peroxisome genes (Fig. S4A and Table S1). After injecting an AAV-sgRNA library into dox-inducible Cas9 mice, we fed the mice with normal chow (NC), CDA-HFD, or WD for 12 weeks before sequencing liver samples and comparing the sgRNA abundance between the AAV library and the harvested livers (Fig. 3D). The liver and body weights were similar to those from mice that were not given AAV-sgRNA libraries (Fig. 3E and Fig. S3A).

Comparing CDA-HFD and NC revealed that clones with sgRNAs targeting *Cideb* and *Glyat* were most enriched in CDA-HFD livers (Fig. 3F). *Glyat*, or glycine N-acyltransferase, conjugates glycine with various acyl-CoA substrates to facilitate their excretion ^17^. In contrast, comparing WD and NC revealed that sgRNAs against *Acaca, Abcd3*, and *Glyat* were enriched whilst sgRNAs against *Cidea, Cideb, or Cidec* were not positively selected (Fig. 3G). ACACA (ACC1) converts acetyl-CoA to malonyl-CoA during fatty acid synthesis and ABCD3 transports molecules across peroxisomal membranes ^18^.

Comparing CDA-HFD vs. WD revealed that sgRNAs against *Cideb, Srebf1*, and *Mfn2* were specifically enriched in CDA-HFD (Fig. 3H,I). SREBP1c is the master transcriptional regulator of lipogenesis ^19^. MFN2 plays a role in mitochondrial fusion and regulates ER stress in the liver ^20^. Conversely, sgRNAs against genes such as *Acaca, Mlxipl* and *Pex5* were specifically enriched in WD (Fig. 3H,I and Fig. S4B). MLXIPL, or ChREBP, is a transcription factor that regulates fatty acid synthesis in response to changes in carbohydrate metabolism ^21^. PEX5 is a receptor for peroxisomal targeting sequences involved in protein import into peroxisomes ^22^. To determine the relationship between the degree of mutant clone selection and the extent of MASLD protection, we deleted *Cideb, Srebf1, Mfn2, Acaca, Mlxipl*, or *Pex5* in the entire liver using high dose AAV-sgRNAs, then treated with CDA-HFD. Interestingly, the degree of positive clone selection in CDA-HFD correlated with protection from CDA-HFD induced MASLD (Fig. 3J,K). Notably, sgRNAs that were not positively selected (*sgAcaca, sgMlxipl, sgPex5*) were associated with modestly beneficial or worse outcomes after CDA-HFD (Fig. 3J,K). We also used a lineage tracing approach to test this concept. By injecting a low dose of AAV-TBG-Cre, we deleted *Cideb* in a small fraction of hepatocytes, and simultaneously used Tomato as a reporter. After 3 months of NC, WD, or CDA-HFD feeding, we observed differences in Tomato clone expansion. In mice fed NC or WD, *Cideb*-deficient clone expansion was reduced compared to WT clones. In mice fed CDA-HFD, *Cideb*-deficient clone expansion was significantly increased compared to WT clones (Fig. 3L,M). This suggest that *Cideb* deletion confers a selective advantage specifically under the metabolic stress induced by the CDA-HFD, highlighting a diet-dependent clonal fitness effect. Thus, different diets can select for different mutations, and the degree of clonal selection correlates with the degree of disease protection.

### Liver-wide loss of *Cideb* is protective against multiple MASLD and ALD models

As *Cideb* KO clones were positively selected for in CDA-HFD but not in WD conditions, we asked if *Cideb* suppression might have a greater impact in CDA-HFD conditions. To these ends, we generated whole-body and conditional *Cideb* KO mice by using CRISPR-Cas9 germline gene editing. We used sgRNAs to target exon 2 because exons 1 and 2 of *Cideb* do not overlap with *Nop9*, a gene that overlaps in an antisense fashion with the 3’UTR of *Cideb* (Fig. S5A). Li’s group previously deleted exon 3-5 of *Cideb* ^3^, which removed part of the *Nop9* 3' UTR. While it is unknown if perturbing *Nop9* has any effects, our current KO model avoids this potential off-target effect. DNA and protein analysis confirmed the successful creation of whole-body *Cideb* KO mice missing exon 2 (Fig. S5B,C).

**Fig. 4:**
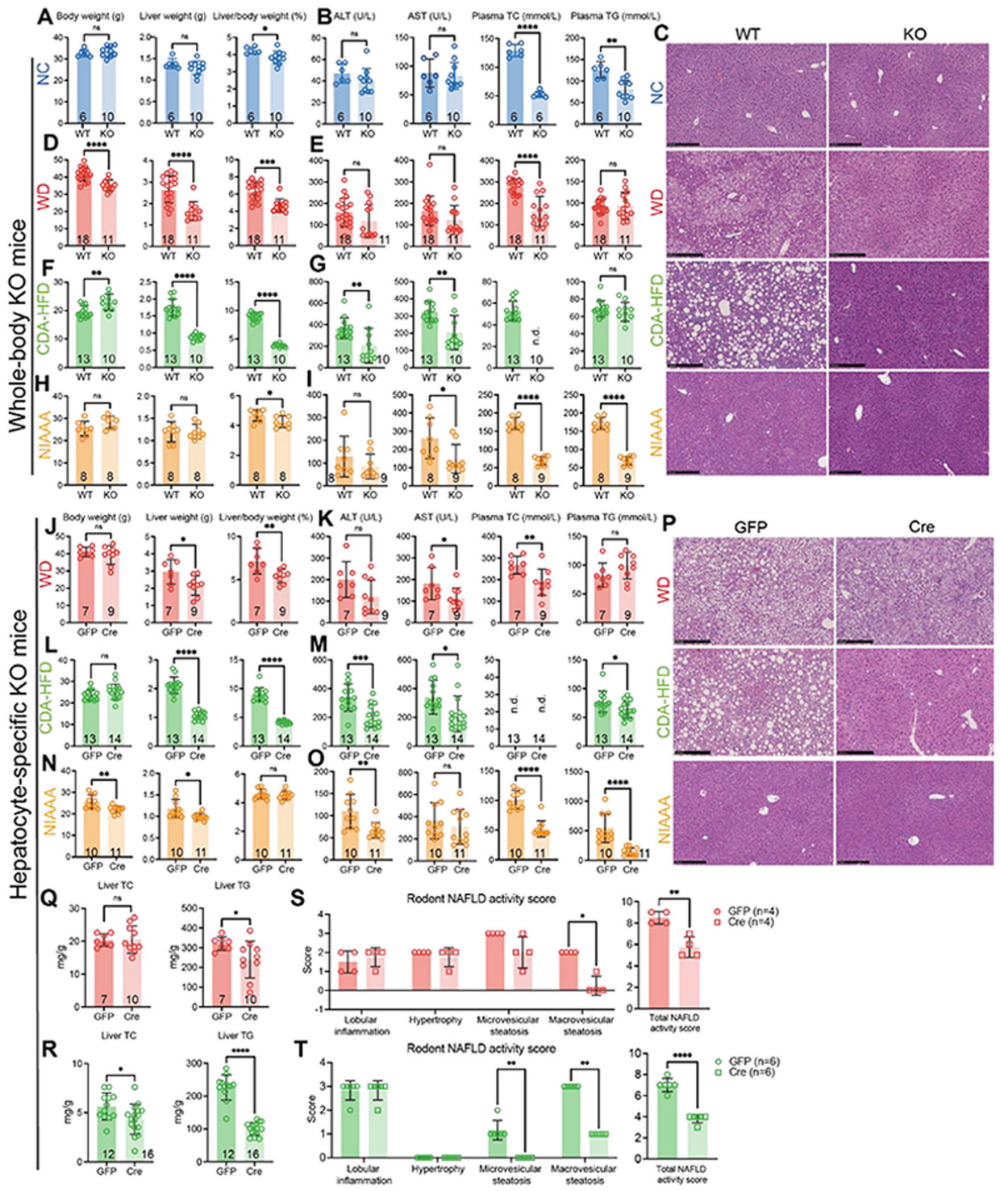
*Cideb* deletion in the liver protects against MASLD and ALD. **(A)** Body weight, liver weight, and liver/body weight ratio of WT and whole-body KO mice fed with NC for 12 weeks. **(B)** Plasma ALT, AST, cholesterol, and triglycerides of mice from Fig. 4A. **(C)** H&E of livers from mice treated with NC, WC, CDA-HFD, and NIAAA. **(D)** Weights of WT and whole-body KO mice fed with WD for 12 weeks. **(E)** Plasma measurements of mice from Fig. 4D. **(F)** Weights of WT and whole-body KO mice fed with CDA-HFD for 12 weeks. **(G)** Plasma measurements of mice from Fig. 4F. **(H)** Weights of WT and whole-body KO mice fed with the NIAAA diet for 4 weeks with weekly oral gavage of 5 g/kg ethanol. **(I)** Plasma measurements of mice from Fig. 4H. **(J)** Weights of *Cideb*^fl/fl^ mice injected with AAV-TBG-GFP or AAV-TBG-Cre, fed with WD for 24 weeks. **(K)** Plasma measurements of mice from Fig. 4J. **(L)** Weights of *Cideb*^fl/fl^ mice injected with AAV-TBG-GFP or AAV-TBG-Cre, fed with CDA-HFD for 12 weeks. **(M)** Plasma measurements of mice from Fig. 4L. **(N)** Weights of *Cideb*^fl/fl^ mice injected with AAV-TBG-GFP or AAV-TBG-Cre, fed with NIAAA diet for 4 weeks with weekly gavage of 5 g/kg ethanol. **(O)** Plasma measurements of mice from Fig. 4N. **(P)** H&E of livers from mice treated with WD, CDA-HFD, and NIAAA. **(Q)** Cholesterol and triglyceride content analysis from livers exposed to WD. **(R)** Cholesterol and triglyceride content analysis from livers exposed to CDA-HFD. **(S)** NAFLD activity score of the H&E sections from liver exposed to WD. The right panel represents the total NAFLD activity score, which is the sum of the four scores on the left (Each dot represents the average NAS score from 1–3 liver sections within a single slice). **(T)** NAFLD activity score of the H&E sections from liver exposed to CDA-HFD. The right panel represents the total NAFLD activity score, which is the sum of the four scores on the left (Each dot represents the average NAS score from 1–3 liver sections within a single slice). All mice in this figure were male, Data are shown as mean ± SEM. p-values were calculated by unpaired two-tailed Student’s t-test. *p < 0.05, **p < 0.01, ***p < 0.001, ****p < 0.0001.

Six week old male whole-body *Cideb* WT and KO mice were given NC, WD, or CDA-HFD for 12 weeks. On NC, there were no differences in body and liver weights between WT and KO mice, but liver/body weight ratios were reduced in KO mice (Fig. 4A). Alanine transaminase (ALT) and aspartate transferase (AST) were no different in WT and KO mice, but KO mice showed reduced plasma cholesterol and triglycerides (Fig. 4B). On NC, liver histology showed no differences in steatosis between WT and KO mice (Fig. 4C). On WD, KO mice had decreased body weight, liver weight, and liver/body weight ratios (Fig. 4D). ALT, AST and triglycerides were similar in WT and KO mice, but plasma cholesterol was reduced in the KO mice (Fig. 4E). On WD, liver histology showed a reduction in steatosis in KO mice (Fig. 4C). On CDA-HFD, KO mice had increased body weight, reduced liver weight, and reduced liver/body weight ratios (Fig. 4F). ALT, AST, and plasma cholesterol were decreased in KO mice, but triglycerides were unchanged (Fig. 4G). On CDA-HFD, liver histology showed a substantial reduction in steatosis in KO mice (Fig. 4C).

To determine if *Cideb* has an effect on ALD, we exposed mice to the NIAAA alcohol regimen for 4 weeks (Lieber-DeCarli liquid diet plus weekly oral gavage of ethanol) ^23^. KO mice had reduced liver/body weight ratios (Fig. 4H), as well as reduced plasma AST, cholesterol, and triglycerides (Fig. 4I). On NIAAA, liver histology showed a modest reduction in steatosis in KO mice (Fig. 4C). Overall, whole-body *Cideb* deletion protected mice from the effects of WD, CDA-HFD, and alcohol, but most effectively mitigated the effects of CDA-HFD (Fig. 4C).

To determine if organ-specific loss of *Cideb* mimics germline deletion, we generated *Cideb* floxed mice with loxP sequences flanking exon 2 (Fig. S5B). We used AAV-TBG-Cre to delete *Cideb* in hepatocytes at 6 weeks of age, resulting in CIDEB protein loss (Fig. S5D). *Cideb* WT and hepatocyte-specific KO mice were provided with a WD for 24 weeks or CDA-HFD for 12 weeks. After 24 weeks of WD feeding, mice with hepatocyte-specific deletion had decreased liver weight and liver/body weight ratios (Fig. 4J), but in contrast to the whole-body KO model, the hepatocyte-specific KO did not cause body weight reduction. Plasma AST and cholesterol levels were reduced in the hepatocyte-specific KO, while ALT trended down and triglyceride was unchanged (Fig. 4K). On CDA-HFD, hepatocyte-specific KO mice had similar body weights, but lower liver, and liver/body weight ratios (Fig. 4L). Plasma ALT, AST, and triglycerides were lower in the hepatocyte-specific KO mice and plasma cholesterol was below the limit of detection for both WT and KO mice (Fig. 4M). Hepatocyte-specific KO mice exposed to the NIAAA diet showed reduced body and liver weights, but the liver/body weight ratios did not change (Fig. 4N). Similar to the whole-body KO mice, plasma ALT, triglyceride, and cholesterol were reduced, but AST remained unchanged (Fig. 4O). In all MASLD and ALD models, hepatocyte-specific KO mice had reduced steatosis on histology; the magnitude of decrease was most pronounced with CDA-HFD (Fig. 4P). KO livers on both MASLD diets showed reduced liver triglycerides and KO livers on CDA-HFD showed reduced liver cholesterol (Fig. 4Q,R). We applied a rodent-adapted nonalcoholic fatty liver disease (NAFLD) activity scoring system to assess hepatic steatosis and liver injury. This analysis revealed that KO livers exhibited reduced macrovesicular steatosis, microvesicular steatosis, and hepatocyte hypertrophy under both CDA-HFD and WD feeding conditions (Fig. 4S,T).For both diets, female hepatocyte-specific KO mice showed similar but less pronounced phenotypes (Fig. S6). Altogether, both whole-body and hepatocyte-specific deletion of *Cideb* impaired MASLD and ALD. The hepatocyte-specific KO mice did not show weight loss, thus metabolic improvements were not simply a consequence of body weight change.

### After MASLD establishment, hepatocyte-specific *Cideb* deletion can reverse disease

**Fig. 5:**
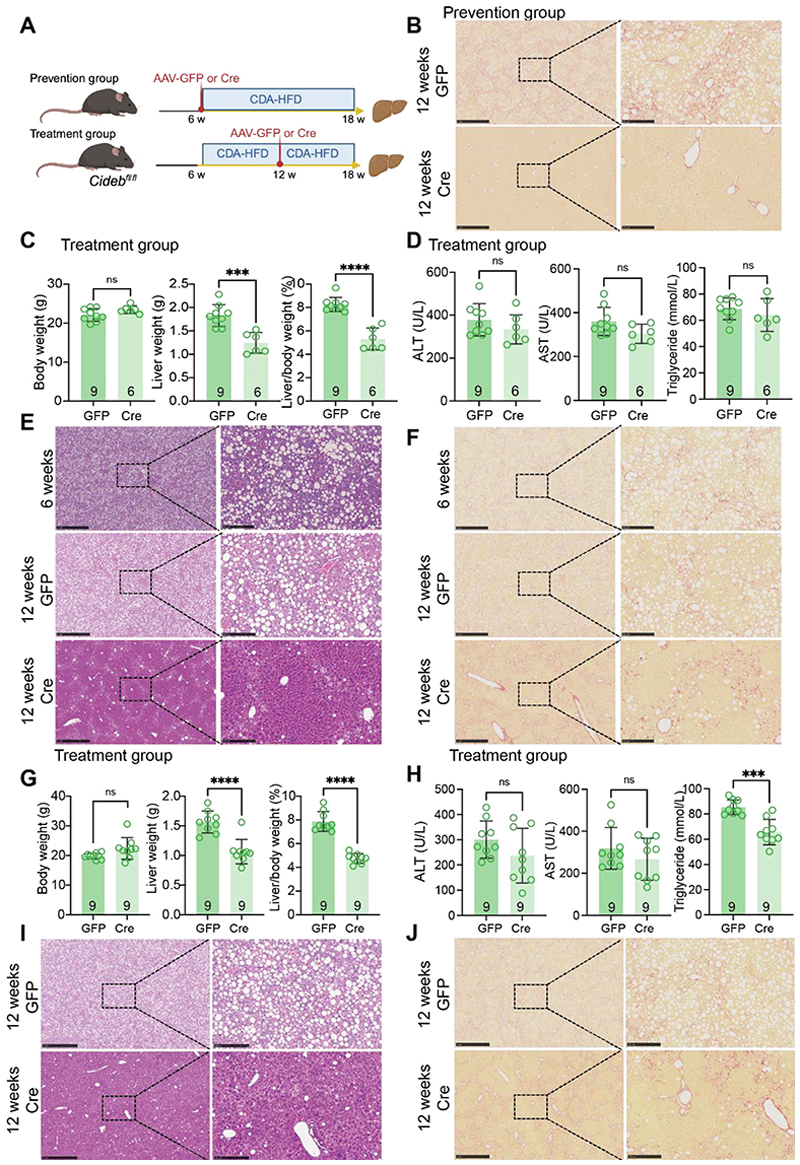
*Cideb* deletion reverses MASLD progression in male and female mice. **(A)** In the "Prevention" group, *Cideb*^*fl/fl*^ mice were injected with AAV-TBG-GFP or AAV-TBG-Cre at 6 weeks of age and fed CDA-HFD for 12 weeks (data shown in Fig. 4L). In the "Treatment" group, *Cideb*^*fl/fl*^ mice were fed CDA-HFD starting at 6 weeks of age for 6 weeks, then given AAV-TBG-GFP or AAV-TBG-Cre, then maintained on CDA-HFD for another 6 weeks before harvest. **(B)** Representative Sirius red staining of male “Prevention” group livers from Fig. 4L. **(C)** Body weight, liver weight, and liver/body weight ratio of male "Treatment" mice. **(D)** Plasma ALT, AST, and triglycerides from the male "Treatment" mice. **(E)** H&E staining of *Cideb*^*fl/fl*^ livers from the 6 week timepoint (when the AAV was given), and from the control and KO male "Treatment" mice at the 12 week timepoint. **(F)** Sirius red staining of livers from the male "Treatment" mice. **(G)** Weights of female "Treatment" mice. **(H)** Plasma measurements from the female "Treatment" mice. **(I)** H&E staining of livers from the female "Treatment" mice. **(J)** Sirius red staining of livers from the female "Treatment" mice. Data are shown as mean ± SEM. p-values were calculated by unpaired two-tailed Student’s t-test. ***p < 0.001, ****p < 0.0001.

Genetic experiments can mimic a potential hepatocyte targeting siRNA approach. We asked if hepatocyte-specific *Cideb* deletion after the establishment of MASLD could reverse some features of the disease. We used two cohorts. The first, referred to as the ‘Prevention group’, was discussed in the previous section. These male *Cideb*^*fl/fl*^ mice were given AAV-TBG-GFP (control) or AAV-TBG-Cre to induce *Cideb* deletion, then were given CDA-HFD for 12 weeks (Fig. 5A). The second cohort, referred to as the ‘Treatment group’, consisted of male *Cideb*^*fl/fl*^ mice that were fed CDA-HFD for 6 weeks, then given AAV-TBG-GFP or AAV-TBG-Cre, then resumed on CDA-HFD for an additional 6 weeks (Fig. 5A). In the ‘Prevention group’, MASLD characteristics were improved as discussed above (Fig. 4L,M,P) and fibrosis was decreased (Fig. 5B). In the ‘Treatment group’, KO mice had similar body weights, but reduced liver weights and liver/body weight ratios (Fig. 5C). Plasma ALT, AST, and triglyceride levels were similar in WT and KO mice (Fig. 5D). In the ‘Treatment’ experiment, control mice had similar levels of steatosis between 6 and 12 weeks, but KO mice had a robust reversal of steatosis after 6 weeks of CDA-HFD (Fig. 5E). That said, the modest amount of fibrosis observed was unchanged between WT and KO groups (Fig. 5F). Many of the same phenotypes were seen in female mice (‘Prevention’ in Fig. S6 and ‘Treatment’ in Fig. 5G-J). Hepatocyte-specific deletion was sufficient to reverse some, but not all MASH features.

### Unsaturated fats preferentially accumulate in LDs and *Cideb* deletion mitigates this

The impact of *Cideb* deletion in the CDA-HFD setting was increased in comparison to the WD setting. Lipidomics revealed differences in the types of free fatty acids (FFAs) present in the diets. The proportion of unsaturated fatty acids was higher in CDA-HFD than in WD (Fig. S7A). To understand if differences in the diets were also seen within the livers of mice, we performed FFA analysis on livers exposed to WD or CDA-HFD. CDA-HFD fed livers contained a higher proportion of unsaturated vs. saturated fatty acids compared to WD fed livers (Fig. S7B).

To determine if unsaturated fats accumulate in LDs more than saturated fats ^24,25^, we provided different saturated and unsaturated fatty acids (100 µM, 16h) to human (HepG2, Huh7) and mouse (H2.35) liver cancer cell lines. Saturated fatty acids included palmitic (16:0), stearic (18:0), and arachidic acid (20:0). The unsaturated fatty acids included oleic (18:1), linolenic (18:3), stearidonic (18:4), mead (20:3), arachidonic (20:4), and eicosapentaenoic acid (20:5). Compared to saturated fatty acids, the unsaturated fatty acids resulted in the formation of larger LDs (Fig. S7C,D). We repeated these experiments using primary C57BL/6 hepatocytes and the results were similar (Fig. S7E,F), supporting the idea that unsaturated fatty acids form LDs more efficiently than saturated fatty acids in the liver.

To determine if *Cideb* KO mice have altered accumulation of fatty acid species, we performed FFA analysis on WT and *Cideb* KO livers on CDA-HFD. There was little change in the relative levels of saturated fatty acids between WT and KO, but unsaturated fatty acids were disproportionately decreased in KO livers (Fig. S8A). To corroborate these findings, we also incubated primary hepatocytes from control *Cideb*^*fl/fl*^ and liver-specific *Alb-Cre; Cideb*^*fl/fl*^ mice in media containing predominantly saturated or unsaturated fatty acids. While unsaturated fatty acids resulted in larger LDs in WT hepatocytes, saturated fatty acids did not have a similar effect on LD formation (Fig. S7C,F). However, in KO hepatocytes, unsaturated fatty acids were unable to form LDs (Fig. S8B,C). Overall, LDs were smaller in KO hepatocytes, regardless of exogenous fatty acid source. This suggests that deleting *Cideb* impairs the enlargement of LDs mainly through the reduction of unsaturated fatty acids.

### Loss of *Cideb* did not reduce DNL or increase VLDL secretion

While CDA-HFD results in more unsaturated fatty acid accumulation within LDs, and *Cideb* deletion primarily reduces accumulation of unsaturated fatty acids, it is still unclear why there is a net reduction of neutral lipids in KO livers. We sought to determine if *Cideb* deficiency alters lipid delivery/uptake, synthesis, secretion, or oxidation. MASLD is most commonly associated with obesity. As indicated previously, whole-body *Cideb* deletion resulted in an increase in body weight in mice fed the CDA-HFD (Fig. 4F), so body weight cannot explain the reduced steatosis seen in the *Cideb* deficient mice. The increased *Cideb* KO body weight could be caused by reduced *Gdf15* and/or *Fgf21* secondary to improved liver health (Fig. 8D).

**Fig. 6:**
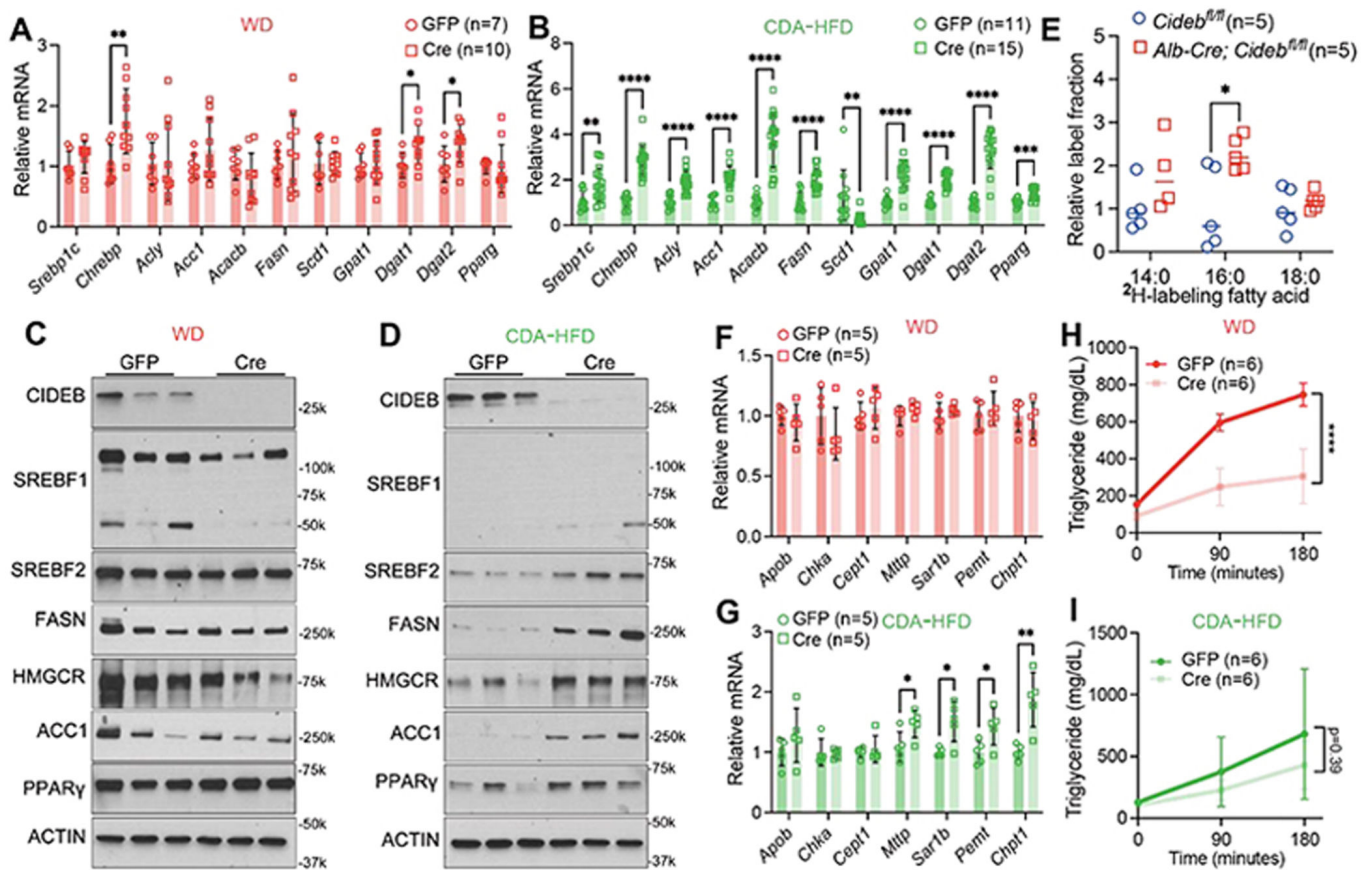
Influence of *Cideb* KO on DNL and VLDL lipidation/secretion. **(A)** qPCR of DNL genes from livers fed with 24 weeks of WD. **(B)** qPCR of DNL genes from livers fed with 12 weeks of CDA-HFD. **(C)** Western blot of DNL-related proteins in livers exposed to WD for 24 weeks. **(D)** Western blot of DNL-related proteins in livers exposed to CDA-HFD for 12 weeks. **(E)** Total labeled fraction of liver fatty acid species after 5 hours of short-term ^2^H_2_O tracing in *Cideb*^*fl/fl*^ and *Alb-Cre; Cideb*^*fl/fl*^ mice. **(F)** VLDL-related mRNA expression from livers treated with 24 weeks of WD. Data taken from RNA-seq analysis. **(G)** VLDL-related mRNA expression from livers treated with 12 weeks of CDA-HFD. Data taken from RNA-seq analysis. **(H)** Poloxamer-407 assay on mice fed with WD for 1 week and fasted overnight. Plasma triglyceride levels were measured. **(I)** Poloxamer-407 assay on mice fed with CDA-HFD for 1 week and fasted overnight. Data are shown as mean ± SEM. p-values were calculated by unpaired two-tailed Student’s t-test. *p < 0.05, **p < 0.01, ***p < 0.001, ****p < 0.0001.

We then assessed DNL pathways to determine if decreased lipogenesis could account for improved MASLD ^26^. KO livers on WD showed increases in *Chrebp, Dgat1* and *Dgat2*, but no changes in the other DNL mRNAs (Fig. 6A). KO livers on CDA-HFD showed an increase in DNL pathway mRNAs including *Srebp1c, Chrebp, Acly, Acc1, Acacb, Fasn, Gpat1, Dgat1, Dgat2* and *Pparγ* (Fig. 6B). In addition, SREBF1 and HMGCR were lower in KO livers on WD (Fig. 6C), but SREBF1, SREBF2, FASN, HMGCR, ACC1 and PPARγ protein expression were higher in KO livers on CDA-HFD (Fig. 6C). This suggests that on CDA-HFD, DNL is likely to be increased rather than decreased in KO livers, whereas on WD, DNL was only modestly lower in KO livers.

To functionally assess DNL in the CDA-HFD setting, we performed isotope enrichment analysis with a single dose of deuterated water (^2^H_2_O) followed by assessment of liver samples 5 hours later. In this assay, ^2^H_2_O effectively labels the hydrogen atoms in fatty acids via transfer from NADPH during fatty acid synthesis ^27,28^ . Therefore, ^2^H_2_O is used to measure DNL by quantifying the relative ^2^H enrichment of lipids ^27,28^. While labeled fatty acids between WT and KO livers fed CDA-HFD were similar, palmitic acid was increased in the KO suggesting modestly increased DNL (Fig. 6E). Thus, *Cideb* deficient mice were not protected from MASLD through decreases in DNL.

Increased disposal of lipids through very low density lipoprotein (VLDL) lipidation/secretion could represent an alternative mechanism for reduced MASLD. However, *Cideb* deletion is predicted to impair VLDL lipidation ^29^ and CDA-HFD induces steatosis at least in part by impairing VLDL secretion as is reflected in the low plasma triglycerides seen in this context. Nevertheless, we considered this pathway in our models. In the RNA-seq data from the WD model, the expression of key VLDL-related mRNAs was similar in WT and KO livers (Fig. 6F). In the RNA-seq data from the CDA-HFD model, *Apob, Chka*, and *Cept1* were similar in WT and KO livers, while *Mttp, Sar1b, Pemt*, and *Chpt1* were modestly increased in KO livers (Fig. 6G). Next, we functionally tested VLDL secretion. Poloxamer 407 inhibits Lipoprotein lipase, thereby reducing hydrolysis of triglycerides and facilitating VLDL accumulation. In this assay, we observed reduced VLDL secretion in KO mice on both WD and CDA-HFD (Fig. 6H,I), which is consistent with *Cideb* KO mice on WD having reduced plasma cholesterol (Fig. 4E,K), an observation that is also favorable for therapeutic approaches. Overall, these data suggest that *Cideb* deletion does not protect from MASLD through increased lipid export from the liver.

### *Cideb* deletion in the liver in mice increases hepatic FAO

**Fig. 7:**
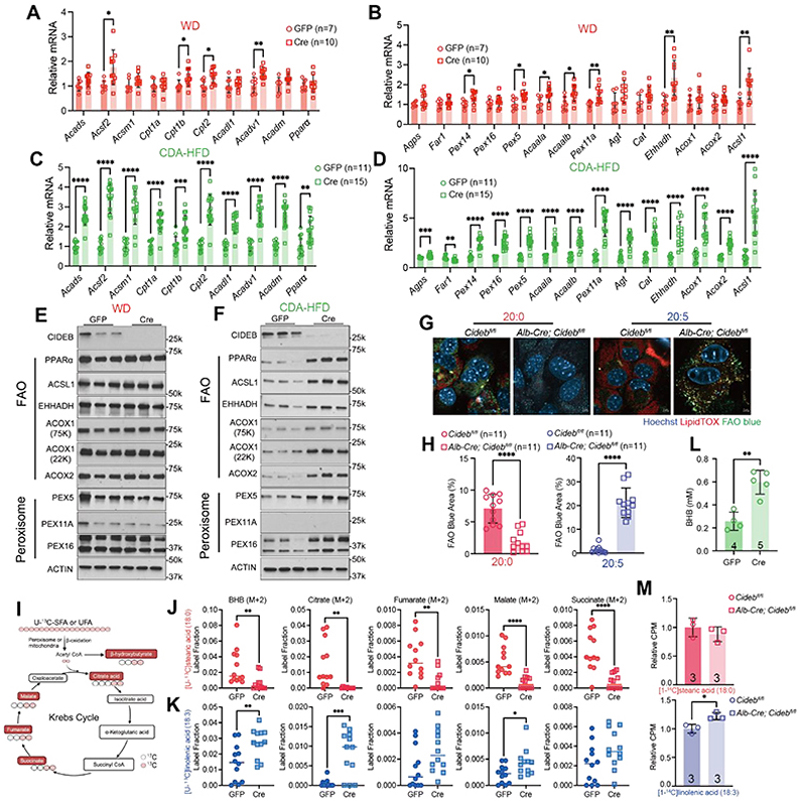
*Cideb* deletion increases FAO. **(A)** qPCR of FAO genes from livers fed with 24 weeks of WD. **(B)** qPCR of peroxisome-related genes from livers fed with 24 weeks of WD. **(C)** qPCR of FAO genes from livers fed with 12 weeks of CDA-HFD. **(D)** qPCR of peroxisome-related genes from livers fed with 12 weeks of CDA-HFD. **(E)** Western blot analysis of FAO and peroxisome-related proteins in livers exposed to WD for 24 weeks. The CIDEB and ACTIN blots are the same as shown in Fig. 6C. **(F)** Western blot analysis of FAO- and peroxisome-related proteins in livers exposed to CDA-HFD for 12 weeks. The CIDEB and ACTIN blots are the same as shown in Fig. 6D. **(G)** Representative FAO blue (green) of primary hepatocytes treated with arachidic acid (20:0) or arachidonic acid (20:5) overnight from *Cideb*^fl/fl^ and *Alb-Cre; Cideb*^*fl/fl*^ mice. LDs were stained with LipidTOX (red) and nuclei were stained with Hoechst (blue) (scale bars = 5 µm). **(H)** Quantification of LD area from Fig. 7G. **(I)** Schematic of [U-^13^C]linolenic and [U-^13^C]stearic acid tracing. **(J)** Fractional enrichment of indicated liver isotopologues in *Cideb*^*fl/fl*^ and *Alb-Cre; Cideb*^*fl/fl*^ mice after 2-hour infusion with [U-^13^C]stearic acid (n=12, 12 mice). **(K)** Fractional enrichment of indicated liver isotopologues in *Cideb*^fl/fl^ and *Alb-Cre*; *Cideb*^fl/fl^ mice after 2-hour infusion with [U-^13^C]linolenic acid (n=12, 12 mice). **(L)** Plasma BHB from *Cideb*^*fl/fl*^ mice injected with AAV-TBG-GFP or AAV-TBG-Cre fed with CDA-HFD for 1 month. **(M)** Relative FAO activity was assessed by measuring counts per minute (CPM) of ^14^CO_2_ production in primary hepatocytes isolated from *Cideb*^fl/fl^ and *Alb-Cre*; *Cideb*^fl/fl^ mice. The hepatocytes were treated with [1-^14^C]stearic acid or [1-^14^C]linolenic acid to evaluate the effect of *Cideb* deletion on fatty acid metabolism. Data are shown as mean ± SEM. p-values were calculated by unpaired two-tailed Student’s t-test. *p < 0.05, **p < 0.01, ***p < 0.001, ****p < 0.0001.

The CDA-HFD RNA-seq Gene Set Enrichment Analysis (GSEA) revealed upregulation of pathways related to bile acid metabolism, fatty acid metabolism, xenobiotic metabolism, adipogenesis, oxidative phosphorylation, and peroxisomes in KO vs. WT livers (Fig. S9A,B). On both diets, mRNAs related to FAO (*Acads, Acsf2, Acsm1, Cpt1a, Cpt1b, Cpt2, Acadl1, Acadv1, Acadm, Ppara*) and peroxisomes (*Agps, Pex14, Pex16, Pex5, Acaala, Acaalb, Pex11a, Agt, Cat, Ehhadh, Acox1, Acox2, Acsl1*) were upregulated (Fig. 7A-D). Proteins involved in FAO and peroxisome pathways were also increased in KO livers, but more in the CDA-HFD than in the WD setting (Fig. 7E,F).

To quantify FAO in primary hepatocytes with and without CIDEB, we used a reagent that visualizes FAO with green fluorescence. In cells exposed to the saturated arachidic acid (20:0), FAO blue signal was reduced in *Cideb* KO vs. WT hepatocytes, while in cells exposed to the unsaturated arachidonic acid (20:4), FAO blue signal was increased in KO vs. WT hepatocytes (Fig. 7G,H). To evaluate in vivo changes in FAO, *Cideb*^fl/fl^ and *Alb-Cre*; *Cideb*^fl/fl^ mice were infused with unsaturated ([U-^13^C]linolenic acid (18:3)) or saturated fatty acids ([U-^13^C]stearic acid (18:0)) for 2 hours (Fig. 7I). In KO mice, injection with saturated fatty acids led to a significant decrease in ^13^C-labeled β-hydroxybutyrate (BHB), citrate, fumarate, malate, and succinate, whereas injection with unsaturated fatty acids resulted in an increase in these metabolites, indicating increased FAO (Fig. 7J,K). To assess the overall production of ketones through FAO, we measured BHB in the plasma of CDA-HFD treated mice, showing that liver-specific KO mice had higher plasma BHB levels (Fig. 7L). Consistently, similar findings were made in primary hepatocytes using a gold standard radioactive assay that directly measures CO_2_ release from fatty acids. In KO hepatocytes, this showed increased β-oxidation when linoleic acid is provided, but no significant difference with stearic acid (Fig. 7M). Collectively, these results suggest a relative increase in β-oxidation of unsaturated fatty acids, and an overall increase in FAO in *Cideb* KO mice.

### PPARα and ATGL are effectors of increased FAO in *Cideb* KO livers

**Fig. 8:**
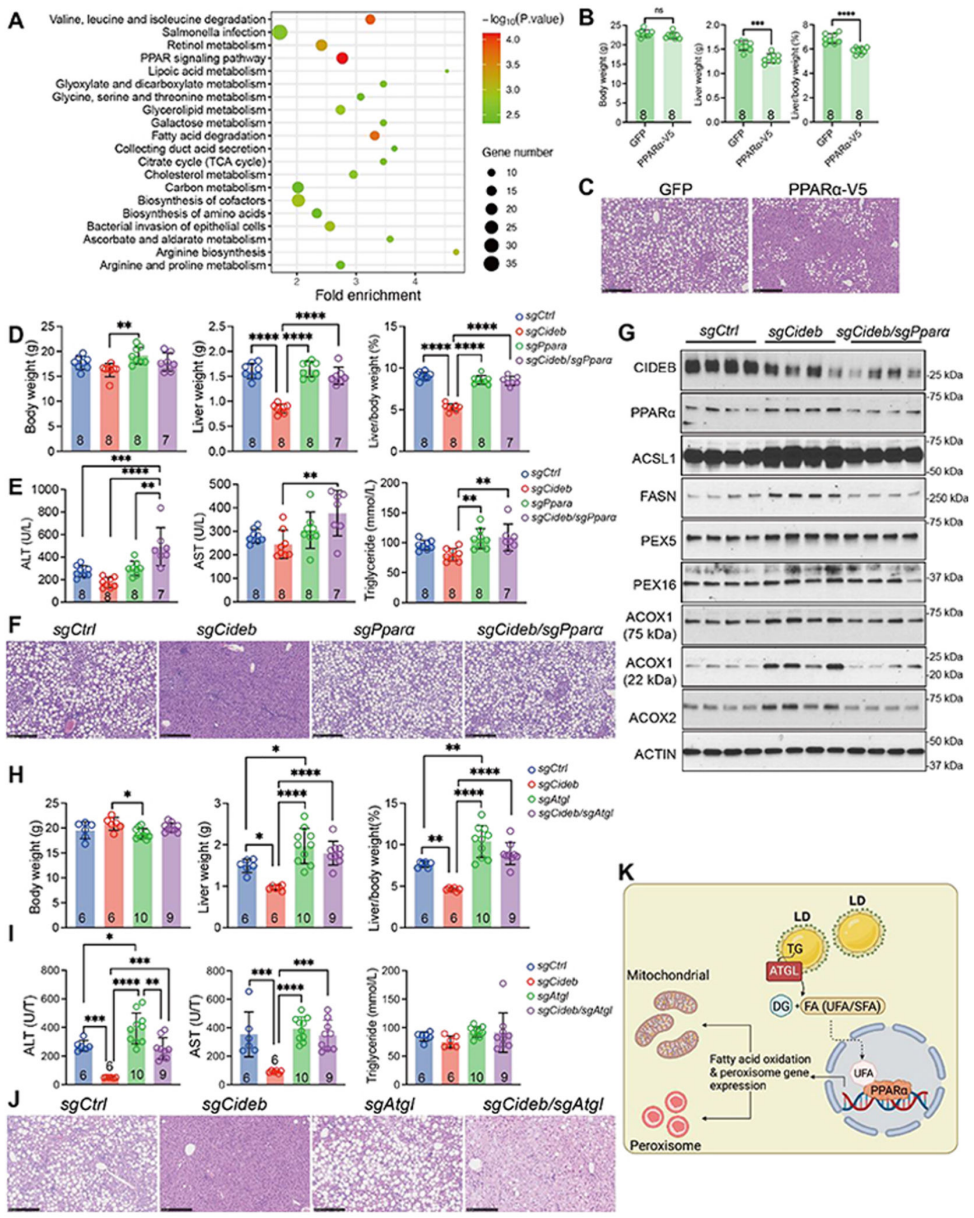
*Pparα* and *Atgl* deletion rescue the protective effects of *Cideb* deletion **(A)** Bubble plot showing differentially expressed pathways between WT and KO livers treated with 12 weeks of CDA-HFD. **(B)** Body weight, liver weight, and liver/body weight ratio of WT mice injected with AAV-TBG-GFP or AAV-TBG-PPARα-V5 and treated with CDA-HFD for 3 weeks. **(C)** Representative H&E staining of livers from Fig. 8B. **(D)** Body weight, liver weight, and liver/body weight ratio in male iCas9 mice given high-dose AAV-sgRNAs (*sgCtrl, sgCideb, sgPparα*, or *sgCideb*/*sgPparα*). At 8.5 weeks, mice were fed CDA-HFD for 3 weeks. **(E)** Liver function tests from mice in Fig. 8D. **(F)** Representative H&E staining of livers from Fig. 8D. **(G)** Western blot analysis of livers from Fig. 8D. **(H)** Body weight, liver weight, and liver/body weight ratio in male iCas9 mice given high-dose AAV-sgRNAs (*sgCtrl, sgCideb, sgAtgl*, or *sgCideb*/*sgAtgl*). At 8.5 weeks, mice were fed CDA-HFD for 3 weeks. **(I)** Liver function tests from mice in Fig. 8H. **(J)** Representative H&E staining of livers from Fig. 8H. **(K)** Model for how ATGL affects LDs, which subsequently influences PPARα function. Data are presented as mean ± SEM. Statistical analysis was performed using one-way ANOVA followed by Tukey’s post hoc test. *p < 0.05, **p < 0.01, ***p < 0.001, ****p < 0.0001.

KEGG analysis indicated that PPAR signaling was upregulated on the CDA-HFD (Fig. 8A). Specifically, peroxisome proliferator-activated receptor alpha (PPARα) was upregulated on mRNA and protein levels (Fig. 7C,F). Given that PPARα is a nuclear receptor and transcription factor that regulates lipid catabolism and has been implicated in mediating increased FAO in the context of altered LD fatty acid regulation ^30–32^, we hypothesized that upregulation of PPARα might be a key determinant of the increased β-oxidation seen in *Cideb* KO livers. We first asked if PPARα overexpression alone is enough to reduce fatty liver disease. AAV was used to overexpress GFP or PPARα-V5 in WT mice, then mice were fed CDA-HFD. Three weeks later, livers with PPARα overexpression showed reduced steatosis (Fig. 8B,C).

While overexpression of PPARα alone is sufficient to inhibit MASLD, it is unclear if PPARα is required for the protective phenotype caused by *Cideb* loss. We used iCas9 mice and AAV-sgRNA vectors to generate control, *Cideb* single, *Pparα* single, or *Cideb*; *Pparα* double KO livers in the context of 3 weeks of CDA-HFD (Fig. S9C). The liver/body weight ratios were similar in control, *Pparα* KO, and *Cideb*; *Pparα* double KO mice, but all were greater than in *Cideb* single KO mice (Fig. 8D). Double KO mice had higher ALT, AST and triglyceride levels than control and single KO mice (Fig. 8E). Similarly, H&E staining showed extensive steatosis in control, *Pparα* KO, and *Cideb*; *Pparα* double KO mice, and all of these groups had substantially more steatosis than *Cideb* KO mice (Fig. 8F). CIDEB and PPARa were successfully reduced at the protein level, and *PPARα* deletion groups rescued or inhibited the downstream peroxisome associated protein increases caused by *Cideb* loss (Fig. 8G). This suggests that deleting *Cideb* upregulates the transcriptional activity of PPARα, promoting the expression of peroxisome- and FAO-related genes.

However, it was still unclear how loss of *Cideb* stimulated increased PPARα levels and transcriptional activity that promoted β-oxidation. We hypothesized that in *Cideb* KO livers, LD triglycerides undergo lipolysis, resulting in FFAs that act as ligands to activate PPARα. To test this, we asked if the inhibition of ATGL-mediated triglyceride lipolysis in hepatocytes could block the effects of *Cideb* deletion ^33,34^. To do this, we also generated control, *Cideb* single, *Atgl* single, or *Cideb*; *Atgl* double KO livers in the context of 3 weeks of CDA-HFD (Fig. S9C). The liver/body weight ratios were similar in control, *Atgl* KO, and *Cideb*; *Atgl* double KO mice. All of these groups had greater LW/BW ratios than *Cideb* single KO mice (Fig. 8H). Similarly, H&E staining showed extensive steatosis in control, *Atgl* KO, and *Cideb*; *Atgl* double KO mice, whereas *Cideb* single KO mice had minimal steatosis (Fig. 8I-J). Single *Atgl* KO livers showed the most steatosis. In *Cideb*; *Atgl* double KO mice, LDs were smaller but did not disappear as seen with single *Cideb* KO (Fig. 8I). This suggests that ATGL-mediated lipolysis contributes to the activation of PPARa in *Cideb* KO livers (Fig. 8K).

### *Cideb* loss prevents liver cancer development in the context of MASLD

Hepatocellular carcinoma (HCC) is one of the most devastating outcomes associated with MASH ^35^. Given that *Cideb* loss can promote clone expansion in MASH, we wanted to ask if this might eventually lead to increased cancer development. We used the diethylnitrosamine (DEN) plus WD approach to instigate tumor growth. 14-day-old *Cideb*^*fl/fl*^ mice were injected intraperitoneally with DEN. At 28 days of age, these mice were injected with either AAV-TBG-GFP or AAV-TBG-Cre and initiated on WD. After 8 months, more HCC was found on the liver surface of WT vs. hepatocyte-specific KO male mice (Fig. S10A), while there was only a non-significant difference in female mice (Fig. S10A). Microscopic tumor numbers were also lower in male KO livers (Fig. S10B,C).

## Discussion

Somatic and germline *CIDEB* mutations appear to ameliorate liver disease in humans ^1,2^. Here, functional characterization of all reported *CIDEB* somatic mutations showed that they impair CIDEB’s impact on LD growth. Furthermore, mosaic deletion of *Cideb* in the murine liver leads to positive selection of deleted clones, but specifically with certain fatty liver inducing diets. Interestingly, *Cideb* somatic mutations were more strongly selected for in the context of CDA-HFD compared with WD. Consistently, liver-specific *Cideb* deletion was associated with greater protection against CDA-HFD vs. WD. This suggests that *Cideb* directed interventions could be more effective in some subtypes of fatty liver disease. Future clinical trials in MASH may benefit from considering different mechanisms of MASH development, or by exploiting *Cideb* somatic mutations as biomarkers to predict the patients that would benefit the most.

Peng Li’s group have shown that whole-body *Cideb* deletion is protective against fatty liver disease and hyperlipidemia in mouse models ^3,7^. While they showed that CIDEB promotes VLDL lipidation and restrains β-oxidation ^3^, the predominant mechanism for MASLD protection was related to DNL. They showed that CIDEB promotes the loading of SREBP/SCAP into COPII vesicles, thereby facilitating SREBP1 transit from ER to nucleus ^5^. In this way, CIDEB promotes DNL transcription and its deletion impairs lipogenesis in the context of high fructose, low fat diets. While our study also shows anti-MASLD phenotypes, our results suggest that altered β-oxidation, rather than DNL, is predominantly responsible for protective phenotypes. The differences in the importance of DNL could be due to differences in the MASLD inducing diets used between studies, together with the fact that all other studies were done in whole-body *Cideb* KO mice, which also manifest a reduction in body weight. Since there are therapeutic approaches associated with DNL inhibition under clinical evaluation, it is important to delineate alternative approaches that are focused on β-oxidation.

Besides demonstrating that loss of function mechanisms underscore positive clonal selection in fatty livers, our studies also increase the mechanistic understanding of CIDEB function. *Cideb* KO hepatocytes have smaller LDs, an observation associated with reduced neutral lipids and cholesterol storage in the liver. In addition, there were significant decreases in unsaturated fatty acid content in the livers of *Cideb* KO mice fed with CDA-HFD, while saturated fatty acids remained largely unaffected. This suggests that CIDEB preferentially regulates the metabolism, storage, and FAO of unsaturated fatty acids in hepatocytes. *Cideb* loss reduces neutral lipid storage in LDs not through reduced DNL, but rather through increased lipolysis and β-oxidation. Since ATGL is the main triglyceride lipase in the liver, and because *Atgl* deletion rescues the effects of *Cideb* deletion, the reduction in LD size is potentially mediated through ATGL. Alternatively, the reduction in LD size caused by *Cideb* deletion could increase the surface area to volume ratio of LDs, thus increasing the efficiency of lipolysis by ATGL. Since unsaturated fatty acids can serve as ligands that activate PPARα’s transcriptional activity ^36–38^, we reason that increased unsaturated FFAs levels trigger increased β-oxidation through PPARα. Consequently, the enhanced expression of PPARα-regulated pathways facilitates the metabolism and consumption of FFAs in hepatocytes, thereby protecting the liver from the detrimental effects of unsaturated fatty acid accumulation. In conclusion, our findings show that targeting CIDEB may offer potential therapeutic strategies for MASLD, and our work suggests that a disease defining manifestation of *Cideb* mutation is increased β-oxidation.

## Materials and Methods

### Cloning

Human *CIDEA, CIDEB*, and *CIDEC* cDNAs were amplified by PCR using Phusion High-Fidelity DNA Polymerase and cloned into pCDNA3.1 either untagged or tagged with HA at the C-termini (Thermo Fisher, F530L). Truncations (stop), point mutants, and deletions (Δ) were generated using either QuikchangeII XL (Agilent Technologies) site-directed mutagenesis or Gibson assembly. WT and mutant versions of *CIDEA, CIDEB*, and *CIDEC* cDNAs were also inserted into a pEGFPN3 vector (Clontech, 6080-1) to create C-terminal GFP tagged constructs.

### Cell lines

Huh7, HepG2, and HeLa cells were cultured in High glucose Dulbecco’s Modified Eagle’s Medium (DMEM) supplemented with 10% (vol/vol) fetal bovine serum (FBS), and 1% penicillin-streptomycin at 37°C in 20% O_2_ and 5% CO_2_. H2.35 cells were cultured with complete DMEM supplemented with 4% FBS, 200 nM dexamethasone, and 1x penicillin-streptomycin.

### Western Blotting

Cells were lysed in radio-immunoprecipitation assay (RIPA) buffer (Fisher Scientific #PI89900) and supplemented with a protease and phosphatase inhibitor cocktail (ApexBio #K1007 and #K1012). Western blots were performed in the standard fashion using Bio-Rad reagents. The following antibodies were used: Phospho-Smad1/5 (CST #9516), Smad1 (CST #6944), actin (CST #4970), streptavidin-HRP (Life #S911), and anti-rabbit IgG, HRP-linked antibody (CST #7074).

### Animal models

All mice were housed and handled in strict accordance with the guidelines of the Institutional Animal Care and Use Committee (IACUC) at UT Southwestern. *Cideb*^*-/-*^ and *Cideb*^*fl/fl*^ mice, maintained on the C57BL/6 background, were generated by the CRI Mouse Genome Engineering Core. *Cideb*^*fl/fl*^ mice on a C57BL/6 background were crossed with *Alb-Cre* mice to achieve liver-specific embryonic deletion of *Cideb*, or with *LSL-tdTomato* mice for lineage tracing of *Cideb*-mutant cells. In general, experiments were initiated when mice were 6-8 weeks old. In most experiments, a high dose of AAV-TBG-Cre or AAV-TBG-GFP (5E10 GC/mouse) was administered to ensure efficient delivery to nearly all hepatocytes. Mouse sex is specified in the text or legends. For mosaic deletion of *Cideb* and Tomato labeling of hepatocytes, 0.125E10 genomic copies of AAV8-TBG-Cre in 100μl saline was injected retro-orbitally into *Cideb*^*fl/fl*^; *LSL-tdTomato* het or control *Cideb*^*+/+*^; *LSL-tdTomato* het mice at 8 weeks of age. After 3 months of treatment with NC, WD and CDA-HFD, mice were collected to determine the Tomato labeling percentage in the liver.

### Dietary models of MASLD and ALD

WD consisted of high sugar, high fat, and high cholesterol solid food (Teklad Diets #TD. 120528) combined with high-sugar water containing 23.1 g/L d-fructose (Sigma-Aldrich #G8270) and 18.9 g/L d-glucose (Sigma-Aldrich #F0127). CDA-HFD consisted of a methionine and choline-deficient high-fat diet (Research Diets #A06071302). The NIAAA alcohol feeding protocol is comprised of ethanol liquid diet (1-5%, vol/vol), and 1000 mL of ethanol liquid diet blended with 133 g of dry mix (Bio-serv, F1258SP) and different amounts of maltose dextrin and a different volume of water to the diet on the basis of the percentage of ethanol required ^23^ with weekly oral gavage of 5 μg/kg ethanol.

### Plasma and liver metabolic assays

Blood samples were collected in BD Microtainer Capillary Blood Collector and BD Microgard Closure (BD #365985) and centrifuged at 2000 g for 15 min at 4°C. The plasma collected after centrifugation was analyzed using VITROS MicroSlide Technology.

### Histology

Tissue samples were fixed overnight at 4°C in 4% paraformaldehyde. Fixed tissues were embedded in paraffin, sectioned and H&E staining by the UTSW Histopathology Core or the SCCC Tissue Management Service. Fibrosis detection was performed by staining with Sirius Red (IHC World #IW-3012) and quantified by ImageJ. Images were taken by a Hamamatsu Nanozoomer 2.0HT in the UTSW Whole Brain Microscopy Facility.

### MOSAICS screening and Cas9 mediated gene deletion in mice

SgRNAs (Table S1) were cloned into the MOSAICS-V8 or -V10 vectors, and the corresponding AAV8 was produced. For single gene deletion, AAV8 at a concentration of 2E11 VG/mL was diluted to a final volume of 100 μl with saline. This solution was retro-orbitally injected into *Rosa-rtTA; TetO-Cas9* double homozygous mice (iCas9) 3-5 days after initiating doxycycline (dox) water (1 mg/mL dox) at 6.5 weeks of age. For the AAV libraries used in the LD screens, AAV8 at a concentration of 5E11 VG/mL was injected. For liver-wide single or double gene deletion, we used AAV8 at a concentration of 1E12 VG/mL. One week post-injection, the mice were fed NC, WD, or CDA-HFD. The reads from the sequencing of amplicon libraries were processed using cutadapt (version 1.9.1) to remove excessive adaptor sequences, retaining only the sgRNA sequences. The 5’ sequences were trimmed using the options `-O 32 –discard-untrimmed -g CTTTATATATCTTGTGGAAAGGACGAAACACCGˋ, and the 3’ sequences were trimmed using the options `-O 12 -a GTTTTAGAGCTAGAAATAGCAˋ. The abundance of each sgRNA was calculated with the count function in MAGeCK (version 0.5.6) using default options. The trimmed fastq files were assigned to NC, WD, and CDA-HFD groups and uploaded, along with library files containing sgRNA sequences and targeted gene names, to a server preloaded with MAGeCK. The enrichment of each sgRNA was calculated using the test function in MAGeCK (Table S2).

### Genomic DNA extraction, sgRNA amplification, and amplicon library construction

To extract genomic DNA containing the integrated sgRNA, the entire liver was minced into approximately 1 mm^3^ pieces using a blade and weighed. Minced liver was transferred into a glass Wheaton Dounce Tissue Grinder in two volumes (w/v) of homogenizing buffer (100 mM NaCl, 25 mM EDTA, 0.5% SDS, 10 mM Tris-HCl, pH 8) and stroked 50 times or until no bulk tissue remained. After homogenizing, 500 μl liver lysate was transferred to a 15 mL tube for genomic DNA extraction using the Blood & Cell Culture DNA Midi Kit (Qiagen #13343) according to the manufacturer’s protocol. The remaining lysates were frozen at -80°C as backup samples. For sgRNA amplification and amplicon library construction, 5 μg of genomic DNA, 5 μl of general forward primer mix (5 mM), 5 μl of barcode-specific reverse primer (5 mM), 1 μl of Q5 DNA polymerase, 10 μl of Q5 buffer, 10 μl of High GC buffer, 1 μl of dNTP (10 mM), and water were mixed for a 50 μl PCR reaction. Two reactions were made for each genomic DNA sample. The PCR cycle was: 95°C for 3 min, followed by (95°C for 30 s, 60°C for 30 s, and 72°C for 20 s) repeated n times, and a final extension at 72°C for 2 min. The cycle number was pre-optimized using the same PCR reactions with a smaller volume, choosing the cycle number that gave a weak but sharp band on a DNA gel. For the final PCR reaction, 25 cycles were used. After PCR, 50 μl was resolved on a DNA gel. The 250 bp band corresponding to the amplicon was cut and purified using the QIAquick Gel Extraction Kit (Qiagen #28704). DNA concentration was determined using the Qubit kit (Invitrogen #Q32853), and high-throughput sequencing was performed using an Illumina NextSeq500 system at the CRI at UT Southwestern Sequencing Facility ^16,42^ .

### RNA extraction and real time qPCR

Total RNA was isolated from liver tissue using TRIzol reagent (Invitrogen #15596018), followed by purification with the RNeasy Mini kit (Qiagen #74104). For real-time quantitative PCR, cDNA synthesis was performed from 1 μg of total RNA using the iScript Reverse Transcription Supermix (BioRad #1708840) in a 20 μL reaction. Each resulting cDNA sample was then diluted to a final volume of 200 μL. For PCR, 2 μL of this diluted cDNA was combined with specific primers and iTaq Universal SYBR Green Supermix (BioRad #1725121) in a 10 μL reaction (Table S1).

### RNA-seq analysis

RNA was purified as described. The RNA was further treated with DNase (Invitrogen #AM1906). The RNA-seq library was prepared using the Nugen Ovation Mouse RNA-seq System (Nugen #0348-32) and quantified with the Qubit dsDNA BR Assay kit. Herein, 75 bp single-end sequencing was performed on an Illumina NextSeq 500 at the Children’s Research Institute Sequencing Facility. RNA-seq data were deposited in the GEO database (GSE294945). Table S3 contains the differentially expressed genes.

### Poloxamer-407 assay

Mice were fasted overnight before the VLDL secretion assay. After baseline blood collection (~50 μl) from the retro-orbital sinus, mice were intraperitoneally injected with poloxamer-407 (P-407) (10% (w/v)) in saline (Sigma-Aldrich, #16758) at a dose of 1.0 g/kg of body weight. Blood samples (approximately 50 μL) were collected at 0, 90, 180 min post-injection, and plasma triglycerides were measured as described above.

### Statistical analysis

The data reflect multiple experiments performed on different days using mice derived from different litters. In all experiments, mice were not excluded from analysis after the experiment was initiated unless the mice died. Unless otherwise stated in the methods or figure legends, 2-tailed Student’s *t* tests (2-sample equal variance) were used to test the significance of differences between 2 groups. For time-course–expression experiments, 1-way ANOVA was performed and the significance shown was compared with the initial time point. For experiments involving 2 groups and different time points, 2-way ANOVAs were used, and the significance was compared with the means of each group at different time points. Variation is indicated using SEM and presented as mean ± SEM. Statistical significance was defined as *P* < 0.05.

### Liver cancer modeling

For the mutagen HCC model, (50 μg/g of DEN (Sigma #N0756) was given at p14 to both male and female mice. At 28 days of age, these mice were injected with either AAV-TBG-GFP or AAV-TBG-Cre and initiated on WD. After 8 months, livers were harvested.

## Supplementary Material

Supplementary Materials

## Data Availability

The RNA-seq data in this study has been deposited in the Gene Expression Omnibus (GEO) under accession number GSE294945, and are publicly available at https://www.ncbi.nlm.nih.gov/geo/query/acc.cgi?acc=GSE294945. The mouse models are available upon request and after MTAs are generated with UTSW.

## Highlights

- ***Cideb* deficient clones are positively selected with a subset of MAFLD inducing diets**.
- ***Cideb* deletion suppresses liver disease caused by CDA-HFD, WD, and alcohol**.
- ***Cideb* deletion protects against MASLD in part through increased hepatic fatty acid oxidation**.
