# Supplementary material for: Mechanistic basis for protection against fatty liver disease by CIDEB loss-of-function mutations
Source: J Hepatol. Author manuscript; Available in PMC 2026 Jan 1. (PMC12313214; doi:10.1016/j.jhep.2025.06.021)
Supplement: Supplementary Materials [file EMS206755-supplement-Supplementary_Materials.pdf]

Title: Mechanistic basis for protection against fatty liver disease by *CIDEB* loss-of-function mutations

Short Title: CIDEB loss protects against fatty liver

Authors:
Qiyu Zeng,[1] Satish Patel,[2] Xun Wang,[1] Meng-Hsiung Hsieh,[1] Zhijie Li,[1] Xiongzhao Ren,[1] Jingjing Wang,[1] Dohun Kim,[1] Shili Li,[1] Xinping Gu,[1] Greg Mannino,[1] Gianna Maggiore,[1] Xiangyi Fang,[1] Lin Li,[1] Min Zhu,[1] Mengmeng Wang,[1] Boyuan Li,[1] Amaey Bellary,[1] Koini Lim,[2] Zhetuo Qi,[2] Pushpa Pushpa,[2] Mandour Omer Mandour,[2] Vladimir Saudek,[2] Tripti Sharma,[1] Yu Zhang,[1] Gerta Hoxhaj,[1] Prashant Mishra,[1] Purva Gopal,[3] Peter Campbell,[4] Matthew Hoare,[5] David B. Savage,[2] Hao Zhu.[1]

Affiliations:
[1]Children's Research Institute, Departments of Pediatrics and Internal Medicine, Center for Regenerative Science and Medicine, Children's Research Institute Mouse Genome Engineering Core, University of Texas Southwestern Medical Center, Dallas, TX 75390, USA.
[2]University of Cambridge Metabolic Research Laboratories, Wellcome Trust-MRC Institute of Metabolic Science, Cambridge, CB2 0QQ, UK.
[3]Department of Pathology, University of Texas Southwestern Medical Center, Dallas, TX 75390, USA.
[4]Cancer Genome Project, Wellcome Sanger Institute, Hinxton, Cambridgeshire CB10 1SA, UK.
[5]University of Cambridge Department of Medicine, Cambridge Biomedical Campus, Cambridge, CB2 0QQ, UK and University of Cambridge Early Cancer Institute, Hutchison Research Centre, Cambridge Biomedical Campus, Cambridge, CB2 0XZ, UK.

*Co-correspondence and lead contact:
David Savage (dbs23@cam.ac.uk) and Hao Zhu (Hao.Zhu@utsouthwestern.edu)

Supplementary Method

High content confocal imaging and quantification of LD size. HeLa cells were seeded at a density of $1x10^5$ cells/well onto ethanol-treated glass coverslips in 12-well plates or 6000 cells/well onto Perkin Elmer 96-Well Phenoplate Ultra Plates (6055302; Perkin Elmer). The following day, *CIDEA*, *CIDEB,* or *CIDEC* cDNA was co-transfected along with GFP using Lipofectamine LTX according to the manufacturer's instructions. 4 h later, the media was replaced with fresh DMEM supplemented with 400 µM Oleic acid. 24 h post-transfection, the media was removed and cells were washed three times with 1x PBS and fixed with 4% (v/v) formaldehyde in PBS for 15 min at room temperature followed by three washes for 5 min with PBS. LDs were visualized either by addition of 1:2000 dilution of BODIPY™ 558/568 C_{12} during oleate-loading or stained with 1x HCS LipidTOX Deep Red Neutral Lipid Stain post-fixation for one hour. Imaging was carried out on the Perkin Elmer Opera Phenix spinning disc confocal microscope equipped with a 40x/1.1 Water objective with 2-pixel binning. EGFP and LipidTox Deep Red Neutral Stain were excited at 488 and 640, and emission signals were collected at 500-550 nm and 650-760 nm, respectively. Images were acquired and LD volumes in GFP positive cells were determined by the Harmony phenoLOGIC Software (v4.9; Perkin Elmer). LD enlargement activity is represented as Z-scores where data is normalised as $x\text{-}\mu/\sigma$ where μ was calculated from wild type (WT) CIDE or empty vector (EV) and σ from all values.

For HA immunostaining, fixed cells were permeabilized with 0.5% saponin for 10 min, washed with PBS, blocked with 1% BSA in PBST for 1h, followed by overnight incubation at 4C with 1:500 HA-Tag (C29F4) antibody. Cells were washed four times for 5min with 0.1% BSA in PBST and incubated with Alexa Fluor 488-conjugated secondary antibody. After washing with PBS, cells were mounted using VECTASHIELD Antifade Mounting Medium (Vector Laboratories, H-1000) or Prolong Gold Antifade Mountant with DAPI (Thermo Fisher, P36931). 2-dimensional images were acquired using a Leica SP8 confocal microscope. HA (or GFP) and LipidTOX Deep Red Neutral Lipid Stain were excited at 488 and 637 nm, and emission signals were collected at 495–535 and 645–700 nm, respectively. Bodipy 558/568 C_{12} was excited at 558nm and emission collected 565-600 nm.

CIDEB structural modeling. AlphaFold predictions were carried out with AlphaFold2 [39] using the protocol developed for oligomers [40] on the Google ColabFold v1.5.5 server. The quality of the prediction is described by the two main AlphaFold descriptors: the predicted local distance difference test (pLDDT) and predicted aligned error (PAE). The calculations were run for structures consisting of up to 12 monomeric chains. Only tetramers folded in the stable structure, reaching pLDDT 80-95 (confident to very high reliability). PAE descriptor indicates that all four protomers interact tightly in the tetramer (**Fig. S1**). Predictions of other oligomers led to structures of very low pLDDT (<40) and were discarded. The predictions of a tetramer were also run for human CIDEA and CIDEB and the most remote available sequence of hagfish CIDEB (UniProt entry A0A8C4N4T1) leading to very similar structures with root mean square errors of superposition of the b-sheet ranging from 1.9 to 2.4 to Å and comparable quality descriptors.

Primary hepatocyte isolation and culture. Primary hepatocytes were isolated from mouse livers using a two-step collagenase perfusion method. Briefly, mice were anesthetized with isoflurane, and the abdominal area was sterilized with 70% ethanol. A midline incision was made to expose the portal vein, and a catheter was inserted into the inferior vena cava (IVC) and secured by threading a needle through the catheter's butterfly wing. Perfusion was initiated with Liver Perfusion Medium (Life Technologies, #17701038) at a flow rate of 3 mL/min, using approximately 30–50 mL of medium. The portal vein was immediately severed to allow for adequate outflow. Following perfusion with Liver Perfusion Medium, the liver was perfused with Liver Digestion Medium (Life Technologies, #17703034) at the same flow rate, using an additional 30–50 mL. After digestion, the liver was carefully removed and placed in 10 mL of Liver Digestion Medium in a 10 cm culture dish. The liver capsule was gently peeled away, and the tissue was gently agitated to release hepatocytes. The cell suspension was then filtered through a 70 µm nylon mesh into a 50 mL tube prefilled with 20 mL of Hepatocyte Washing Medium (Life Technologies #17704024) on ice to halt enzymatic activity. Cells were pelleted by centrifugation at 50 g for 5 minutes at 4°C, and the supernatant was discarded. The cell pellet was resuspended in 20 mL of Washing Medium, and the wash step was repeated twice to ensure cell purity. Cell viability and yield were assessed by Trypan blue exclusion. Cells were then centrifuged once more at 50 g for 5 minutes and resuspended in the desired volume of washing medium or buffer for further experiments.

FFA treatment assay. Human liver cancer cell lines (HepG2 and Huh7), a mouse immortalized liver cell line (H2.35), and primary hepatocytes (*Cideb*$^{fl/fl}$ and *Alb-Cre*; *Cideb*$^{fl/fl}$) were cultured and treated with saturated and unsaturated fatty acids. Cells were exposed to fatty acids at a final concentration of 100 µM for 16 hours. Saturated fatty acids included palmitic acid (16:0) (Sigma Aldrich #43051), stearic acid (18:0) (Sigma Aldrich #85679), and arachidic acid (20:0) (Sigma Aldrich #A3631). Unsaturated fatty acids included oleic acid (18:1) (Sigma Aldrich #O1257), linolenic acid (18:3) (Sigma Aldrich #L1376), stearidonic acid (18:4) (Sigma Aldrich #SMB00291), mead acid (20:3) (Sigma Aldrich #43059), arachidonic acid (20:4) (Sigma Aldrich #23401), and eicosapentaenoic acid (20:5) (Sigma Aldrich #E3635). All fatty acids (50 mM) were dissolved in ethanol, then diluted to 5 mM with warm 10% fatty acid-free BSA (Sigma Aldrich #A8806) prior to application to cells to ensure optimal cellular uptake. After incubation, cells were harvested for further analysis as described in subsequent sections.

AAV production and purification. AAV-Pro 293T cells (Takara #632273) were plated one day before transfection at 50% confluence, allowing the cells to reach 80-90% confluence the next day. For transfection of one 15 cm dish, the following plasmids were mixed in one tube with 1 mL Opti-MEM medium: 10 µg MOSAICS-V8 or -V10 vector, 10 µg pAAV2/8 (Addgene #112864), and 20 µg pAdDeltaF6 (Addgene #112867). In another tube, 160 µl PEI solution (1 mg/mL in water, pH 7.0, ChemCruz #sc-360988) was mixed with 1 mL Opti-MEM medium. The solutions from both tubes were then combined and incubated for 10 minutes before being added to the cell culture. Forty-eight hours after transfection, the cells were scraped off the dish and collected by centrifugation at 500 g for 10 minutes. The supernatant was disinfected and discarded, and the cell pellets were lysed in 1.5 mL lysis buffer per 15 cm dish (PBS supplemented with NaCl to a final concentration of 200 mM and CHAPS to a final concentration of 0.5% (w/v)). The cell

suspension was placed on ice for 10 minutes with intermittent vortexing, and then centrifuged at 20,000 g for 10 minutes at 4°C. The supernatant containing the AAV was collected. To set up the gravity column for AAV purification, 0.5 mL of AAV8-binding slurry beads (ThermoFisher #A30789) was loaded into an empty column (Bio-Rad #731-1550). After the beads were tightly packed at the bottom, they were washed with 5 mL wash buffer (PBS supplemented with NaCl to a final concentration of 500 mM). The supernatant containing AAV was then loaded onto the column. After all the supernatant flowed through, the beads were washed twice with 10 mL wash buffer. The AAV was then eluted with 3 mL elution buffer (100 mM glycine, 500 mM NaCl in water, pH 2.5) and the eluate was immediately neutralized with 0.12 mL 1 M Tris-HCl (pH 7.5-8.0). The AAV was concentrated by centrifugation at 2000 g for 3-5 minutes at 4°C using a 100k Amicon Ultra Centrifugal Filter Unit (Millipore #UFC810024). After centrifugation, the volume of AAV was adjusted to equal or less than 0.5 mL. The concentrated AAV was diluted with 4-5 mL AAV dialysis buffer (PBS supplemented with NaCl to a final concentration of 212 mM and 5% sorbitol (w/v)) and centrifuged at 2000 g for 3-5 minutes at 4°C. The dilution and centrifugation processes were repeated three times. The final concentrated AAV was transferred into a 1.5 mL tube and centrifuged at 20,000 g for 5 minutes to remove debris. The supernatant was aliquoted, flash frozen using liquid nitrogen, and stored at -80°C. The AAV titer was determined using the AAVpro Titration Kit (for Real Time PCR) Ver.2 (Takara Bio #6233).

Poloxamer-407 assay. Mice were fasted overnight before the VLDL secretion assay. After baseline blood collection (~50 μl) from the retro-orbital sinus, mice were intraperitoneally injected with poloxamer-407 (P-407) (10% (w/v)) in saline (Sigma-Aldrich, #16758) at a dose of 1.0 g/kg of body weight. Blood samples (approximately 50 μL) were collected at 0, 90, 180 min post-injection, and plasma triglycerides were measured as described above.

FFA analysis. Approximately 20 mg of frozen liver tissue was homogenized in a 2:1:1 mixture of chloroform, PBS, and methanol. After homogenization, samples were centrifuged in screw-cap glass vials at 1,000 g at 4°C for 10 minutes. The chloroform layer was transferred to a new vial and dried under nitrogen. Dried samples were saponified with 1 M KOH in 80% methanol at 56°C for 1 hour, neutralized with HCl, and re-dried under nitrogen. Samples were then re-suspended in a 65:35 isopropanol/methanol mixture for LC-MS/MS analysis. Fatty acids were resolved on a Thermo Scientific Vanquish Flex UHPLC system with an Accucore C18 column (2.1x150 mm), using a binary solvent system (A: 50:50 water:acetonitrile; B: 88:10:2 isopropanol:acetonitrile:water with 5 mM ammonium formate and 0.1% formic acid). A programmed gradient flow (0.17 mL/min) separated fatty acids, with the column at 60°C. Mass spectrometry was performed on a Thermo Fusion Lumos Tribrid Orbitrap, using MSn acquisition for structural identification of lipid species [27]. Data were analyzed with TraceFinder software (Thermo Fisher Scientific, V5.0) using a validated library of fatty acid spectra.

Isotope tracing to assess DNL and FAO. Mice received an intraperitoneal injection of 99.9% D_2O (Sigma-Aldrich, 151882) in 0.9% NaCl at 30 μL/g body weight. After 5 hours, mice were sacrificed and ~20 mg liver samples were homogenized in a 2:1:1 mixture of

chloroform, PBS, and methanol, then centrifuged at 1,000 g at 4°C for 10 minutes. The chloroform layer was dried under nitrogen, saponified with 1 M KOH in 80% methanol at 56°C for 1 hour, neutralized with HCl, re-dried, and re-suspended in a 65:35 isopropanol/methanol mixture for analysis. Mass spectrometry was performed on a Thermo Fusion Lumos Tribrid Orbitrap. Extracted ion chromatograms for labeled and non-labeled species were generated with 1-5 ppm mass accuracy to distinguish ^{13}C and ^{2}H isotopes. Data analysis used TraceFinder software (V5.0), with isotope correction via a customized R script based on the AccuCor algorithm. For FAO, isotope tracing was conducted with [U-^{13}C]linolenic acid or [U-^{13}C]stearic acid (Cambridge Isotope Laboratories, CLM-6990-PK and CLM-8386-1) dissolved in ethanol at 50 mM, diluted to 5 mM with warm 10% fatty acid-free BSA. Mice were infused at 1 mg/min/kg for 2 hours. Approximately 20 mg of frozen liver tissue was homogenized in 80:20 acetonitrile:water, subjected to freeze-thaw cycles, and centrifuged at 16,000 g for 15 minutes at 4°C. The supernatant was analyzed on a Thermo Scientific QExactive HF-X hybrid quadrupole orbitrap HRMS [28]. Data analysis was performed with TraceFinder software (V5.0), with isotope abundances corrected via a customized R script based on the AccuCor algorithm [43].

The fatty acid oxidation assay. The fatty acid oxidation assay was conducted using [1-^{14}C]-stearic acid isotope tracing in hepatocytes cultured to 90% confluence in 6-well plates. Cells were incubated in DMEM containing 10% FBS, 50 μM ^{12}C-stearic acid (Sigma Aldrich #85679), 2 μCi [1-^{14}C]-stearic acid (American Radiolabeled Chemicals, ARC0258), 0.28% BSA (Goldbio, A-421-10), and 1 mM L-carnitine (Millipore Sigma, C0283). Prior to incubation, [1-^{14}C]-stearic acid was dried at 37°C overnight and conjugated with BSA and 12C-stearic acid. Hepatocytes were exposed to this mixture for 6 hours. Released $^{14}CO_2$ was captured on filter paper pre-soaked with 30 μl of 1N NaOH for 2 hours. The filter paper was then transferred to a scintillation vial (VWR, 66022-055) for analysis. The CPM value was measured for 1 minute using a scintillation counter (Beckman Coulter LS 6500 Liquid Scintillation Counter). For experiments involving linolenic acid (American Radiolabeled Chemicals, ARC0379), the same protocol was followed.

Statistical analysis. The data reflect multiple experiments performed on different days using mice derived from different litters. In all experiments, mice were not excluded from analysis after the experiment was initiated unless the mice died. Unless otherwise stated in the methods or figure legends, 2-tailed Student's *t* tests (2-sample equal variance) were used to test the significance of differences between 2 groups. For time-course–expression experiments, 1-way ANOVA was performed and the significance shown was compared with the initial time point. For experiments involving 2 groups and different time points, 2-way ANOVAs were used, and the significance was compared with the means of each group at different time points. Variation is indicated using SEM and presented as mean ± SEM. Statistical significance was defined as $P < 0.05$.

Liver cancer modeling. For the mutagen HCC model, (50 μg/g of DEN (Sigma #N0756) was given at p14 to both male and female mice. At 28 days of age, these mice were injected with either AAV-TBG-GFP or AAV-TBG-Cre and initiated on WD. After 8 months, livers were harvested.

Fig. S1. Somatic *CIDEB* mutations in patients with chronic liver disease.
Distribution of somatic mutations in *CIDEB*. Amino acids with observed mutations in chronic liver disease shown above the WT protein sequence.

Fig. S2. Disruption of the predicted C-terminal β-sheet and N-terminus helix on CIDEC lipid droplet enlargement activity.

- **(A)** Quantification of LD volume in oleate-loaded HeLa cells transfected with CIDEC β-sheet deletion and point mutants. Enlargement activity is represented as a Z-score where 0 is WT CIDEC.
- **(B)** Representative images of LDs (red) and HA (green) in oleate-loaded HeLa cells transfected with CIDEC β-sheet deletion and point mutants. Nuclei were stained with DAPI (blue).
- **(C)** Western blot analysis in oleate-loaded HeLa cells transfected with CIDEC β-sheet deletion and point mutants.
- **(D)** Sequence alignment of the β-sheet region of CIDE proteins from representative vertebrates. β-sheet strands from AlphaFold predicted tetramers are indicated by green arrows. The position of the hydrophobic residues within the β-sheet (indicated by arrows), the mutation of which to acidic D impaired LD growth. A, B, and C stand for CIDEA, CIDEB and CIDEC, respectively.
- **(E)** Cluster of aromatic residues inside the CIDEB putative channel (hollow β-barrel).
- **(F)** C164 in two antiparallel protomers of the CIDEB channel can form disulfide bonds. Side view (left panel) and along the axis (right panel) of the channel.
- **(G)** AlphaFold prediction of the N-terminal segment of CIDEB aa 1-34 with quality parameters coloured as in **Fig. 2A**.
- **(H)** Surface representation of the helix, predicted with confidence, viewed from two sides and coloured according to residue type (yellow: hydrophobic, red: negative, blue: positive, violet: others).
- **(I)** Sequence alignment of the N-terminal region of CIDE proteins from representative vertebrates. The α-helix predicted by AlphaFold in human CIDEB is indicated by a yellow cylinder. The insertion between positions 16-28 (blue line), unique to B_Hagfish, is not shown.

Data are presented as mean ± SEM. Statistical analysis was performed using one-way ANOVA followed by Tukey's post hoc test. ****$p < 0.0001$.

Fig. S3. *CIDEB* expression in different MAFLD models.

- **(A)** Body weight, liver weight, and liver/body weight ratio in C57BL/6 mice fed NC, WD, or CDA-HFD for 12 weeks.
- **(B)** Representative H&E staining of livers treated with NC, WD, or CDA-HFD for 12 weeks.
- **(C)** Western blot analysis to assess CIDEB levels in the liver.
- **(D)** Quantification of CIDEB protein levels.
- **(E)** qPCR for *Cideb*, *Cidea*, and *Cidec* mRNA in livers fed with NC, WD, or CDA-HFD.

Data are presented as mean ± SEM. Statistical analysis was performed using one-way ANOVA followed by Tukey's post hoc test. *$p < 0.05$, **$p < 0.01$, ****$p < 0.0001$.

Fig. S4. In vivo CRISPR screening of genes involved in fatty liver disease.

- **(A)** Genes targeted in the CRISPR screen.
- **(B)** sgRNAs enriched in CDA-HFD or WD but not in NC-fed livers. Each circle represents one sgRNA. Different sgRNAs targeting the same genes were aligned vertically. Circle sizes correlate to $-\log_2(p)$. Control sgRNAs are filled black circles.

Fig. S5. Generation and validation of *Cideb* KO mice.

- **(A)** Schematic representation of the mouse *Cideb* gene and overlap with the *Nop9* gene.
- **(B)** Strategy to generate whole-body *Cideb* KO and floxed mice.
- **(C)** CIDEB protein in WT and whole-body KO livers.
- **(D)** CIDEB protein in livers from $Cideb^{fl/fl}$ mice injected with AAV-TBG-GFP or AAV-TBG-Cre.

Fig. S6. Impact of hepatocyte-specific *Cideb* deletion in female mice.

- **(A)** Body weight, liver weight, and liver-to-body weight ratio in mice injected with AAV-TBG-GFP or AAV-TBG-Cre and fed with 24 weeks of WD.
- **(B)** Plasma ALT, AST, cholesterol, and triglycerides in WD fed mice.
- **(C)** H&E staining of livers from **Fig. S6A**.
- **(D)** Body weight, liver weight, and liver-to-body weight ratio in mice injected with AAV-TBG-GFP or AAV-TBG-Cre and fed with 12 weeks of CDA-HFD.
- **(E)** Plasma ALT, AST and triglycerides in CDA-HFD fed mice.
- **(F)** H&E staining of livers from **Fig. S6D**.
- **(G)** Sirius red staining of livers from **Fig. S6D**.
- **(H)** NAFLD activity score of the H&E sections from liver exposed to WD. The right panel represents the total NAFLD activity score, which is the sum of the four scores on the left (Each dot represents the average NAS score from 1–3 liver sections within a single slice).
- **(I)** NAFLD activity score of the H&E sections from liver exposed to CDA-HFD. The right panel represents the total NAFLD activity score, which is the sum of the four scores on the left (Each dot represents the average NAS score from 1–3 liver sections within a single slice).

Data are shown as mean ± SEM. p-values were calculated by unpaired two-tailed Student's t-test. *$p < 0.05$, **$p < 0.01$, ***$p < 0.001$, ****$p < 0.0001$, ns, not significant.

Fig. S7. Unsaturated fatty acids preferentially accumulate within LDs in vitro and in vivo. .

- **(A)** Lipidomic analysis of the fatty acid content of WD and CDA-HFD.
- **(B)** Lipidomic analysis of saturated and unsaturated fatty acids in WT livers exposed to WD or CDA-HFD for 12 weeks.
- **(C)** Representative images of LD staining (green) of HepG2, Huh7, and H2.35 cells treated with 10% BSA, saturated (16:0, 18:0, 20:0) or unsaturated fatty acids (18:1, 18:3, 18:4, 20:3, 20:4, 20:5) at a concentration of 100 µM for 16 hours. LDs were

stained with Bodipy (green) and nuclei were stained with Hoechst (blue) (scale bars = 20 µm).

(D) Quantification of the LD area (%) in the cells from C under fatty acid treatments (n = 3 individual images from the same well).

(E) Representative images of LD staining (green) of C57BL/6 primary hepatocytes treated with various lipids, same as in **Fig. S7C**.

(F) Quantification of the LD area (%) from **Fig. S7E** (n = 3 individual images from the same well).

Data are presented as mean ± SEM. Statistical analysis was performed using one-way ANOVA followed by Tukey's post hoc test. *p < 0.05, **p < 0.01, ***p < 0.001, ****p < 0.0001.

Fig. S8. *Cideb* deletion reduces unsaturated fatty acids and LD growth in the liver.

(A) Relative abundance of fatty acids in *Cideb*$^{fl/fl}$ livers injected with AAV-TBG-GFP or AAV-TBG-Cre and fed with 12 weeks of CDA-HFD.

(B) Representative LD staining (green) of primary hepatocytes from *Cideb*$^{fl/fl}$ and *Alb-Cre; Cideb*$^{fl/fl}$ mice (scale bars = 20 µm).

(C) Quantification of LD area from **Fig. S8B**.

(D) qPCR of *Gdf15* and *Fgf21* from WT and whole body KO livers of CDA-HFD treated mice.

Data are shown as mean ± SEM. p-values were calculated by unpaired two-tailed Student's t-test. *p < 0.05, **p < 0.01, ***p < 0.001, ****p < 0.0001.

Fig. S9. *Cideb* deletion alters liver gene expression in the context of MAFLD inducing diets.

(A) Gene Set Enrichment Analysis (GSEA) of RNA-seq data from 12 week CDA-HFD treated *Cideb*$^{fl/fl}$ livers injected with either AAV-TBG-GFP or AAV-TBG-Cre.

(B) GSEA of RNA-seq data from 24 week WD treated *Cideb*$^{fl/fl}$ livers injected with either AAV-TBG-GFP or AAV-TBG-Cre.

(C) qPCR of *Cideb, Pparα* and *Atgl* from iCas9 mice given high-dose AAV-sgRNAs (*sgCtrl*, *sgCideb*, *sgPparα*, *sgCideb*/*sgPparα, sgAtgl or sgCideb/sgAtgl*) treated with CDA-HFD for 3 weeks (n=6, 5, 8, 9, 9 and 8 mice).

Data are presented as mean ± SEM. Statistical analysis was performed using one-way ANOVA followed by Tukey's post hoc test. *p < 0.05, **p < 0.01, ***p < 0.001, ****p < 0.0001.

Fig. S10. *Cideb* deletion reduces liver tumor development in male mice.

(A) Surface tumor numbers in DEN-treated livers. *Cideb* was deleted using AAV-TBG-Cre at P28.

(B) Representative picture and H&E staining of DEN treated livers from **Fig. S10A**.

(C) Quantification of tumor nodules.

Data are shown as mean ± SEM. p-values were calculated by unpaired two-tailed Student's t-test. **p < 0.01.

Figure S1

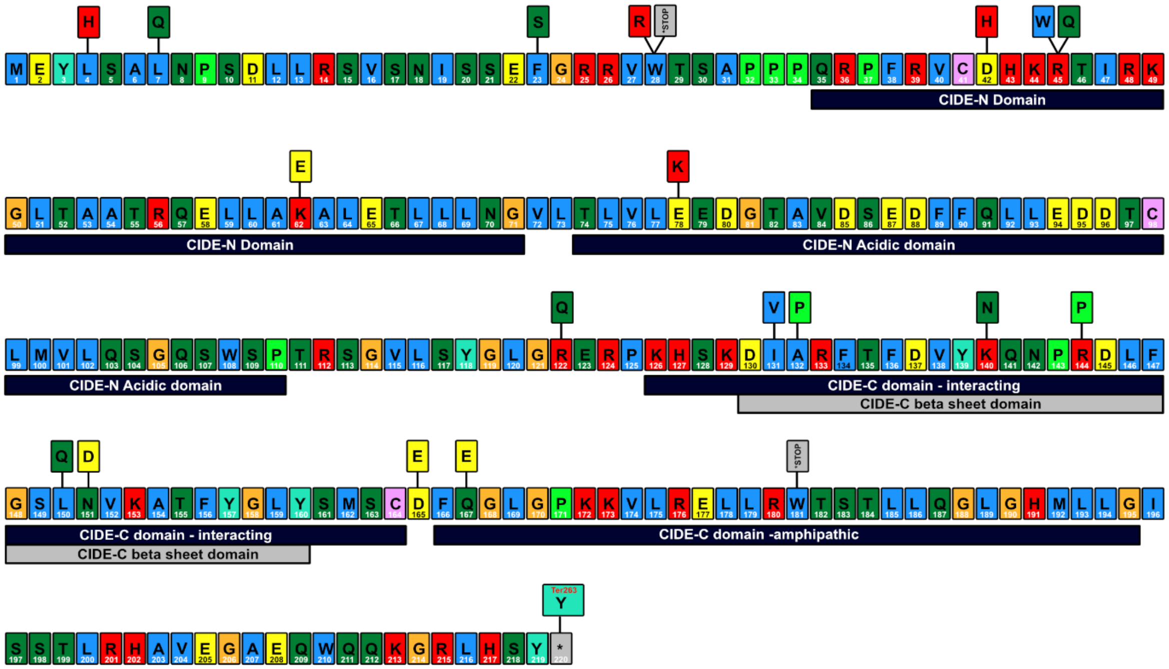
CIDE-N Domain
CIDE-N Domain
CIDE-N Acidic domain
CIDE-N Acidic domain
CIDE-C domain - interacting
CIDE-C beta sheet domain
CIDE-C domain - interacting
CIDE-C beta sheet domain
CIDE-C domain -amphipathic

Figure S2

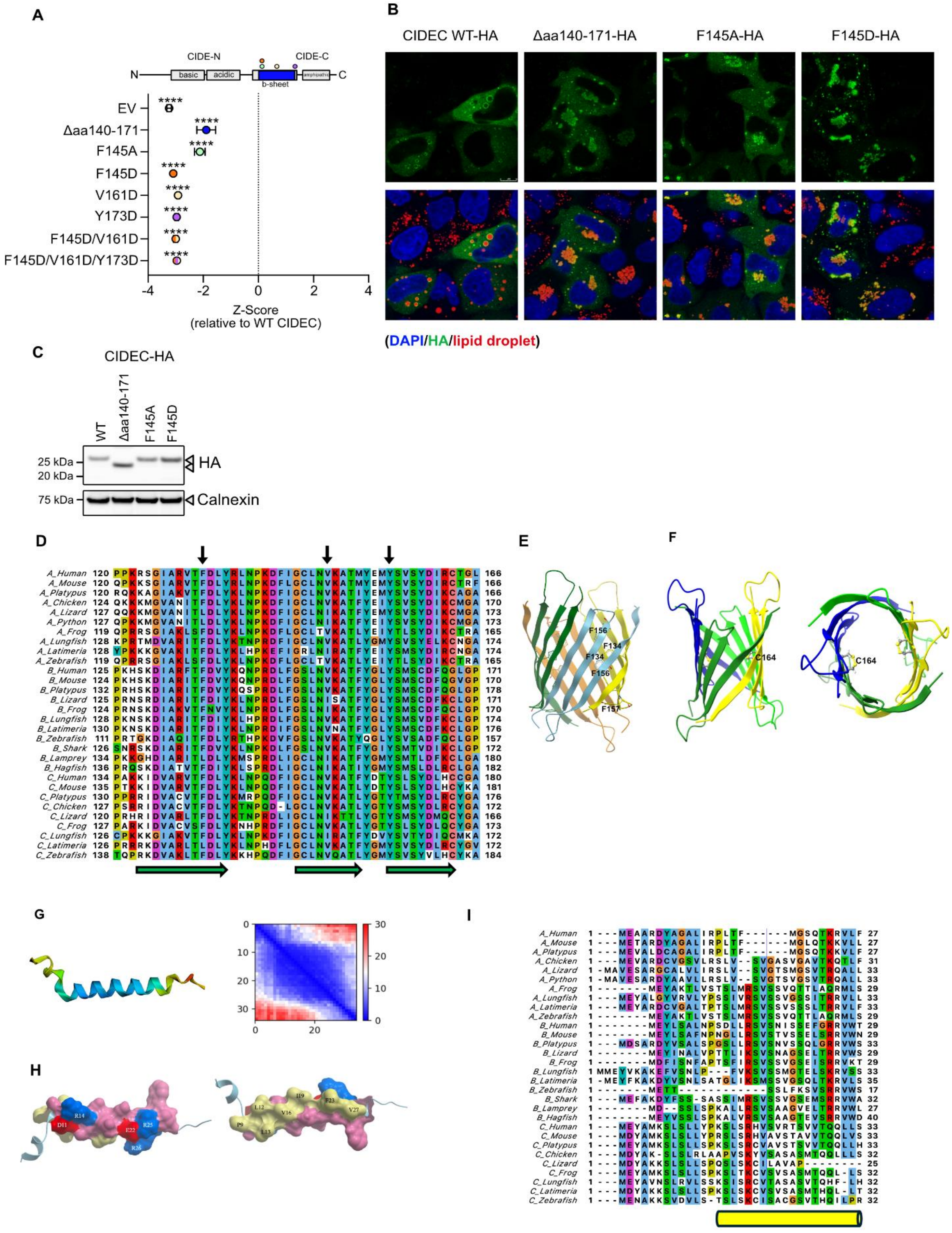
A
CIDE-N
CIDE-C
N
basic
acidic
b-sheet
C
EV
Δaa140-171
F145A
F145D
V161D
Y173D
F145D/V161D
F145D/V161D/Y173D
****
-4
-2
0
2
4
Z-Score
(relative to WT CIDEC)
B
CIDEC WT-HA
Δaa140-171-HA
F145A-HA
F145D-HA
(DAPI/HA/lipid droplet)
C
CIDEC-HA
WT
Δaa140-171
F145A
F145D
25 kDa
20 kDa
HA
75 kDa
Calnexin
D
E
F156
F134
F134
F156
F157
F
C164
C164
G
H
I

Figure S3

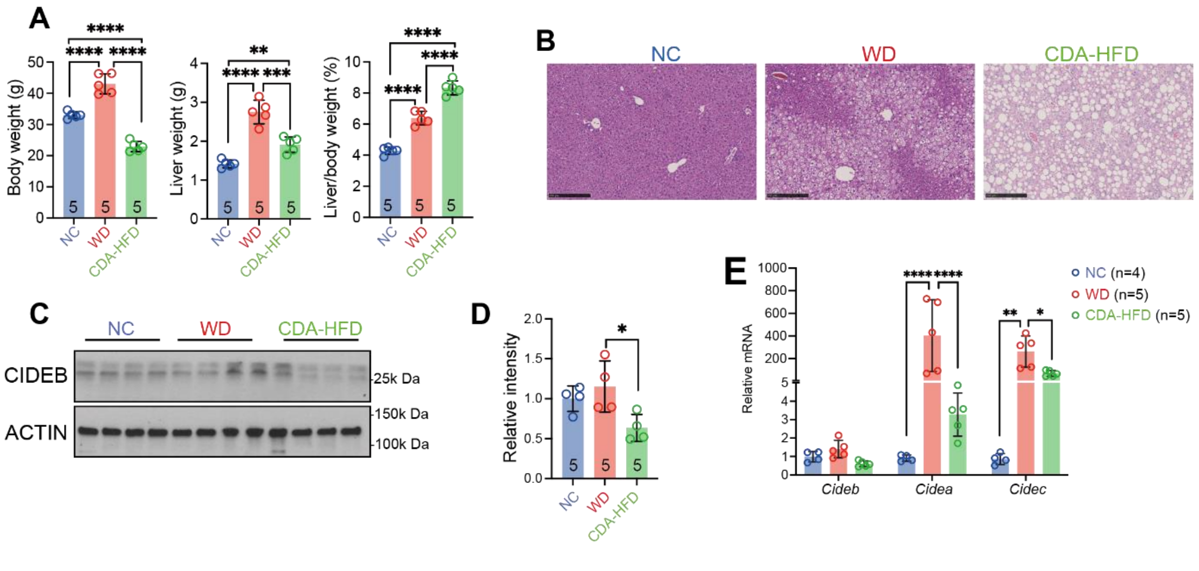

A
Body weight (g)
Liver weight (g)
Liver/body weight (%)
NC
WD
CDA-HFD
B
NC
WD
CDA-HFD
C
NC
WD
CDA-HFD
CIDEB
ACTIN
25k Da
150k Da
100k Da
D
Relative intensity
E
Relative mRNA
NC (n=4)
WD (n=5)
CDA-HFD (n=5)
Cideb
Cidea
Cidec

Figure S4

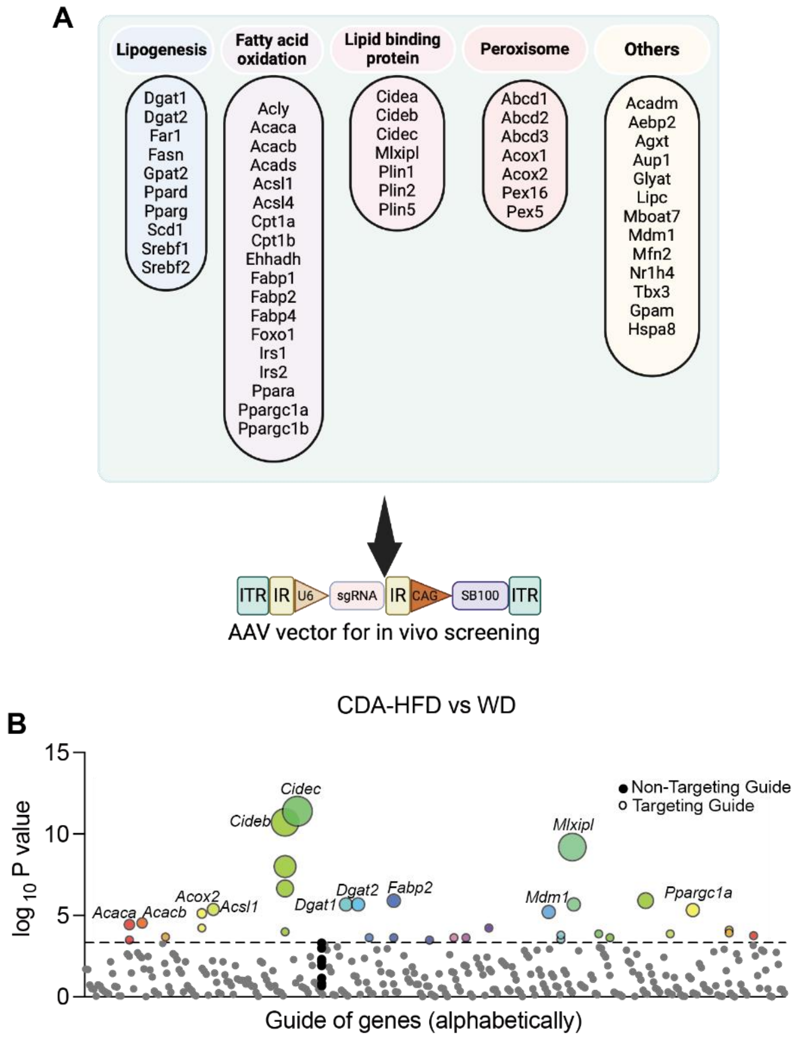
A
Lipogenesis
Dgat1
Dgat2
Far1
Fasn
Gpat2
Ppard
Pparg
Scd1
Srebf1
Srebf2
Fatty acid oxidation
Acly
Acaca
Acacb
Acads
Acsl1
Acsl4
Cpt1a
Cpt1b
Ehhadh
Fabp1
Fabp2
Fabp4
Foxo1
Irs1
Irs2
Ppara
Ppargc1a
Ppargc1b
Lipid binding protein
Cidea
Cideb
Cidec
Mlxipl
Plin1
Plin2
Plin5
Peroxisome
Abcd1
Abcd2
Abcd3
Acox1
Acox2
Pex16
Pex5
Others
Acadm
Aebp2
Agxt
Aup1
Glyat
Lipc
Mboat7
Mdm1
Mfn2
Nr1h4
Tbx3
Gpam
Hspa8
ITR
IR
U6
sgRNA
IR
CAG
SB100
ITR
AAV vector for in vivo screening
B
CDA-HFD vs WD
log 10 P value
15
10
5
0
Non-Targeting Guide
Targeting Guide
Cidec
Cideb
Mlxipl
Acox2
Dgat2
Fabp2
Mdm1
Ppargc1a
Acaca
Acacb
Acsl1
Dgat1
Guide of genes (alphabetically)

Figure S5

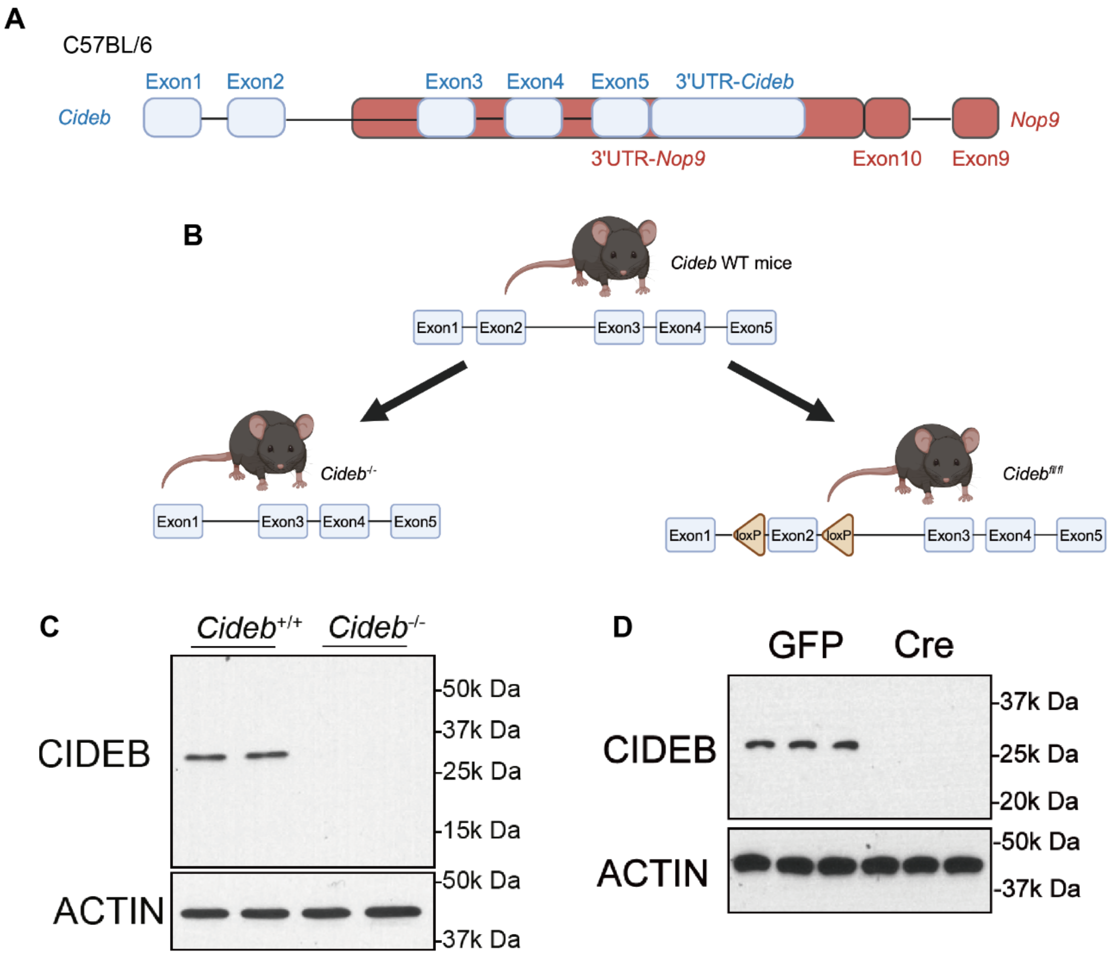

A
C57BL/6
Exon1
Exon2
Exon3
Exon4
Exon5
3'UTR-Cideb
Cideb
Nop9
3'UTR-Nop9
Exon10
Exon9
B
Cideb WT mice
Exon1
Exon2
Exon3
Exon4
Exon5
Cideb-/-
Exon1
Exon3
Exon4
Exon5
Cidebfl/fl
Exon1
loxP
Exon2
loxP
Exon3
Exon4
Exon5
C
Cideb+/+
Cideb-/-
CIDEB
ACTIN
50k Da
37k Da
25k Da
15k Da
50k Da
37k Da
D
GFP
Cre
CIDEB
ACTIN
37k Da
25k Da
20k Da
50k Da
37k Da

Figure S6

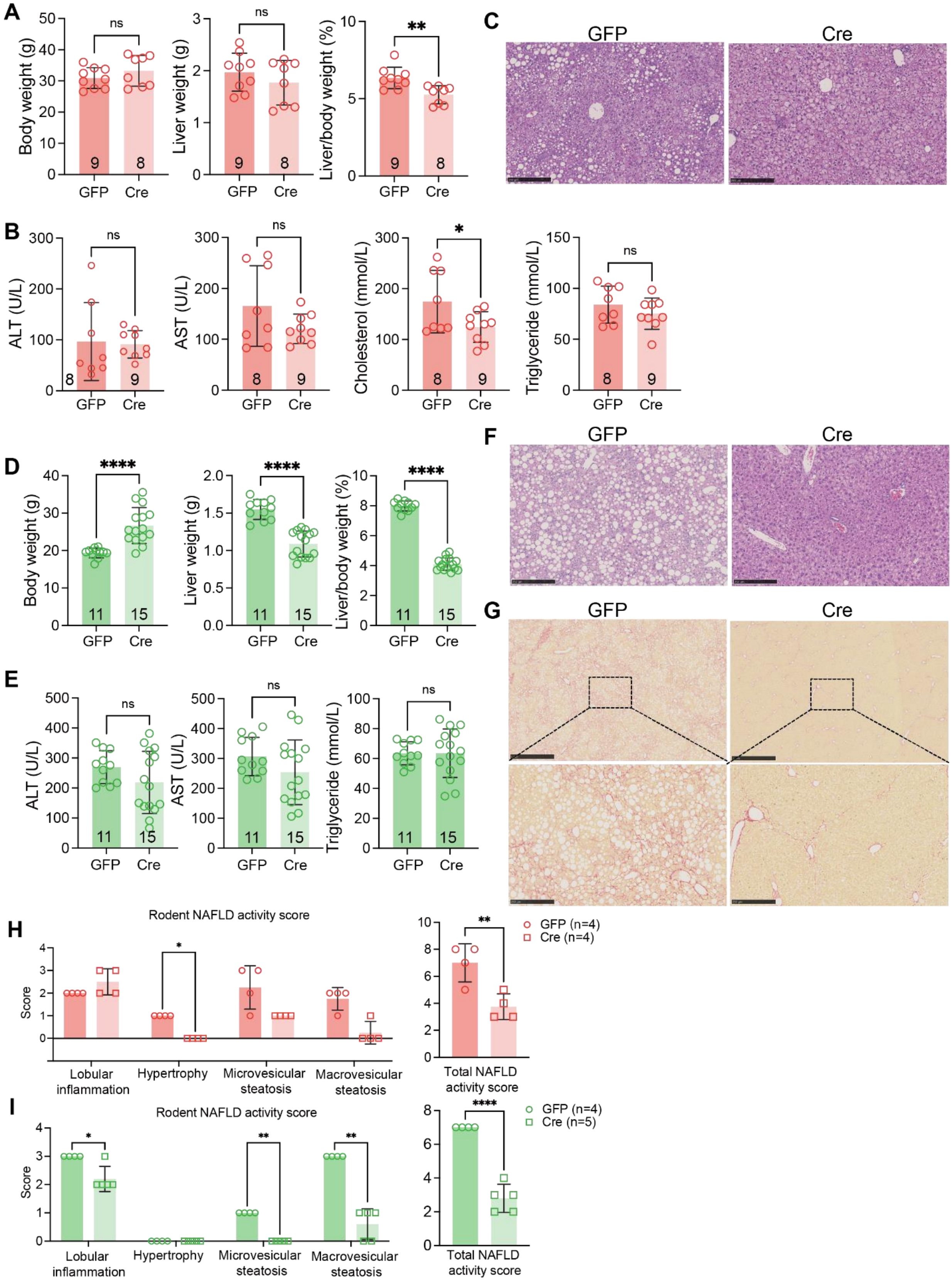
A
Body weight (g)
Liver weight (g)
Liver/body weight (%)
GFP
Cre
ns
**
B
ALT (U/L)
AST (U/L)
Cholesterol (mmol/L)
Triglyceride (mmol/L)
*
C
D
****
E
F
G
H
Rodent NAFLD activity score
Score
Lobular inflammation
Hypertrophy
Microvesicular steatosis
Macrovesicular steatosis
Total NAFLD activity score
GFP (n=4)
Cre (n=4)
I
Cre (n=5)

Figure S7

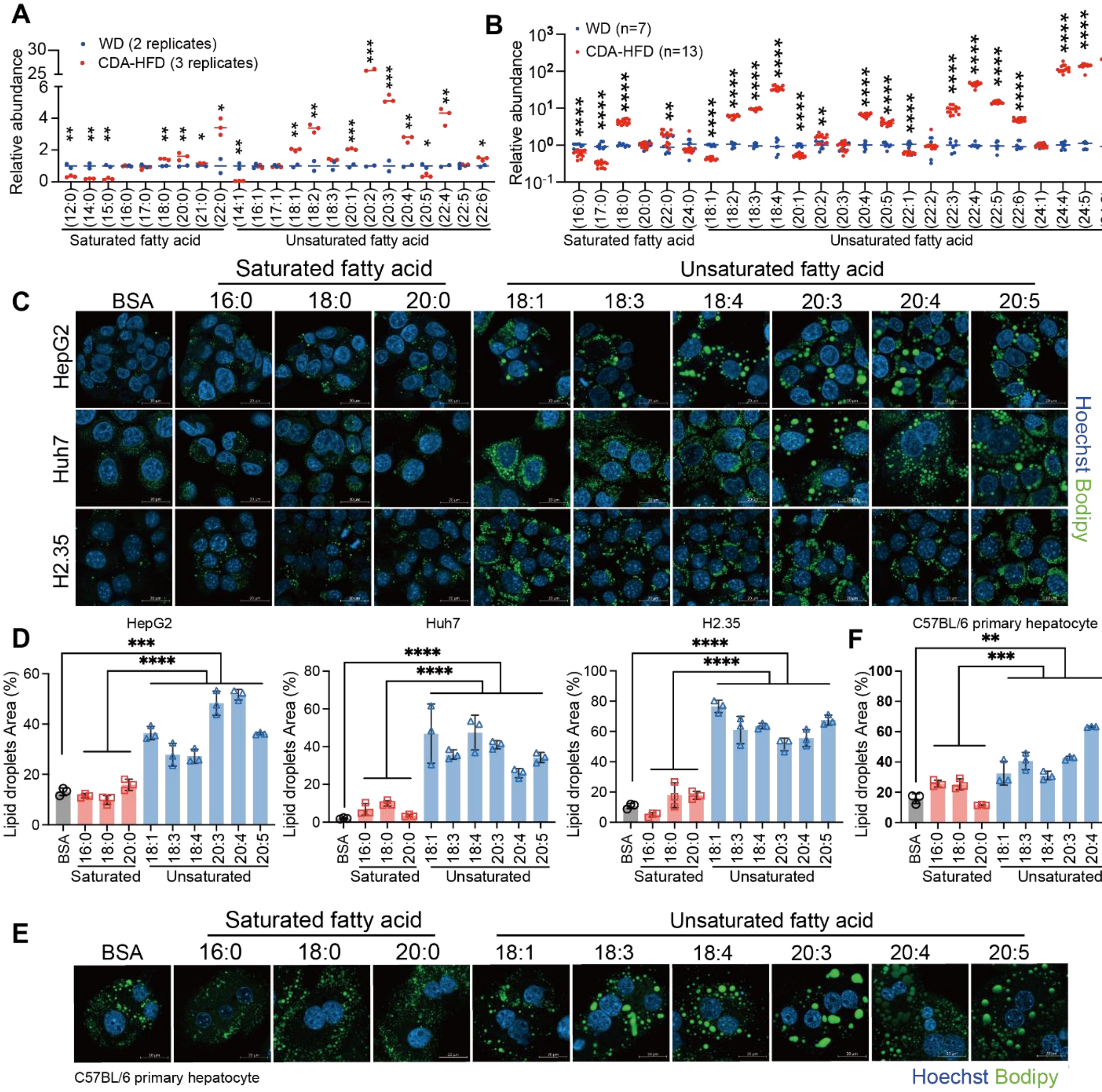
A
WD (2 replicates)
CDA-HFD (3 replicates)
Relative abundance
Saturated fatty acid
Unsaturated fatty acid
B
WD (n=7)
CDA-HFD (n=13)
Relative abundance
Saturated fatty acid
Unsaturated fatty acid
C
Saturated fatty acid
Unsaturated fatty acid
BSA
16:0
18:0
20:0
18:1
18:3
18:4
20:3
20:4
20:5
HepG2
Huh7
H2.35
Hoechst Bodipy
D
HepG2
Huh7
H2.35
Lipid droplets Area (%)
Saturated
Unsaturated
F
C57BL/6 primary hepatocyte
Lipid droplets Area (%)
Saturated
Unsaturated
E
Saturated fatty acid
Unsaturated fatty acid
BSA
16:0
18:0
20:0
18:1
18:3
18:4
20:3
20:4
20:5
C57BL/6 primary hepatocyte
Hoechst Bodipy

Figure S8

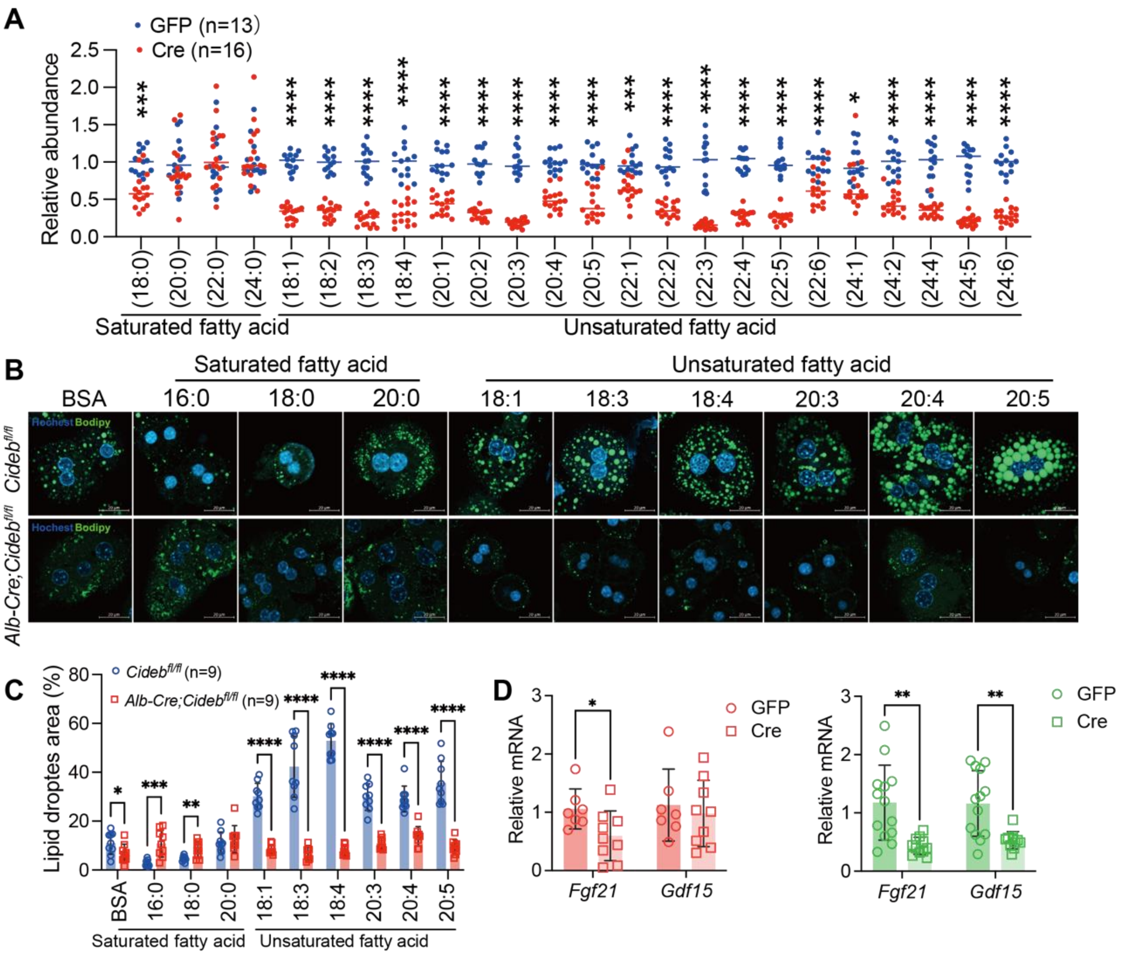

A
GFP (n=13)
Cre (n=16)
Relative abundance
(18:0) (20:0) (22:0) (24:0) (18:1) (18:2) (18:3) (18:4) (20:1) (20:2) (20:3) (20:4) (20:5) (22:1) (22:2) (22:3) (22:4) (22:5) (22:6) (24:1) (24:2) (24:4) (24:5) (24:6)
Saturated fatty acid
Unsaturated fatty acid
B
Saturated fatty acid
Unsaturated fatty acid
BSA 16:0 18:0 20:0 18:1 18:3 18:4 20:3 20:4 20:5
Cideb^fl/fl
Alb-Cre;Cideb^fl/fl
Hochest Bodipy
C
Cideb^fl/fl (n=9)
Alb-Cre;Cideb^fl/fl (n=9)
Lipid droptes area (%)
BSA 16:0 18:0 20:0 18:1 18:3 18:4 20:3 20:4 20:5
Saturated fatty acid
Unsaturated fatty acid
D
Relative mRNA
GFP
Cre
Fgf21 Gdf15

Figure S9

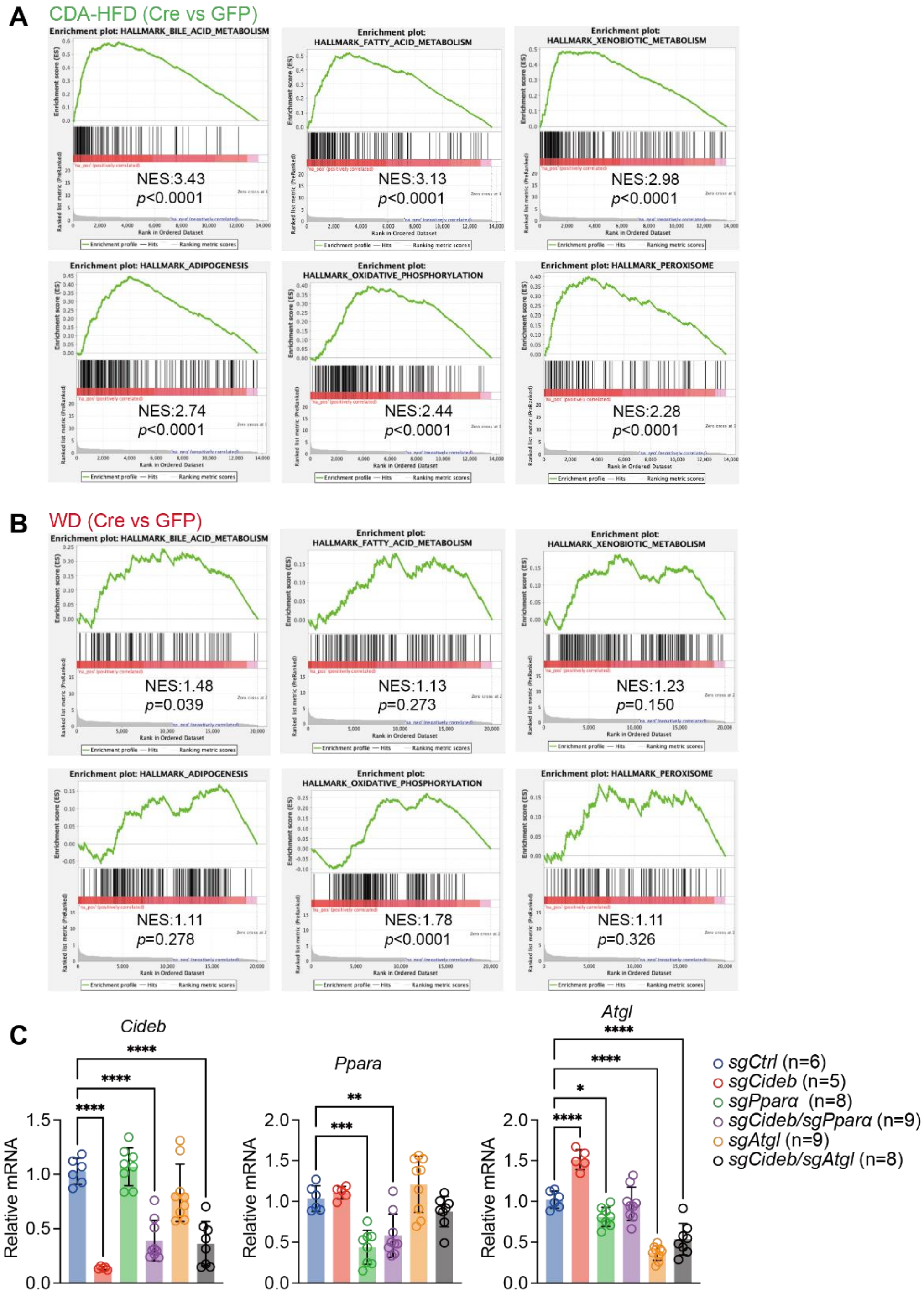
A
CDA-HFD (Cre vs GFP)
Enrichment plot: HALLMARK_BILE_ACID_METABOLISM
NES:3.43
p<0.0001
Enrichment plot: HALLMARK_FATTY_ACID_METABOLISM
NES:3.13
p<0.0001
Enrichment plot: HALLMARK_XENOBIOTIC_METABOLISM
NES:2.98
p<0.0001
Enrichment plot: HALLMARK_ADIPOGENESIS
NES:2.74
p<0.0001
Enrichment plot: HALLMARK_OXIDATIVE_PHOSPHORYLATION
NES:2.44
p<0.0001
Enrichment plot: HALLMARK_PEROXISOME
NES:2.28
p<0.0001
B
WD (Cre vs GFP)
Enrichment plot: HALLMARK_BILE_ACID_METABOLISM
NES:1.48
p=0.039
Enrichment plot: HALLMARK_FATTY_ACID_METABOLISM
NES:1.13
p=0.273
Enrichment plot: HALLMARK_XENOBIOTIC_METABOLISM
NES:1.23
p=0.150
Enrichment plot: HALLMARK_ADIPOGENESIS
NES:1.11
p=0.278
Enrichment plot: HALLMARK_OXIDATIVE_PHOSPHORYLATION
NES:1.78
p<0.0001
Enrichment plot: HALLMARK_PEROXISOME
NES:1.11
p=0.326
C
Cideb
Ppara
Atgl
Relative mRNA
sgCtrl (n=6)
sgCideb (n=5)
sgPparα (n=8)
sgCideb/sgPparα (n=9)
sgAtgl (n=9)
sgCideb/sgAtgl (n=8)

Figure S10

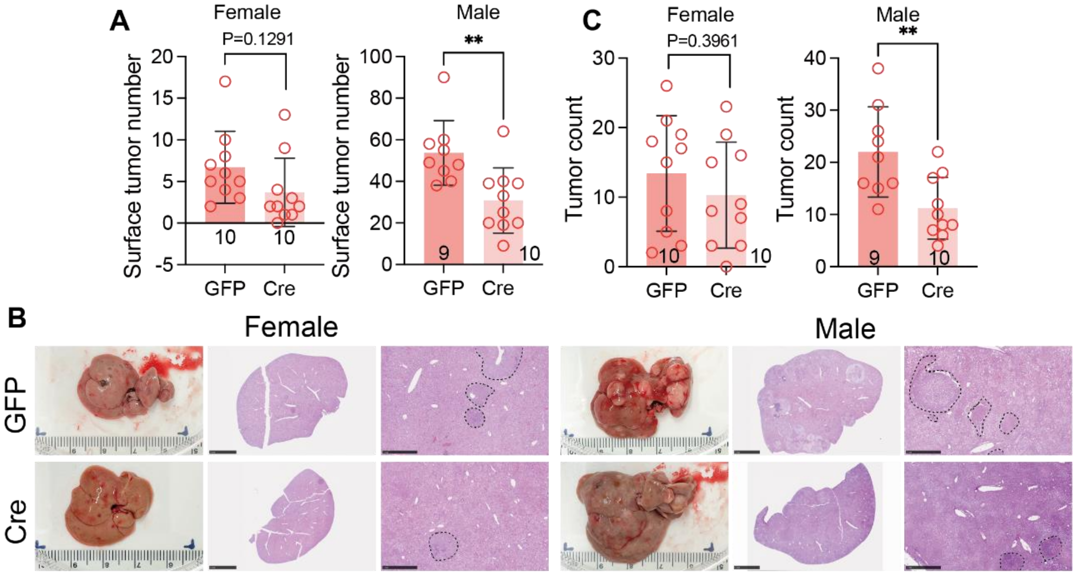
A
Female
P=0.1291
Surface tumor number
20
15
10
5
0
-5
10
10
GFP
Cre
Male
**
Surface tumor number
100
80
60
40
20
0
9
10
GFP
Cre
C
Female
P=0.3961
Tumor count
30
20
10
0
10
10
GFP
Cre
Male
**
Tumor count
40
30
20
10
0
9
10
GFP
Cre
B
Female
Male
GFP
Cre

Table S1

Qpcr primers

SPECIES	NAME	SEQUENCE	SOURCE
mouse	*Acaa1a-F*	CGCATCGCCCAATTTCTGAG	NA
mouse	*Acaa1a-R*	TTCTCCGAGGTCATCCCCAT	NA
mouse	*Acaa1b-F*	TTTCACGGCAGAAGCAGGAT	NA
mouse	*Acaa1b-R*	CATAGGACCTCAGGACCCCA	NA
mouse	*Acaca_F*	ATGGGCGGAATGGTCTCTTTC	https://pga.mgh.harvard.edu
mouse	*Acaca_R*	TGGGGACCTTGTCTTCATCAT	https://pga.mgh.harvard.edu
mouse	*Acacb_F*	CGCTCACCAACAGTAAGGTGG	https://pga.mgh.harvard.edu
mouse	*Acacb_R*	GCTTGGCAGGGAGTTCCTC	https://pga.mgh.harvard.edu
mouse	*Acadl_F*	TCTTTTCCTCGGAGCATGACA	https://pga.mgh.harvard.edu
mouse	*Acadl_R*	GACCTCTCTACTCACTTCTCCAG	https://pga.mgh.harvard.edu
mouse	*Acadm_F*	AGGGTTTAGTTTTGAGTTGACGG	https://pga.mgh.harvard.edu
mouse	*Acadm_R*	CCCCGCTTTTGTCATATTCCG	https://pga.mgh.harvard.edu
mouse	*Acads_F*	GACTGGCGACGGTTACACA	https://pga.mgh.harvard.edu
mouse	*Acads_R*	GGCAAAGTCACGGCATGTC	https://pga.mgh.harvard.edu
mouse	*Acadvl_F*	CTACTGTGCTTCAGGGACAAC	https://pga.mgh.harvard.edu
mouse	*Acadvl_R*	CAAAGGACTTCGATTCTGCCC	https://pga.mgh.harvard.edu
mouse	*Acox1-F*	TTTGTGGAACCTGTTGGCCT	NA
mouse	*Acox1-R*	AAAATCTGGGGCTCTGGCTC	NA
mouse	*Acox2-F*	AGGATTCCTACCCACCCTCC	NA
mouse	*Acox2-R*	TCGCTTGAGTGACCAGTGTC	NA
mouse	*Acsf2_F*	CTTCGGGAGGCTGTGTATCG	https://pga.mgh.harvard.edu
mouse	*Acsf2_R*	CACCATTCCAGAACTGAGAGC	https://pga.mgh.harvard.edu
mouse	*Acsl1_F*	TGCCAGAGCTGATTGACATTC	https://pga.mgh.harvard.edu
mouse	*Acsl1_R*	GGCATACCAGAAGGTGGTGAG	https://pga.mgh.harvard.edu
mouse	*Acsm1_F*	TCTGCAAAGTCTTACAGGGCT	https://pga.mgh.harvard.edu
mouse	*Acsm1_R*	TCCTCAGGTGAATCATGGTCA	https://pga.mgh.harvard.edu
mouse	*Actin B_F*	CAGAAGGAGATTACTGCTCTGGCT	NA
mouse	*Actin B_R*	TACTCCTGCTTGCTGATCCACATC	NA
mouse	*Agps-F*	CTTCTGCTCCTTGGGACAGG	NA
mouse	*Agps-R*	CCATGGTGATGTGACAGGCT	NA
mouse	*Agt-F*	CTAGGTGAGAGAGCCACCCT	NA
mouse	*Agt-R*	GAGTCCTGCTCGTAGATGGC	NA
mouse	*Cat-F*	ACCAAGGTTTGGCCTCACAA	NA
mouse	*Cat-R*	TCACACAGGCGTTTCCTCTC	NA
mouse	*Cpt1a_F*	CTCCGCCTGAGCCATGAAG	https://pga.mgh.harvard.edu
mouse	*Cpt1a_R*	CACCAGTGATGATGCCATTCT	https://pga.mgh.harvard.edu
mouse	*Cpt1b_F*	GCACACCAGGCAGTAGCTTT	https://pga.mgh.harvard.edu

mouse	*Cpt1b_R*	CAGGAGTTGATTCCAGACAGGTA	https://pga.mgh.harvard.edu
mouse	*Cpt2_F*	CAGCACAGCATCGTACCCA	https://pga.mgh.harvard.edu
mouse	*Cpt2_R*	TCCCAATGCCGTTCTCAAAAT	https://pga.mgh.harvard.edu
mouse	*Ehhadh-F*	CGGAGACTTGTGTCCGTTCA	NA
mouse	*Ehhadh-R*	CAAGCCAAGAACACCAACCG	NA
mouse	*Far1-F*	TGTAGTGGTCAACACGAGCC	NA
mouse	*Far1-R*	GGGCCTTATGGCTTACAGCA	NA
mouse	*Fasn_F*	GGAGGTGGTGATAGCCGGTAT	https://pga.mgh.harvard.edu
mouse	*Fasn_R*	TGGGTAATCCATAGAGCCCAG	https://pga.mgh.harvard.edu
mouse	*Pex11a-F*	ACAAAGAGGCCGTGGTACTG	NA
mouse	*Pex11a-R*	TCTGCTCAGTTGCCTGGATG	NA
mouse	*Pex14-F*	ATCTGCCAGGAGGGGATCTT	NA
mouse	*Pex14-R*	AAGATCCCCTCCTGGCAGAT	NA
mouse	*Pex16-F*	ACGAGCTGTCTGAACTGGTG	NA
mouse	*Pex16-R*	GGGGTGATGTCTGAATGCCA	NA
mouse	*Pex5-F*	CAGAGACCGTTTCCAAGCCT	NA
mouse	*Pex5-R*	CAAAGTCACTGGCTGTGTGC	NA
mouse	*Srebf1_F*	GATGTGCGAACTGGACACAG	https://pga.mgh.harvard.edu
mouse	*Srebf1_R*	CATAGGGGGCGTCAAACAG	https://pga.mgh.harvard.edu
mouse	*Srebf2_F*	GCAGCAACGGGACCATTCT	https://pga.mgh.harvard.edu
mouse	*Srebf2_R*	CCCCATGACTAAGTCCTTCAACT	https://pga.mgh.harvard.edu

sgRNA sequences

NAME	SEQUENCE
sgMlxipl-F1	caccgGCAAGCTGGTCTCTCCCAAG
sgMlxipl-R1	aaacCTTGGGAGAGACCAGCTTGCc
sgAcaca-F1	caccgGCAGAATTTGTTACTCGTTT
sgAcaca-R1	aaacAAACGAGTAACAAATTCTGCc
sgSrebf1-F1	caccgCTGTTTGACGCCCCCTATGC
sgSrebf1-R1	aaacGCATAGGGGGCGTCAAACAGc
sgMfn2-F1	caccgACAGGTACCCTTTGACGTCC
sgMfn2-R1	aaacGGACGTCAAAGGGTACCTGTc
sgPex5-F1	caccgAAGATTATAATGAGACCGAC
sgPex5-R1	aaacGTCGGTCTCATTATAATCTTc
sgCideb-F1	caccGAGCTCGGAGTTAAGCCGTA
sgCideb-R1	aaacACGGCTTAACTCCGAGCTC
sgPpara-F1	caccgCATCGAGTGTCGAATATGTG
sgPpara-R1	aaacCACATATTCGACACTCGATGc
sgAtgl-F1	caccgCGCCAATGTGGTAGACCCCG
sgAtgl-R1	aaacCGGGGTCTACCACATTGGCGc

Screening library sgRNAs

gene	sequence

Abcd1	CTTGACCTACCCTGATGTTG
Abcd1	CAGGGTGTACGAGATGTTCC
Abcd1	GAGGGGCCCCTGAAGATCCA
Abcd1	TCTCTCTACAGGCCAAGTTG
Abcd1	TCATCCTGCTTGAGCGCCTA
Abcd1	GTTCCAGCATGACATACCAT
Abcd2	CATGATGTACCATAAACGTT
Abcd2	TGTCGCTGAGGGGTAGTTCC
Abcd2	GTCTTCGATGAAGTGAAGAG
Abcd2	AGCAGGCTATGGTTAGCGAT
Abcd2	CAGCTGTGGACTAATTATGG
Abcd2	TCGCTAACCATAGCCTGCTT
Abcd3	TTCCTTTGCAGTAAGAAAAG
Abcd3	CTCACAGATCCTAAAAATTA
Abcd3	CTCGAACATACTGTGATGTT
Abcd3	AGAATGGGACGCTCATTGAG
Abcd3	GTGGTTTGCAGTGGCATCAT
Abcd3	ACCGCCATTACAGAATAATG
Acaca	ACATTTCATAAGACCACCGA
Acaca	ATTTGCGTAATTGTTGTTGT
Acaca	AATGCATGCGATCTATCCGT
Acaca	GCAGAATTTGTTACTCGTTT
Acaca	CAACAACAATTACGCAAATG
Acaca	AAACGAGTAACAAATTCTGC
Acaca	ACATTTCATAAGACCACCGA
Acaca	ATTTGCGTAATTGTTGTTGT
Acaca	AATGCATGCGATCTATCCGT
Acaca	GCAGAATTTGTTACTCGTTT
Acaca	CAACAACAATTACGCAAATG
Acaca	AAACGAGTAACAAATTCTGC
Acacb	GTATGCGCTCCATCCGCCGC
Acacb	CACCTGTACAGGGATTCTCT
Acacb	CGACGGTGAAATCTCTGTGC
Acacb	TCCAAACCGAGTGACAAACT
Acacb	GATCAGTACGTCCCTGTCCC
Acacb	CGTGGTCATGGTGACGCCCG
Acadm	AACAAGCATCGAACGTTCCC
Acadm	TGCCCGCAAGTTTGCCAGAG
Acadm	GTTGATCAAGCCGAGTTCCC

Acadm	CGCTGTAGGTGGCCTTGGCC
Acadm	TCGAACACAACACTCGAAAG
Acadm	CTTGATCAACGCGCACATTC
Acads	AGGCACGGCTGATCTCCTCC
Acads	GGTCCCCATTGCGGCCCAGC
Acads	GACGCTCATGATAACTCCCG
Acads	ATGCCGTGACTTTGCCGAGA
Acads	TGTGCTGGCAAATACCACCG
Acads	GTCCCGTCAGTCTCTCTACT
Acly	ACGTGTGCATCTATGCTACC
Acly	CCTTAGGTCGGCAAGGCCAA
Acly	GAGAGAGATTGACCCCGACG
Acly	TTCCTCGACGTTTGATTAAC
Acly	TCACCCGGGCGTACTTGAAC
Acly	AGAACCGGTTCAAGTACGCC
Acox1	CACTCGAAGCCAGCGTTACG
Acox1	CTCACGGATAGGGACAACAA
Acox1	CTTCGAAGCTCTGTGCACCG
Acox1	TAACAGCCACCTCGTAACGC
Acox1	ATCACTCGAGGGGAGTGCTA
Acox1	TGAGCCTTTGGACCTTCACT
Acox2	TTAAGTTGTTATATCCAGCA
Acox2	GACCCGAGATGAGCTATATG
Acox2	GTGGTCCTCTAGGCTCCGGA
Acox2	ATCCCAAGGGACATACCTGC
Acox2	CTGAACGCATTTATGCTGAC
Acox2	AACCTATGATGCAACCACCC
Acsl1	TGATCTTCGAATACCATCAG
Acsl1	CCGATGAACTGCTCGGAGCA
Acsl1	GTCCCGCTCTATGACACCCT
Acsl1	CCTTGCTCCGAGCAGTTCAT
Acsl1	GGCTTCCGAGAACCTAAACA
Acsl1	CAGTGGGTGATCGTCGAGCA
Acsl4	TCACAGCATGCAATCAGTAG
Acsl4	CAATAGAGCAGAGTACCCTG
Acsl4	CTCAACACTAGTAATCAGAT
Acsl4	GATTACTAGTGTTGAGCTTC
Acsl4	GACCATGCTGTAGCCAAATT
Acsl4	TTATCCAGAGTATCTGCTCC

Aebp2	CCGCTGTAGCCAACTCTGAC
Aebp2	GTTCTGCTGACTGAAGTGTG
Aebp2	ATTCGCTCCATACATGTCGA
Aebp2	TGTGCATCGAAGAAATCGTG
Aebp2	ACGCTGACCATCGACATGTA
Aebp2	AACAGGTGTTCGTTTGCTTG
Agxt	CAGGTACCAGTGCCTACTCC
Agxt	GCAGAGATTGCTGATCGCAT
Agxt	GGGCCTGGCCCAACACAAAC
Agxt	CGCCAGTGGATGACTCCCCG
Agxt	CCTATCTACATGGACCAACA
Agxt	CCGGTTAGAAAGGAGTCCCC
Aup1	GACTCCGCGATCACCGCGTC
Aup1	ACCACGTCCTGAATAGAAAA
Aup1	GAGGCCACCAATGGCCGAGA
Aup1	TGCCCGACAGCGTCCTCCGC
Aup1	CGCGCTACTGCTCTACGCGC
Aup1	GACGCGGTGATCGCGGAGTC
Cidea	TGTATGTGCCCGCATAGACC
Cidea	GCGAGCTGGATGTATGAGGG
Cidea	GAAATGCGTGTTGTCCCTTA
Cidea	CAAACCATGACCGAAGTAGC
Cidea	CGAGTACATCTCGTACATCG
Cidea	CATCCGATGCACAAGCTTCA
Cideb	GAGCTCGGAGTTAAGCCGTA
Cideb	TCACGTTGAGGCTGCCAAAG
Cideb	GGAGGACGACACGTGCTTGA
Cideb	CGGAGTTAAGCCGTAGGGTC
Cideb	GCAGCGAGGAAGTCCAACGG
Cideb	TTGGCCCTAAAAGAGTACTC
Cidec	TTTGCGAACCTTCCGATCTG
Cidec	GCCAACTAAGAAGATCGATG
Cidec	GGAGGAAGATGGCACAATCG
Cidec	CACTCACTTCACGATGCGCT
Cidec	AAGGCCGTCACCTGTTCTGA
Cidec	GTCGTGTTAGCACCGCAGAT
Cpt1a	CACTCACGATGTTCTTCGTC
Cpt1a	GGGGTCCACTTTGGTATGCA
Cpt1a	GCAGGCGCGGCAGAGACGTC

Cpt1a	GACTATGCGCTACTCGCTGA
Cpt1a	GGGTTACTCACGTGGTGTCT
Cpt1a	CTGTCAAAGATACCGTGAGC
Cpt1b	TCATCTTCTCCACCGGAGTC
Cpt1b	GCGTGCCAGCCACAATTCAC
Cpt1b	AGGTCCCTTACCTTTCCGGG
Cpt1b	GATGCAATCGACCAGTCCCA
Cpt1b	GCATCTCGAACATCCACCCG
Cpt1b	TGATGAAGCATATTACCGCA
Dgat1	ATTATCGTGGTATCCTGAAT
Dgat1	CACTCTCAGGTATGGCATCC
Dgat1	ATTTCAGATTGAGAAGCGCC
Dgat1	CCTGCCCCATGCGTGATTAT
Dgat1	GCATCACCACACACCAATTC
Dgat1	AAAATAACCTTGCATTACTC
Dgat2	CTTCGCGAGTACCTGATGTC
Dgat2	AAGGTGGCAGGAGATCGCAG
Dgat2	CCGAGACTACTTTCCCATCC
Dgat2	AAGGCCCTATTTGGCTACGT
Dgat2	GACTGGAACACGCCCAAGAA
Dgat2	CACCTTAGATCTGTTGAGCC
Ehhadh	AGCTGCGTTCCTCTTGCACC
Ehhadh	AACCGTCTGAGCAAATTTGA
Ehhadh	CTATCGGATTGCCAATGCAA
Ehhadh	GCTCTTGACTTAATTACCTC
Ehhadh	TATGGTCCAAACTAGCTTTC
Ehhadh	GCCATCCAAGGCGTGGCTCT
Fabp1	TCACCTATTGCCTTCATGAA
Fabp1	AAGTCCGTGACTGAACTCAA
Fabp1	CCTGCAGGCAGTCGTCAAGC
Fabp1	GTGTCAGAAATCGTGCATGA
Fabp1	GGTCCGCAATGAGTTCACCC
Fabp1	GACAATGTCGCCCAATGTCA
Fabp2	TTCTGGTAATGAACTAATCC
Fabp2	GGAGCTGATTGCTGTCCGAG
Fabp2	TTCCTTCTTAAAGAATCGCT
Fabp2	GGCCAAGCGATTCTTTAAGA
Fabp2	CGTTCCGTCTGCTAGACTGT
Fabp2	GCTGAGATCATGGCATTCGA

Fabp4	TGCCCTTTCATAAACTCTTG
Fabp4	CAGAGCATCATAACCCTAGA
Fabp4	ATGAAATCACCGCAGACGAC
Fabp4	CGATGATTACATGAAAGAAG
Fabp4	GAATTCCACGCCCAGTTTGA
Fabp4	GTCTAGGAATGTGTTATGAA
Far1	GACGATCGACGGCCTTACAA
Far1	TGAACTTACCCAACCTAAAC
Far1	GTGTAGCTATCACATTTAAC
Far1	ATCTTACAGTAGCCGCACAG
Far1	CGTCGATCGTCGGCGCCAGT
Far1	ACCGCAATGAAGAGACCACT
Fasn	GGTGCCGGCATTCAGAATCG
Fasn	GCCTACCAGGCCATCCGTAG
Fasn	AACGAACACTGGCGTCTGGG
Fasn	GGCTCTATGGATTACCCAAG
Fasn	GGAATGCCCCGCGGCCCTTG
Fasn	TGGCATAGACCCGCCGAGCC
Foxo1	GCTCGTCGCGCCGCAACGCG
Foxo1	CGCGTTGCGGCGCGACGAGC
Foxo1	AATTCGGTCATGCCAGCGTA
Foxo1	TTGGCGGCCGCGCCGCCCGA
Foxo1	TACGCTGGCATGACCGAATT
Foxo1	CACGGGGGTCAAGCGGTTCA
Glyat	GACCAGATTATGACAAAGCG
Glyat	AACCACGCTTTGTCATAATC
Glyat	GGTTGTCCGCCCTCAAGAAC
Glyat	TAAAAGCCCTAGTTGACAAG
Glyat	TTTCTACATCAATACTTACC
Glyat	ATAAATTCTTTGTATCCAGC
Gpam	CGTCTCGCTCTTGAACAAAA
Gpam	AGAGCCTTATGAGCCGGAAG
Gpam	TCAAGAGCGAGACGTCCATA
Gpam	ATAGCAGCACCTTCCCACGA
Gpam	ATGGTCGCCACCGTCTCCCC
Gpam	AGACGGTGGCGACCATCTCC
Gpat2	TGATGCCTCCCAGAAGATCA
Gpat2	ACACTACCTCTTGACCATTG
Gpat2	CTTCCCCCAGGTTCCGGGGC

Gpat2	GGGAAAGTATCGCCCCTTTA
Gpat2	CTGGCAGCAGCGGCCCATAA
Gpat2	CTGCAACGTGATCCTTGTGA
Hspa8	TATGTATTTACCTGCACCAT
Hspa8	TTTGACAGGTCGGAGCTGAA
Hspa8	ACAGATGCCAAACGTCTGAT
Hspa8	TTAGACCGTTACCAACGCTG
Hspa8	CATACCTGAATGAGTACACC
Hspa8	TGCAGAAGCGTACCTCGGAA
Irs1	CTATTTGGCACCGAACGGGT
Irs1	ACCCACGCCCATCGGCATCG
Irs1	TTACGCTATTGACGATCCTC
Irs1	CCTGCGCAGGGGAACGCTGA
Irs1	ATGCACCACTTGGAACGTCG
Irs1	CCATCAGCGTTCCCCTGCGC
Irs2	AGCCGCACTTGCGCACGCTG
Irs2	TCGAGCGCGATCACTCGCTT
Irs2	CTTATGAACATTCGCCGCTG
Irs2	CGTTCGGCCGTCACCGGTCC
Irs2	CCTGATCGCCCTCTACACCA
Irs2	CGGCTAAGGGGCGCGCGCAC
Lipc	GTTAGTGGACTGGATCTCCC
Lipc	TGTTCAAAACACCCGTATTG
Lipc	CACTTCCTACCGACCACCCG
Lipc	GCGCACGTCTCAGGGTTCGC
Lipc	GGGACGTTCCCTCAAACATA
Lipc	CCAAGATGAAAACGATCGCC
Mboat7	AGGTGAATAAGGTGAGCCCC
Mboat7	TCAGGCAGGAGGCCCAGCGT
Mboat7	ACAGCATTGGTGAAGGGCGT
Mboat7	CGCGGCAATGCAACCGCACT
Mboat7	CAGCTATAGCTACTGTTACG
Mboat7	GCATGCGCTTCTACGTTGCC
Mdm1	AGCTCCACATCCGCGCTAAC
Mdm1	GGCCTCACCTTGAAGCGCAC
Mdm1	CGACATGCCGGTGCGCTTCA
Mdm1	AAGCCATGCGCCAGTTAGCG
Mdm1	CTCATGGGCAGGACTTAGAT
Mdm1	TGGGTGGGTACCTTCTTTAC

Mfn2	AGTTACCGTCTGCATCAGCG
Mfn2	ATGACCTCGTGCTGATGGAC
Mfn2	ACAGGTACCCTTTGACGTCC
Mfn2	CCGTGATCAATGCCATGCTC
Mfn2	CAGGACGAGCAGTTGCATGC
Mfn2	AGCCACCTTCATGTGCCGCC
Mlxipl	CATAGCTCCGTAAGTCCAGC
Mlxipl	TGTCCCGGCATAGCAACTTG
Mlxipl	TTACTGGAAGCGGCGCATCG
Mlxipl	TGATGCGCGAATACCACAAG
Mlxipl	TTCCCTCAGATGTGCAACGG
Mlxipl	GCAAGCTGGTCTCTCCCAAG
Nr1h4	TGATGGACATGTACATGCGC
Nr1h4	ACGAATCCATAATATAATCC
Nr1h4	ATTCGCGGCTTCTTTGTCAC
Nr1h4	TATTCTGCCTGCCGATGCCG
Nr1h4	TTAGCCGTTTAGATTTACAC
Nr1h4	GCCGTGTACAAGTGTAAGAA
Pex16	TGCGCATGCTCCTGTTGATC
Pex16	TGTGGGGCGAAGTAGGCCGT
Pex16	GCTTCGAAAAAAGTTGCCTG
Pex16	TGAGAGCAATGACGAGCCAA
Pex16	TTCAGACAGCTCGTGTGAAT
Pex16	GTACTCTGCCTCGAACCTGC
Pex5	GGGATACAAGAGTTGCATTC
Pex5	GAATTCATCGCCGAAGTTAC
Pex5	TGAACAGTCAAACTTTCGCC
Pex5	AAGATTATAATGAGACCGAC
Pex5	TGCCAACCCGCTGATGAAGC
Pex5	GCCTCAGTCCCTCCTGCCGA
Plin1	GTCCTCTGCCGTCCGGAAGA
Plin1	CAGTCGGCCAACCCGGGTGT
Plin1	GATCCCGGGTGTGGCACCCC
Plin1	CTACCCGCCTTCGAAGCGCC
Plin1	GCCTCTGCAGGCCAACTCAT
Plin1	CACACTGGGTGGACAGCCGA
Plin2	AGAAGCCGAGCAACTATGAA
Plin2	CTCCGCTTATGTCAGTACAA
Plin2	TCCAGCCGTTCATAGTTGCT

Plin2	CACGGTCTTCACGCCCTTCT
Plin2	CCCAAGCTCACCTCGGACGT
Plin2	TCTGAACCAGCCAACGTCCG
Plin5	GTCCGGTGATCAGACAGCTC
Plin5	CAATTTGTCTAGTCCCCTGC
Plin5	CAAGGACAGGCACCCGCTGC
Plin5	CACCATGCCTGTGACACTTT
Plin5	GCAGAATGTGGTGAATCGAG
Plin5	TCAGCTCCCCACTCCAACGC
Ppara	TCTTTCCCGCGAGTATGACC
Ppara	CTCTTTGTCTTCGACGCCGT
Ppara	ATATTCGACACTCGATGTTC
Ppara	GCCGGGGGACTCGTCCGTGC
Ppara	ATGCTGATCGCGTACGGCAA
Ppara	CAAGGCCCGGGTCATACTCG
Ppard	TCGAGTATGAGAAGTGCGAT
Ppard	CAGCCCGTCTTTGTTGACGA
Ppard	CCTCGTGCACGCCATACTTG
Ppard	CCTTGCACCCCTCGCACGCG
Ppard	CCTCCGGCATCCGTCCAAAG
Ppard	CTACGGGGTCCACGCGTGCG
Pparg	AGAACCTTCTAACTCCCTCA
Pparg	CACAGAGCTGATTCCGAAGT
Pparg	TGTCAAGATCGCCCTCGCCT
Pparg	TATTTGTAATCAGCAACCAT
Pparg	CCATTGAGTGCCGAGTCTGT
Pparg	TGGCATCCGCCCAAACCTGA
Ppargc1a	CCTTGAGCATGTTGCGACTG
Ppargc1a	AGCCGTGACCACTGACAACG
Ppargc1a	GTGAGGAGGGTCATCGTTTG
Ppargc1a	GCAATCCGTCTTCATCCACG
Ppargc1a	CTTACCTCAAATATGTTCGC
Ppargc1a	GGTTGGATATGATTTCCGAT
Ppargc1b	TGACATCCCCGAAGACGATG
Ppargc1b	AGCTCGTCCACGTCAGACGC
Ppargc1b	GACAGGGCAGCACCGAAGTG
Ppargc1b	CAAAGTCACTGGCGTCCAGC
Ppargc1b	TGTTAGCAAGCCCTACCGCC
Ppargc1b	TGCGGAAATACCTTGACACA

Scd1	GGCTGTGATGCCCAGAGCGC
Scd1	CTACTACATGACCAGCGCTC
Scd1	GTGACTTACCCACGGCACCC
Scd1	GCGCTGCTCACCGAAGAGGC
Scd1	TATTCACGACCCCACCTATC
Scd1	CACCTTCTTGCGATACACTC
Srebf1	CTGTTTGACGCCCCCTATGC
Srebf1	GGTGACGGCTGTGGCGCTGC
Srebf1	CCCATCCACCGACTCGCAGC
Srebf1	GGGGCCAGCGGCAGGCTAGA
Srebf1	ATGCCATTGAGAAGCGCTAC
Srebf1	GCCACCATCTAGCCTGCCGC
Srebf2	AAGCCTCGTGACGTCCCCGC
Srebf2	CGACCCTTACTGGCACTTGA
Srebf2	GCTGCGTTCTGGTATATCAA
Srebf2	TCAGCACCGCTCCGCAGACG
Srebf2	ACAGACGGTCGCTGCGCCAC
Srebf2	CGACAAAATCATAGAGTTGA
Tbx3	CTGTACCCCGCTTGTGAAAC
Tbx3	GCCCCGCTACGGGGGAGCAA
Tbx3	CATCCATTGCTCCCCCGTAG
Tbx3	GCCGGACTTCGCCATGAGCG
Tbx3	TATCGTTGGCTCTGACGATG
Tbx3	ACGGCGATGAATTCTGTTTC
control	CGTTATTGTACACCACGCTC
control	CTCGGATGGTGTGTTGAACC
control	GGCACCGCGTTTATTGCACT
control	AGACTCGTGTGCCACGAGGA
control	CTACGGATAGGCGCGGGTGA
control	TCCGTAGGACGTATATATTC
control	AGTAGACGCTATGTTCGCGC

Table S2——Screening results

WD_vs_NC

	id	LFC	pval
1	Mfn2	-0.0364823	0.012686
2	Irs2	-0.0245933	0.013485
3	Mboat7	-0.0163528	0.027245
4	Cidec	-0.0373151	0.029304
5	Scd1	-0.0341177	0.057624
6	Aup1	-0.0060862	0.068396
7	Dgat1	-0.0009109	0.077349
8	Srebf1	-0.0099416	0.087332
9	Hspa8	0.00526467	0.25487
10	Srebf2	-0.0058488	0.1255
11	Ppard	-0.018407	0.14047
12	Irs1	-0.0206133	0.14965
13	Cideb	0.00145161	0.26245
14	Acacb	0.00409068	0.49936
15	Ppargc1b	-0.021207	0.24107
16	Plin2	-0.0037184	0.26895
17	Acadm	-0.0113314	0.30828
18	Gpam	0.00088442	0.74224
19	Fabp2	0.03096123	0.14506
20	Mdm1	-0.0089847	0.37803
21	Ehhadh	0.02162928	0.12147
22	Foxo1	-0.0099853	0.41435
23	control	-0.0046374	0.44361
24	Pparg	0.01667609	0.30161
25	Acsl1	0.02230291	0.20105
26	Acly	0.00149876	0.3204
27	Nr1h4	0.02790184	0.030163
28	Abcd2	0.00316501	0.45693
29	Far1	0.00862466	0.47983
30	Pex5	0.0288342	0.073662
31	Cpt1a	0.0066446	0.41631
32	Pex16	0.00094604	0.61856
33	Abcd1	0.00878794	0.42966
34	Fabp4	0.02419769	0.35502

35	Plin5	-0.0023282	0.67602
36	Gpat2	0.0204823	0.30435
37	Ppara	-0.003621	0.70573
38	Tbx3	0.00580994	0.52815
39	Cpt1b	0.00245466	0.57335
40	Cidea	-0.008073	0.77443
41	Acox1	-0.0141486	0.78478
42	Aebp2	-0.0011844	0.79474
43	Fabp1	0.00655261	0.71599
44	Acox2	0.02727642	0.077699
45	Dgat2	0.00219538	0.89138
46	Acsl4	0.02671709	0.085603
47	Fasn	0.01985146	0.2284
48	Plin1	0.00333288	0.78163
49	Lipc	0.0071154	0.68529
50	Agxt	0.00798508	0.63247
51	Mlxipl	0.00723022	0.51557
52	Ppargc1a	0.02409392	0.27972
53	Acads	0.00757823	0.62333
54	Abcd3	0.07185657	0.0049514
55	Acaca	0.05439604	0.00042469
56	Glyat	0.0600244	0.066747

CDA-HFD_vs_NC

	id	LFC	pval
1	Mlxipl	-0.034036	0.010717
2	Cpt1a	-0.019548	0.0211
3	Hspa8	-0.0259245	0.029014
4	Cidec	-0.07319	0.030993
5	Lipc	-0.025644	0.03429
6	Ppargc1b	-0.0371248	0.051218
7	Acadm	-0.0247751	0.067137
8	Fasn	0.01943498	0.18004
9	Mboat7	0.00370345	0.28195
10	Ehhadh	-0.0168134	0.20341
11	Acsl1	0.01602121	0.12514
12	Mdm1	-0.0159636	0.23054
13	Acads	-0.0001785	0.25607
14	Dgat1	-0.0158696	0.27568

15	Acaca	-0.0109371	0.2826
16	Acox1	-0.0192531	0.2963
17	Irs2	-0.0015362	0.30466
18	Pex16	-0.0255702	0.31079
19	Cpt1b	-0.0209138	0.32871
20	Pex5	-0.0218603	0.34572
21	Aebp2	-0.0023366	0.35868
22	Acly	0.00524434	0.093648
23	Nr1h4	0.00482545	0.23938
24	Pparg	-0.0009988	0.37183
25	Fabp4	-0.010389	0.40344
26	Ppargc1a	-0.0141376	0.41699
27	Abcd1	-0.0108029	0.42342
28	Abcd2	0.00200514	0.69564
29	Srebf1	0.02455909	0.15279
30	Cideb	0.08245317	0.0011242
31	Fabp1	0.00727905	0.45607
32	Ppard	-0.0036512	0.54108
33	Gpam	0.0212804	0.057354
34	Glyat	0.03772105	0.044023
35	Foxo1	-0.0030471	0.60654
36	Acsl4	-0.0110868	0.61686
37	Irs1	-0.0068242	0.68356
38	Far1	0.00945585	0.46984
39	Plin1	0.0124404	0.53426
40	Cidea	-0.0053216	0.74025
41	Mfn2	0.00738768	0.37637
42	Srebf2	0.00813254	0.35349
43	Agxt	-0.0001925	0.78882
44	Ppara	0.00370939	0.54149
45	Acox2	0.03697413	0.086163
46	Aup1	0.00491858	0.31318
47	control	0.02527958	0.10437
48	Fabp2	0.00685234	0.51286
49	Acacb	0.01649558	0.27726
50	Scd1	-0.0033942	0.87974
51	Tbx3	0.02486575	0.30565
52	Abcd3	0.03503124	0.23275
53	Plin5	0.03367571	0.074451

54	Plin2	0.01156122	0.61562
55	Gpat2	0.02210225	0.31168
56	Dgat2	0.04306275	0.087662

CDA-HFD_vs_WD

	id	LFC	pval
1	Acaca	-0.0636789	0.002713
2	Mlxipl	-0.0391195	0.0065303
3	Cidec	-0.0384128	0.031083
4	Pex5	-0.0470971	0.036239
5	Nr1h4	-0.0195125	0.07591
6	Hspa8	-0.0284097	0.087342
7	Ehhadh	-0.0365434	0.12006
8	Fabp2	-0.0191725	0.12131
9	Cpt1a	-0.025503	0.12692
10	Ppargc1a	-0.0358928	0.14941
11	Cpt1b	-0.0219891	0.1732
12	Glyat	-0.0200449	0.201
13	Acox2	0.01431054	0.22326
14	Lipc	-0.0312937	0.25663
15	Dgat1	-0.0122749	0.27597
16	Pex16	-0.0243976	0.28076
17	Fasn	0.00038297	0.7488
18	Acacb	0.01535121	0.064988
19	Acads	-0.0069964	0.37478
20	Abcd1	-0.0176751	0.38619
21	Ppargc1b	-0.0142046	0.43838
22	Acsl1	-0.0043003	0.45789
23	Acly	0.00574406	0.17144
24	Fabp4	-0.033306	0.48476
25	Abcd3	-0.0351546	0.49131
26	Mdm1	-0.0052769	0.51133
27	Srebf1	0.03614396	0.018352
28	Acsl4	-0.0364687	0.52484
29	Gpam	0.02209783	0.064319
30	Acadm	-0.0118536	0.56639
31	Cideb	0.08217974	2.50E-05
32	Foxo1	0.00881276	0.46638
33	Aebp2	0.00023952	0.60081

34	Aup1	0.01322178	0.30289
35	Acox1	-0.0037624	0.63382
36	Tbx3	0.02091116	0.18757
37	Pparg	-0.0161957	0.67029
38	Gpat2	0.00389508	0.47349
39	Srebf2	0.01597677	0.17891
40	Fabp1	0.00210884	0.48061
41	Mboat7	0.02124371	0.49336
42	Irs2	0.0245641	0.092638
43	Far1	0.00222541	0.8672
44	Ppard	0.01641627	0.36696
45	Plin1	0.01057266	0.52199
46	Agxt	-0.0066941	0.88315
47	Cidea	0.00422825	0.72395
48	Abcd2	0.0003894	0.85348
49	Ppara	0.00860445	0.79615
50	Plin2	0.01707052	0.49402
51	Irs1	0.01522875	0.31062
52	Dgat2	0.04250751	0.20223
53	control	0.03126562	0.4842
54	Scd1	0.03228382	0.34738
55	Plin5	0.03719508	0.14691
56	Mfn2	0.0450882	0.028664

Table S3——RNAseq (the differentially expressed genes)

gene	baseMean	log2FoldCha	lfcSE	stat	pvalue	padj
0610005C13Rik	964.41368	0.62484186	0.21237629	2.94214514	0.00325947	0.01992811
0610006L08Rik	0	NA	NA	NA	NA	NA
0610009B22Rik	145.503984	-0.0089569	0.13289053	-0.0674005	0.94626284	0.97121815
0610009E02Rik	5.76274067	-0.6968054	0.49352898	-1.4118835	0.15798426	0.31555837
0610009L18Rik	7.07269121	-0.974747	0.52004273	-1.8743595	0.06088089	0.16366905
0610010F05Rik	372.405849	-0.3316797	0.10506164	-3.157001	0.00159401	0.01179236
0610010K14Rik	121.780537	-0.0179017	0.16413245	-0.1090688	0.91314789	0.9533224
0610012G03Rik	222.680404	-0.0153946	0.13049059	-0.1179748	0.90608761	0.94951944
0610025J13Rik	0.72870449	0.13189874	1.632761	0.08078264	0.93561482	NA
0610030E20Rik	549.380379	0.19814829	0.09426067	2.10213109	0.03554179	0.111789
0610031O16Rik	123.473145	0.92227126	0.30885173	2.98612952	0.00282533	0.01789107
0610038B21Rik	7.43331959	-1.0093891	0.50643953	-1.9931089	0.04624952	0.13478664
0610039H22Rik	8.81167784	0.00630538	0.45158909	0.01396265	0.98885978	0.99414429
0610039K10Rik	12.9842504	-0.3316443	0.3839597	-0.8637476	0.38772655	0.57107184
0610040B10Rik	7.90163618	-0.0664294	0.42525303	-0.1562115	0.87586629	0.93042479
0610040F04Rik	0.97392046	0.93384351	1.33597143	0.69899961	0.48455228	NA
0610040J01Rik	323.496268	0.24460918	0.16880731	1.4490438	0.14732535	0.30003426
0610042G04Rik	14.3583904	-0.6524787	0.41958329	-1.5550635	0.11993095	0.26156173
0610043K17Rik	64.8181886	0.30779342	0.26665485	1.15427647	0.24838684	0.42796989
1100001I12Rik	0.09331208	0.70897419	4.27958794	0.16566412	0.86842128	NA
1110002E22Rik	8.09065251	0.28089033	0.46042343	0.61006957	0.54181572	0.7032039
1110002J07Rik	9.43952398	1.17438773	0.52797978	2.22430436	0.02612797	0.08972772
1110002L01Rik	29.9538827	0.4870916	0.23348021	2.08622219	0.0369585	0.11481874
1110002O04Rik	0.27181117	1.07245269	2.4843087	0.43169059	0.66596631	NA
1110004F10Rik	253.319761	-0.1603169	0.12463174	-1.286325	0.19832965	0.36758883
1110006O24Rik	7.90731531	0.17801728	0.4501899	0.39542709	0.69252772	0.81408735
1110008P14Rik	130.598117	-0.317699	0.12742973	-2.4931313	0.0126622	0.05311275
1110015O18Rik	0	NA	NA	NA	NA	NA
1110017D15Rik	1.48747407	1.58712467	1.00849712	1.57375231	0.11554472	NA
1110018N20Rik	26.3022088	0.46405161	0.3363082	1.37984033	0.16763581	0.32869896
1110019D14Rik	40.7194392	0.16313175	0.19702994	0.82795411	0.40769649	0.58940237
1110020A21Rik	8.92780026	0.26002091	0.42861149	0.60665875	0.5440774	0.70466659
1110025L11Rik	0	NA	NA	NA	NA	NA
1110025M09Rik	72.565375	0.13900712	0.15685954	0.88618852	0.37551595	0.56054183
1110028F11Rik	98.0111318	0.94259751	0.24261095	3.8852224	0.00010224	0.00159402
1110028F18Rik	0.07565308	0.13190146	4.27958794	0.03082106	0.97541224	NA
1110032A03Rik	241.797938	0.09873984	0.16117719	0.61261671	0.54012981	0.70195093
1110032F04Rik	0.30647079	-2.0611197	2.89395441	-0.7122157	0.47633122	NA
1110036E04Rik	0.49626817	1.60669318	1.85501364	0.86613551	0.38641586	NA
1110038B12Rik	95.3101136	-0.2334732	0.1664304	-1.4028281	0.16066811	0.31900792
1110038F14Rik	85.5632268	0.100355	0.20894106	0.48030293	0.631012	0.77217618
1110046J04Rik	4.70525293	-0.5595861	0.53720316	-1.0416657	0.2975667	0.48085664
1110051M20Rik	121.438917	0.06588134	0.10754585	0.61258844	0.5401485	0.70195093
1110057P08Rik	0	NA	NA	NA	NA	NA
1110058D11Rik	6.02817572	0.87340554	0.53120658	1.64419186	0.10013658	0.23140032
1110059E24Rik	51.0207345	-0.0828096	0.24541689	-0.3374243	0.73579708	0.84341665
1110059G10Rik	112.448649	-0.0942859	0.15011578	-0.6280878	0.52994644	0.69355895
1110065P20Rik	52.1120737	0.26334413	0.28740271	0.91628966	0.35951499	0.54449863
1190005I06Rik	0.5004179	0.70897445	1.78750768	0.39662736	0.69164227	NA
1190007I07Rik	58.4686742	0.58146718	0.22780949	2.55242735	0.01069752	0.04706251
1190028D05Rik	0	NA	NA	NA	NA	NA
1300002E11Rik	189.212896	0.09625401	0.10699918	0.89957709	0.36834535	0.55397483
1300017J02Rik	5965.55943	0.68324125	0.1580091	4.32406277	1.53E-05	0.00039357
1500002C15Rik	0	NA	NA	NA	NA	NA
1500002F19Rik	137.569897	0.22587302	0.12991299	1.73864843	0.08209662	0.20133147
1500004A13Rik	27.6575665	0.77797022	0.43259179	1.79839343	0.07211468	0.18437911
1500009C09Rik	0.03258441	-0.4451501	4.27958794	-0.1040171	0.91715581	NA
1500009L16Rik	2.86076911	-1.9251098	0.87337063	-2.2042301	0.02750817	NA
1500011B03Rik	19.081167	-0.6708049	0.37329288	-1.7969935	0.07233666	0.18468698
1500012K07Rik	0.03258441	-0.4451501	4.27958794	-0.1040171	0.91715581	NA
1500015A07Rik	14.5977942	-0.3490309	0.31264522	-1.11638	0.26425949	0.44489538
1500015L24Rik	5.00005604	2.4271521	0.80212951	3.02588554	0.00247906	0.01629144
1500026H17Rik	18.0451232	0.2924689	0.2748841	1.0639717	0.28734152	0.47008081
1500035N22Rik	35.7260295	0.51690699	0.31926144	1.61907116	0.10543195	0.23952163
1520401A03Rik	1.11806454	-0.1111812	1.30403611	-0.0852593	0.93205524	NA
1600002D24Rik	0.07598251	-1.0352329	4.27864469	-0.2419535	0.80881623	NA
1600010M07Rik	2.77910214	-1.0262694	0.65302283	-1.5715674	0.1160509	NA
1600012H06Rik	438.489828	-0.0079196	0.08660768	-0.0914422	0.9271412	0.96047438
1600012P17Rik	0	NA	NA	NA	NA	NA
1600014C10Rik	2457.49274	0.37331476	0.12950874	2.88254483	0.00394477	0.0229981
1600014C23Rik	0	NA	NA	NA	NA	NA
1600015H23Rik	0	NA	NA	NA	NA	NA
1600019K03Rik	0.04534625	0.7089689	4.27958794	0.16566289	0.86842225	NA
1600020E01Rik	63.8485572	0.36351441	0.2528784	1.43750673	0.15057406	0.30434309
1600023N17Rik	3.26914604	0.20479671	0.65593074	0.31222307	0.754871	NA
1600025M17Rik	0	NA	NA	NA	NA	NA
1600027J07Rik	0	NA	NA	NA	NA	NA
1600029I14Rik	1.27922338	-0.6574176	1.36352203	-0.4821467	0.62970177	NA
1600029O15Rik	0.0519703	0	4.27958794	0	1	NA
1700001C19Rik	53.4141057	0.58389461	0.31735451	1.83988121	0.06578568	0.17272303
1700001D01Rik	14.5232723	-0.3725999	0.31082465	-1.1987464	0.23062657	0.4069378
1700001F09Rik	0	NA	NA	NA	NA	NA
1700001G01Rik	0	NA	NA	NA	NA	NA
1700001G11Rik	0.62117401	-1.5015661	1.36811491	-1.0975439	0.27240372	NA
1700001G17Rik	1.15407265	0.20437536	1.09434659	0.1867556	0.85185226	NA
1700001J03Rik	5.11781373	0.35493254	0.48237559	0.73580122	0.46185168	0.63725211
1700001J04Rik	0	NA	NA	NA	NA	NA
1700001J11Rik	0.1786856	1.2005418	4.11274436	0.29190771	0.77035719	NA
1700001K19Rik	14.2987742	-0.1633174	0.2662132	-0.6134835	0.53955669	0.70140351
1700001K23Rik	2.11428374	-0.365394	0.69386397	-0.5266076	0.59846614	NA
1700001L05Rik	176.101811	-0.0581686	0.25645674	-0.2268165	0.8205664	0.89702225
1700001L19Rik	0.68018133	-1.1497993	1.15506146	-0.9954442	0.31952025	NA
1700001O22Rik	1.33749787	0.37752707	0.79739696	0.47344935	0.63589263	NA
1700001P01Rik	2.28697553	0.26171663	0.84848216	0.30845272	0.75773787	NA
1700003C15Rik	0	NA	NA	NA	NA	NA
1700003D09Rik	2.23041827	0.43938174	1.18873941	0.3696199	0.71166572	NA
1700003E16Rik	19.8178189	0.73217871	0.32287865	2.26765907	0.02334999	0.08255492
1700003E24Rik	0	NA	NA	NA	NA	NA
1700003F12Rik	1.58684071	-0.5093732	0.84742387	-0.6010843	0.54778386	NA
1700003G18Rik	0.2801571	-0.4385967	2.15893935	-0.2031538	0.83901484	NA
1700003H04Rik	0	NA	NA	NA	NA	NA
1700003I22Rik	0	NA	NA	NA	NA	NA
1700003L19Rik	0	NA	NA	NA	NA	NA
1700003M07Rik	0.0683904	0	4.27958794	0	1	NA
1700003P14Rik	0	NA	NA	NA	NA	NA
1700006A11Rik	0	NA	NA	NA	NA	NA
1700006F04Rik	0.0519703	0	4.27958794	0	1	NA
1700006H20Rik	0.32841089	-1.8805917	2.42425581	-0.7757398	0.43790265	NA
1700006H21Rik	0.03258441	-0.4451501	4.27958794	-0.1040171	0.91715581	NA
1700007E05Rik	0.08622771	-0.4451449	4.27958794	-0.1040158	0.91715679	NA
1700007F19Rik	14.8144091	0.80773266	0.38221739	2.11328077	0.03457674	0.10956934
1700007J10Rik	7.62115941	0.1184701	0.40823987	0.29019729	0.77166531	0.86638564
1700007K13Rik	0.60290342	-0.3004464	1.60103358	-0.1876578	0.85114491	NA
1700007L15Rik	13.1906455	-0.1381003	0.31100804	-0.4440408	0.65701307	0.78980537
1700007P06Rik	0.46188774	-0.3520869	1.6328771	-0.2156237	0.82928112	NA
1700008C04Rik	0	NA	NA	NA	NA	NA
1700008J07Rik	39.3352062	0.12449329	0.20244244	0.6149565	0.53858345	0.70059978
1700008K24Rik	0.10777615	-0.4451449	4.27958794	-0.1040158	0.91715679	NA
1700008O03Rik	2.64713814	0.40587627	0.66488463	0.61044616	0.5415663	NA
1700008P02Rik	0	NA	NA	NA	NA	NA
1700009C05Rik	0.11298677	-0.4451449	4.27958794	-0.1040158	0.91715679	NA
1700009J07Rik	1.02205543	0.57390921	0.90422227	0.63469927	0.52562457	NA
1700009N14Rik	0.09240878	-1.0352329	4.27864469	-0.2419535	0.80881623	NA
1700010B08Rik	0.15169716	0.70897947	4.27958794	0.16566536	0.86842031	NA
1700010D01Rik	0.1038867	0	4.27958794	0	1	NA
1700010G06Rik	12.5004659	0.29196522	0.3162675	0.9231591	0.3559243	0.54078271
1700010H22Rik	0.05930715	0	4.27958794	0	1	NA
1700010I02Rik	0	NA	NA	NA	NA	NA
1700010I14Rik	8.76944729	-0.2184502	0.39756745	-0.549467	0.58268501	0.73372976
1700010J16Rik	0.048469	-0.4451501	4.27958794	-0.1040171	0.91715581	NA
1700010K23Rik	0	NA	NA	NA	NA	NA
1700010L04Rik	0.96270844	1.44816048	1.27581718	1.13508464	0.25633986	NA
1700010N08Rik	0	NA	NA	NA	NA	NA
1700011B04Rik	0	NA	NA	NA	NA	NA
1700011C11Rik	0	NA	NA	NA	NA	NA
1700011L22Rik	0.67872688	-2.1240879	1.66100301	-1.2787984	0.20096807	NA
1700011M02Rik	0	NA	NA	NA	NA	NA
1700012A03Rik	0.15811312	1.15219578	4.23139865	0.27229668	0.78539391	NA
1700012B07Rik	0.13205526	1.79596894	4.26590466	0.42100541	0.67375113	NA
1700012B09Rik	0.10046196	0.70897419	4.27958794	0.16566412	0.86842128	NA
1700012C14Rik	0.1245032	-1.0134482	4.27892803	-0.2368463	0.81277604	NA
1700012D01Rik	12.7490627	0.20952429	0.33296768	0.62926315	0.52917679	0.6929941
1700012D14Rik	10.8234162	-0.1285326	0.36043156	-0.3566074	0.7213857	0.83308921
1700012D16Rik	1.66609304	-1.4721221	0.83075521	-1.7720287	0.07638979	NA
1700012H19Rik	0	NA	NA	NA	NA	NA
1700012I11Rik	0.05930715	0	4.27958794	0	1	NA
1700012J15Rik	0	NA	NA	NA	NA	NA
1700012P22Rik	0	NA	NA	NA	NA	NA
1700013D24Rik	0	NA	NA	NA	NA	NA
1700013F07Rik	0.46488483	-0.2411879	1.66185362	-0.1451319	0.88460675	NA
1700013G24Rik	0	NA	NA	NA	NA	NA
1700013H16Rik	0.15614678	0	4.22391603	0	1	NA
1700013N06Rik	0	NA	NA	NA	NA	NA
1700014D04Rik	0.40802801	0.12475589	1.90739255	0.06540651	0.94785034	NA
1700014L14Rik	0	NA	NA	NA	NA	NA
1700015C15Rik	0.19691635	0.70895941	4.03875664	0.17553903	0.86065611	NA
1700015G11Rik	0.74098039	1.32851938	1.24953048	1.06321487	0.28768452	NA
1700015I17Rik	0.16495332	0.70897888	4.16633681	0.1701684	0.8648777	NA
1700016A09Rik	2.54285703	-0.1576593	0.62211891	-0.2534231	0.79994127	NA
1700016B01Rik	0	NA	NA	NA	NA	NA
1700016C15Rik	1.78048571	-0.8178218	0.87469193	-0.9349827	0.34979723	NA
1700016C19Rik	0	NA	NA	NA	NA	NA
1700016D06Rik	0	NA	NA	NA	NA	NA
1700016G22Rik	0.1056136	0	4.27958794	0	1	NA
1700016H13Rik	0.03799125	-0.4451501	4.27958794	-0.1040171	0.91715581	NA
1700016J18Rik	0.87108007	-1.1800605	1.81331796	-0.6507741	0.51519229	NA
1700016K05Rik	1.15067941	-0.0127096	0.96451539	-0.0131771	0.98948647	NA
1700016K19Rik	0.48384527	-1.1432665	1.51690745	-0.7536824	0.45103993	NA
1700016L04Rik	0.04306867	0.7089689	4.27958794	0.16566289	0.86842225	NA
1700016L21Rik	0	NA	NA	NA	NA	NA
1700016P04Rik	0	NA	NA	NA	NA	NA
1700017B05Rik	404.01608	-0.0978028	0.18826125	-0.5195057	0.60340811	0.7497252
1700017G19Rik	0.3227136	-1.468068	2.11680721	-0.6935294	0.48797739	NA
1700017J07Rik	0	NA	NA	NA	NA	NA
1700017L05Rik	0	NA	NA	NA	NA	NA
1700017M07Rik	0	NA	NA	NA	NA	NA
1700017N19Rik	0.05352616	0	4.27958794	0	1	NA
1700018A04Rik	0	NA	NA	NA	NA	NA
1700018A23Rik	3.26943839	-0.1326319	0.63144887	-0.2100438	0.83363347	NA
1700018B08Rik	0.11102578	0.70897419	4.27958794	0.16566412	0.86842128	NA
1700018B24Rik	0	NA	NA	NA	NA	NA
1700018F24Rik	0	NA	NA	NA	NA	NA
1700018G05Rik	0.048469	-0.4451501	4.27958794	-0.1040171	0.91715581	NA
1700018L02Rik	10.875986	1.13108628	0.49120441	2.3026794	0.02129689	0.07727606
1700019A02Rik	3.6699534	0.05816472	0.53491973	0.10873541	0.91341235	0.95342803
1700019C18Rik	0	NA	NA	NA	NA	NA
1700019D03Rik	7.3581963	0.16695281	0.40836503	0.40883229	0.68266274	0.80694827
1700019E08Rik	0	NA	NA	NA	NA	NA
1700019F05Rik	0	NA	NA	NA	NA	NA
1700019G24Rik	0	NA	NA	NA	NA	NA
1700019J19Rik	0	NA	NA	NA	NA	NA
1700019M22Rik	0	NA	NA	NA	NA	NA
1700019N19Rik	0.27622359	0.13189935	2.49024312	0.05296646	0.95775863	NA
1700019P21Rik	0	NA	NA	NA	NA	NA
1700020A23Rik	0.33413943	0.12168426	2.5322396	0.04805401	0.9616732	NA
1700020D12Rik	0	NA	NA	NA	NA	NA
1700020D14Rik	0	NA	NA	NA	NA	NA
1700020G03Rik	0.14322517	0.13190674	4.27958794	0.0308223	0.97541126	NA
1700020G17Rik	0	NA	NA	NA	NA	NA
1700020L13Rik	0.18007477	0	4.07730967	0	1	NA
1700020L24Rik	2.44956088	-0.0650912	0.73419084	-0.088657	0.92935448	NA
1700020M21Rik	0	NA	NA	NA	NA	NA
1700020N01Rik	0.35420923	-2.2116925	2.94404404	-0.751243	0.45250643	NA
1700020N15Rik	0	NA	NA	NA	NA	NA
1700020N18Rik	0.11679537	0	4.27958794	0	1	NA
1700021F02Rik	0.08419564	-1.0352325	4.2786447	-0.2419534	0.8088163	NA
1700021F07Rik	0.03799125	-0.4451501	4.27958794	-0.1040171	0.91715581	NA
1700021G15Rik	0	NA	NA	NA	NA	NA
1700021L23Rik	0	NA	NA	NA	NA	NA
1700021N21Rik	0	NA	NA	NA	NA	NA
1700022A21Rik	0.04123002	0.7089689	4.27958794	0.16566289	0.86842225	NA
1700022A22Rik	0	NA	NA	NA	NA	NA
1700022E09Rik	0.05352616	0	4.27958794	0	1	NA
1700022H01Rik	3.74768313	-0.1951561	0.53200861	-0.3668288	0.7137467	0.82755432
1700022H16Rik	0	NA	NA	NA	NA	NA
1700022I11Rik	6.00435071	-0.2805119	0.4399263	-0.6376338	0.5237121	0.68887819
1700022N22Rik	8.8058463	0.34130227	0.43592406	0.78293973	0.4336625	0.61190553
1700023A20Rik	0.42538342	1.95554226	2.01154155	0.97216101	0.33097045	NA
1700023C21Rik	0	NA	NA	NA	NA	NA
1700023D09Rik	6.57589028	-0.1613732	0.43486172	-0.3710908	0.71056987	0.8256632
1700023F02Rik	0	NA	NA	NA	NA	NA
1700023G09Rik	0	NA	NA	NA	NA	NA
1700024B05Rik	0	NA	NA	NA	NA	NA
1700024B18Rik	0.14761167	0.13190674	4.27958794	0.0308223	0.97541126	NA
1700024G13Rik	0.05930715	0	4.27958794	0	1	NA
1700024I08Rik	0	NA	NA	NA	NA	NA
1700024J04Rik	0.45617712	1.20635227	1.87173088	0.6445116	0.51924374	NA
1700024N05Rik	0	NA	NA	NA	NA	NA
1700025B11Rik	0	NA	NA	NA	NA	NA
1700025C18Rik	0.03258441	-0.4451501	4.27958794	-0.1040171	0.91715581	NA
1700025D23Rik	0	NA	NA	NA	NA	NA
1700025F24Rik	0.19181131	-0.4580193	4.07476814	-0.1124038	0.91050326	NA
1700025G04Rik	136.381666	0.02988763	0.15411655	0.19392876	0.84623166	0.91359589
1700025H01Rik	0.06326921	0	4.27958794	0	1	NA
1700025J12Rik	0	NA	NA	NA	NA	NA
1700025K04Rik	0.06451777	0	4.27958794	0	1	NA
1700025K24Rik	0	NA	NA	NA	NA	NA
1700025M24Rik	0.04306867	0.7089689	4.27958794	0.16566289	0.86842225	NA
1700025N23Rik	5.70582599	-0.3921131	0.42117603	-0.9309957	0.35185581	0.536923
1700025O08Rik	0	NA	NA	NA	NA	NA
1700026D11Rik	1.91893369	0.13477529	1.00796531	0.13371025	0.8936317	NA
1700026F02Rik	0	NA	NA	NA	NA	NA
1700026J12Rik	0	NA	NA	NA	NA	NA
1700026J14Rik	0.12403298	0.70895305	4.27958794	0.16565918	0.86842517	NA
1700027A15Rik	0	NA	NA	NA	NA	NA
1700027F06Rik	0	NA	NA	NA	NA	NA
1700027F09Rik	0.048469	-0.4451501	4.27958794	-0.1040171	0.91715581	NA
1700027H10Rik	0.04534625	0.7089689	4.27958794	0.16566289	0.86842225	NA
1700027I24Rik	0.0519703	0	4.27958794	0	1	NA
1700027J07Rik	1.92623681	0.13936126	1.3942464	0.09995454	0.92038042	NA
1700028B04Rik	0	NA	NA	NA	NA	NA
1700028D13Rik	0	NA	NA	NA	NA	NA
1700028E10Rik	32.8685736	1.05340435	0.25888313	4.06903435	4.72E-05	0.00089853
1700028I16Rik	0.77375756	-1.2025445	1.35286995	-0.8888841	0.3740654	NA
1700028J19Rik	3.25948467	-0.6024458	0.6529165	-0.9226996	0.35616375	NA
1700028K03Rik	8.0895731	0.47452562	0.42781223	1.10919134	0.26734765	0.44817082
1700028M03Rik	0	NA	NA	NA	NA	NA
1700028P14Rik	0.03799125	-0.4451501	4.27958794	-0.1040171	0.91715581	NA
1700028P15Rik	0	NA	NA	NA	NA	NA
1700029B22Rik	0	NA	NA	NA	NA	NA
1700029F12Rik	0.0502434	0	4.27958794	0	1	NA
1700029H14Rik	0.69093043	-1.0499288	1.21259553	-0.8658525	0.38657107	NA
1700029I15Rik	20.9832045	0.82181002	0.29762946	2.76118505	0.0057592	0.03019054
1700029J03Rik	0.05930715	0	4.27958794	0	1	NA
1700029J07Rik	16.7300079	0.098875	0.33475388	0.29536624	0.76771412	0.86393751
1700029M03Rik	0	NA	NA	NA	NA	NA
1700029M20Rik	0.21084405	0.65509585	2.59813085	0.25214121	0.80093192	NA
1700029N11Rik	0.04410202	-0.4451501	4.27958794	-0.1040171	0.91715581	NA
1700029P11Rik	0	NA	NA	NA	NA	NA
1700030A11Rik	1.33685575	0.95293567	1.05289579	0.90506171	0.36543269	NA
1700030C10Rik	9.09408431	0.28200526	0.53563796	0.52648484	0.59855138	0.74633578
1700030C12Rik	0.2430458	1.75948573	3.0796721	0.57132242	0.5677811	NA
1700030C14Rik	0.25215723	-1.6908422	3.09137918	-0.546954	0.58441035	NA
1700030F04Rik	0	NA	NA	NA	NA	NA
1700030I03Rik	0	NA	NA	NA	NA	NA
1700030J22Rik	9.9514301	0.52852575	0.33522744	1.57661838	0.11488336	0.25318131
1700030K09Rik	64.6391607	0.20180226	0.14968805	1.34815212	0.17760946	0.34184086
1700030L20Rik	0.04620439	-0.4451501	4.27958794	-0.1040171	0.91715581	NA
1700030L22Rik	17.3686374	-0.6829874	0.37451883	-1.8236397	0.06820656	0.17762449
1700030M09Rik	0.16365467	0	4.17456995	0	1	NA
1700030N03Rik	0.08533204	0.13190146	4.27958794	0.03082106	0.97541224	NA
1700030O20Rik	0	NA	NA	NA	NA	NA
1700031A10Rik	0	NA	NA	NA	NA	NA
1700031F05Rik	0	NA	NA	NA	NA	NA
1700031L13Rik	0	NA	NA	NA	NA	NA
1700031M16Rik	0.09797512	1.2860205	4.27958794	0.30050101	0.76379503	NA
1700031P21Rik	0	NA	NA	NA	NA	NA
1700034B16Rik	0	NA	NA	NA	NA	NA
1700034E13Rik	0	NA	NA	NA	NA	NA
1700034G24Rik	0	NA	NA	NA	NA	NA
1700034H15Rik	7.33802351	-0.4714478	0.40337679	-1.1687529	0.24250318	0.42058554
1700034I23Rik	12.4019985	0.74398164	0.34647037	2.14731675	0.03176807	0.10328109
1700034J05Rik	23.2772518	-0.1478502	0.3180964	-0.464797	0.64207687	0.77894994
1700034K08Rik	0	NA	NA	NA	NA	NA
1700034P13Rik	9.00408263	0.60720971	0.41552952	1.46129141	0.14393549	0.2956929
1700036A12Rik	0.0536433	0	4.27958794	0	1	NA
1700036G14Rik	0	NA	NA	NA	NA	NA
1700037C18Rik	17.2462795	0.11106888	0.264078	0.4205912	0.67405362	0.80116535
1700037F03Rik	1.74518348	-1.4576634	0.94059626	-1.5497228	0.12120807	NA
1700037H04Rik	76.2091414	-0.2477296	0.15393046	-1.6093608	0.10753748	0.24241377
1700039E22Rik	0	NA	NA	NA	NA	NA
1700039I01Rik	0.14741619	0.70897947	4.27958794	0.16566536	0.86842031	NA
1700039M10Rik	0.22124003	0.12013281	3.17398714	0.03784918	0.96980793	NA
1700040D17Rik	1.0088305	-1.6151412	1.25698521	-1.2849325	0.19881586	NA
1700040F15Rik	0	NA	NA	NA	NA	NA
1700040G22Rik	0	NA	NA	NA	NA	NA
1700040K01Rik	0	NA	NA	NA	NA	NA
1700040P09Rik	0.04534625	0.7089689	4.27958794	0.16566289	0.86842225	NA
1700041A01Rik	0	NA	NA	NA	NA	NA
1700041B01Rik	0.86086101	-0.9085631	1.2786043	-0.7105898	0.47733849	NA
1700041C23Rik	0	NA	NA	NA	NA	NA
1700041G16Rik	1.52261644	0.52981635	0.81875115	0.64710303	0.51756526	NA
1700041I07Rik	0.1366788	-0.458182	4.27864461	-0.1070858	0.91472092	NA
1700041M19Rik	0	NA	NA	NA	NA	NA
1700042G07Rik	0	NA	NA	NA	NA	NA
1700042G15Rik	0.048469	-0.4451501	4.27958794	-0.1040171	0.91715581	NA
1700042O10Rik	17.2304467	1.47606687	0.50176409	2.94175473	0.00326358	0.01994035
1700044C05Rik	0	NA	NA	NA	NA	NA
1700044K03Rik	0	NA	NA	NA	NA	NA
1700045H11Rik	19.8604606	0.99009468	0.60606036	1.63365688	0.10233092	0.23485861
1700046C09Rik	0.04534625	0.7089689	4.27958794	0.16566289	0.86842225	NA
1700047A11Rik	8.0697334	-0.0998505	0.42084368	-0.2372627	0.812453	0.89210002
1700047E10Rik	0.12587164	0.70895305	4.27958794	0.16565918	0.86842517	NA
1700047F07Rik	0	NA	NA	NA	NA	NA
1700047G03Rik	0.16224124	0.70897897	4.1836291	0.16946506	0.86543085	NA
1700047L14Rik	0.0519703	0	4.27958794	0	1	NA
1700047M11Rik	7.28899291	-0.1618714	0.45589395	-0.3550638	0.72254182	0.8339811
1700048B10Rik	0	NA	NA	NA	NA	NA
1700048F04Rik	0	NA	NA	NA	NA	NA
1700048M11Rik	5.94179285	-0.1686654	0.4752547	-0.3548948	0.72266839	0.83407907
1700048O14Rik	0	NA	NA	NA	NA	NA
1700048O20Rik	9.57058175	-1.0020275	0.42285957	-2.369646	0.01780512	0.0678426
1700049E15Rik	1.69444411	-2.3379136	1.06893194	-2.1871492	0.02873164	NA
1700049E17Rik1	0	NA	NA	NA	NA	NA
1700049E17Rik2	0	NA	NA	NA	NA	NA
1700049E22Rik	0	NA	NA	NA	NA	NA
1700049J03Rik	126.186212	0.18735763	0.16243344	1.15344248	0.24872881	0.4284115
1700049L16Rik	0	NA	NA	NA	NA	NA
1700051A21Rik	0.2078407	-0.8537272	3.1798448	-0.2684808	0.78832926	NA
1700052H01Rik	0	NA	NA	NA	NA	NA
1700052I22Rik	0.04775656	0.7089689	4.27958794	0.16566289	0.86842225	NA
1700052K11Rik	12.1076762	0.38939304	0.32779373	1.18792094	0.23486456	0.41187259
1700054A03Rik	0.08969902	0.13190146	4.27958794	0.03082106	0.97541224	NA
1700054K19Rik	0.0519703	0	4.27958794	0	1	NA
1700054M17Rik	2.47890989	0.10105052	0.84184192	0.12003503	0.9044554	NA
1700054O19Rik	2.57312052	1.14456803	0.7271722	1.57399859	0.11548777	NA
1700055C04Rik	0	NA	NA	NA	NA	NA
1700055D16Rik	7.09311856	0.0586095	0.42164187	0.13900303	0.88944776	0.93956344
1700055D18Rik	19.4011339	0.6795646	0.30563548	2.22344799	0.02618561	0.08986361
1700055K11Rik	0	NA	NA	NA	NA	NA
1700055L01Rik	0.04306867	0.7089689	4.27958794	0.16566289	0.86842225	NA
1700056E22Rik	10.1992338	0.4642266	0.38501772	1.20572789	0.22792243	0.40369756
1700057G04Rik	0.84396777	-0.4364245	1.52865664	-0.2854955	0.77526458	NA
1700057H15Rik	0	NA	NA	NA	NA	NA
1700057H21Rik	0	NA	NA	NA	NA	NA
1700060C16Rik	0.30751833	-0.3546831	2.46436435	-0.1439248	0.88555986	NA
1700060C20Rik	0.36583885	-0.2773696	1.87162272	-0.1481974	0.88218698	NA
1700060J05Rik	6.01410704	0.36668366	0.4581601	0.80033957	0.42351408	0.60292524
1700061F12Rik	0	NA	NA	NA	NA	NA

Click here to access/download
ICMJE disclosure form
ICMJE_coi_disclosure.docx

Original Western blots

Figure 1

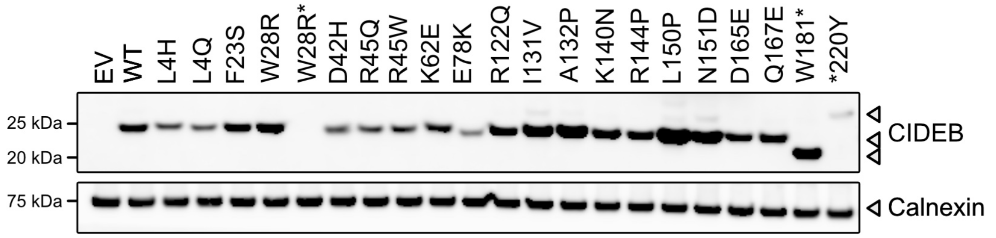

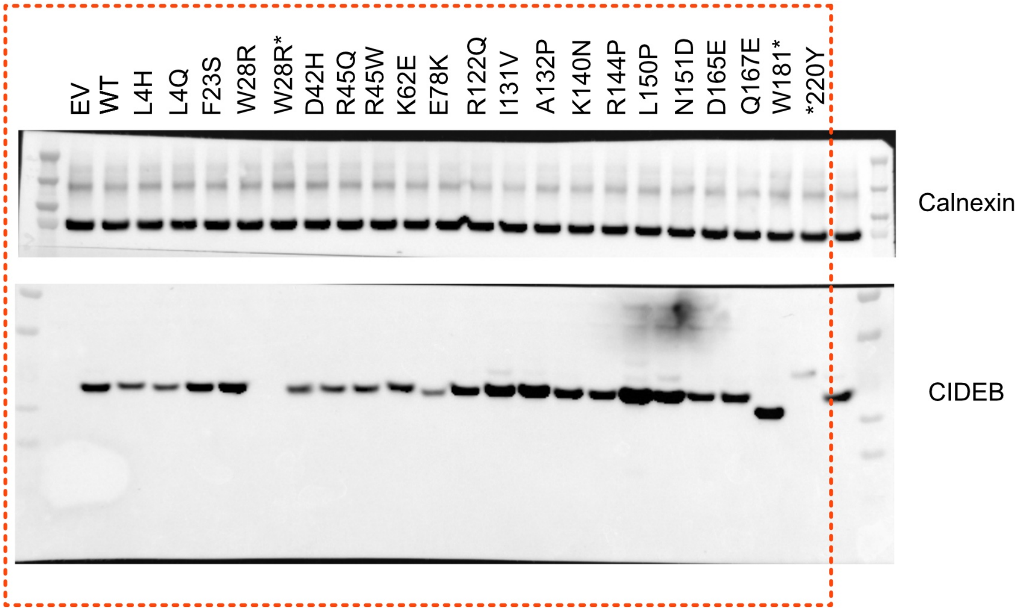

Figure2H

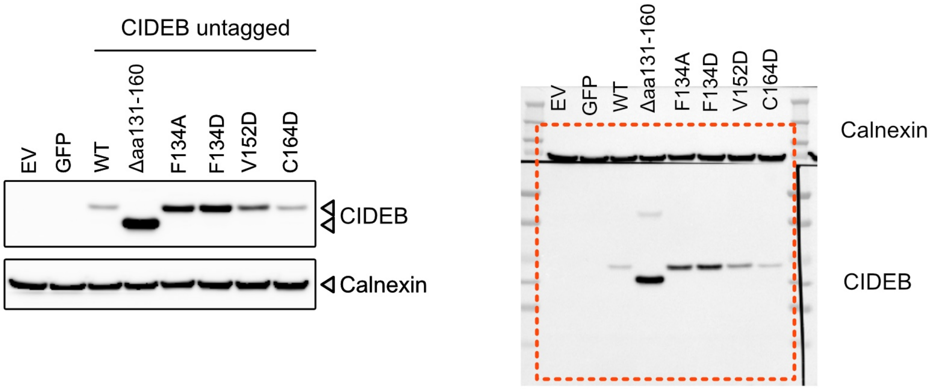

Figure2K (top)

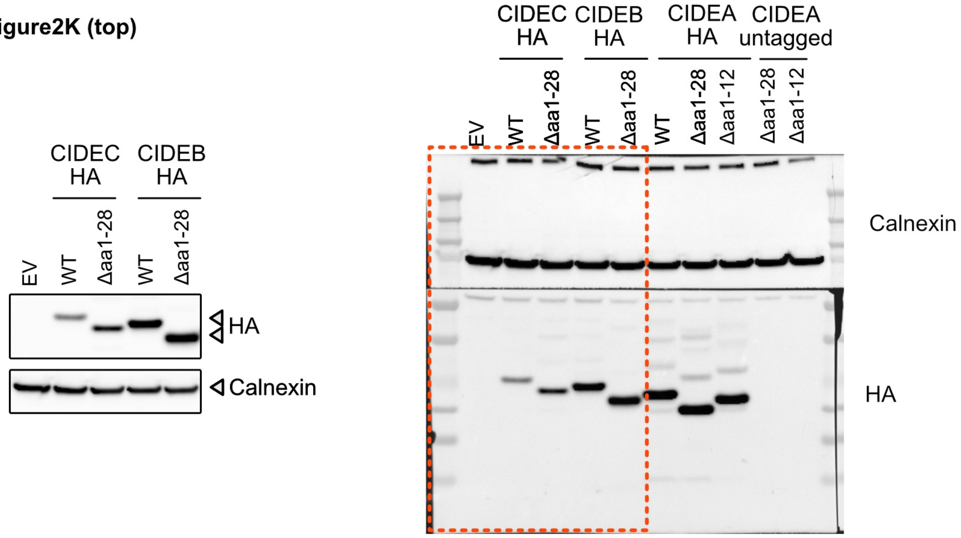

Figure2K (bottom)

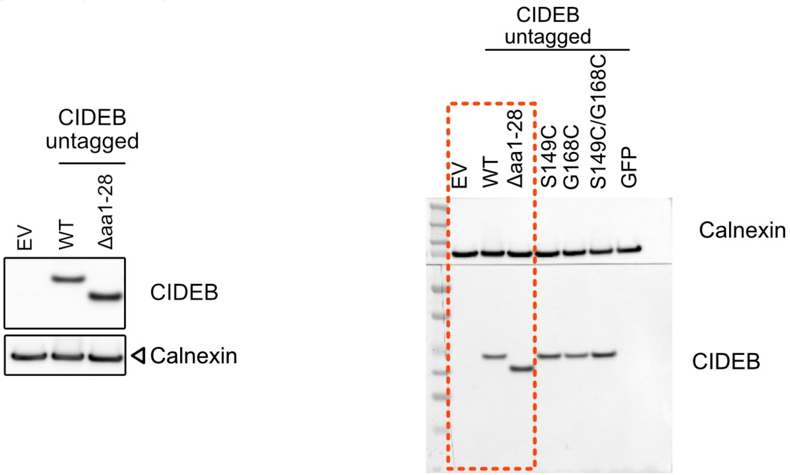

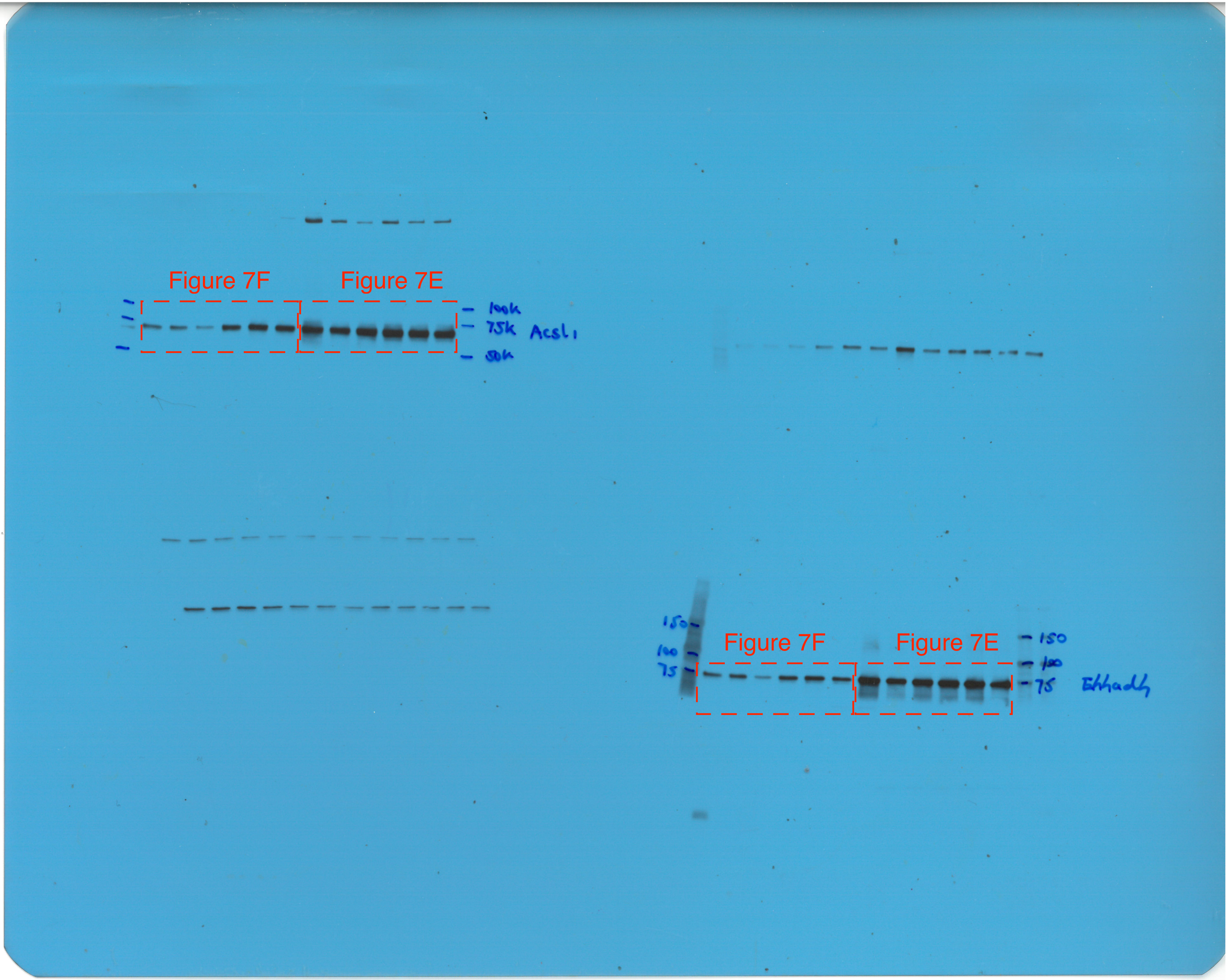
Figure 7F
Figure 7E
100k
75k
50k
Acsl1
150
100
75
Figure 7F
Figure 7E
150
100
75
Ehhadh

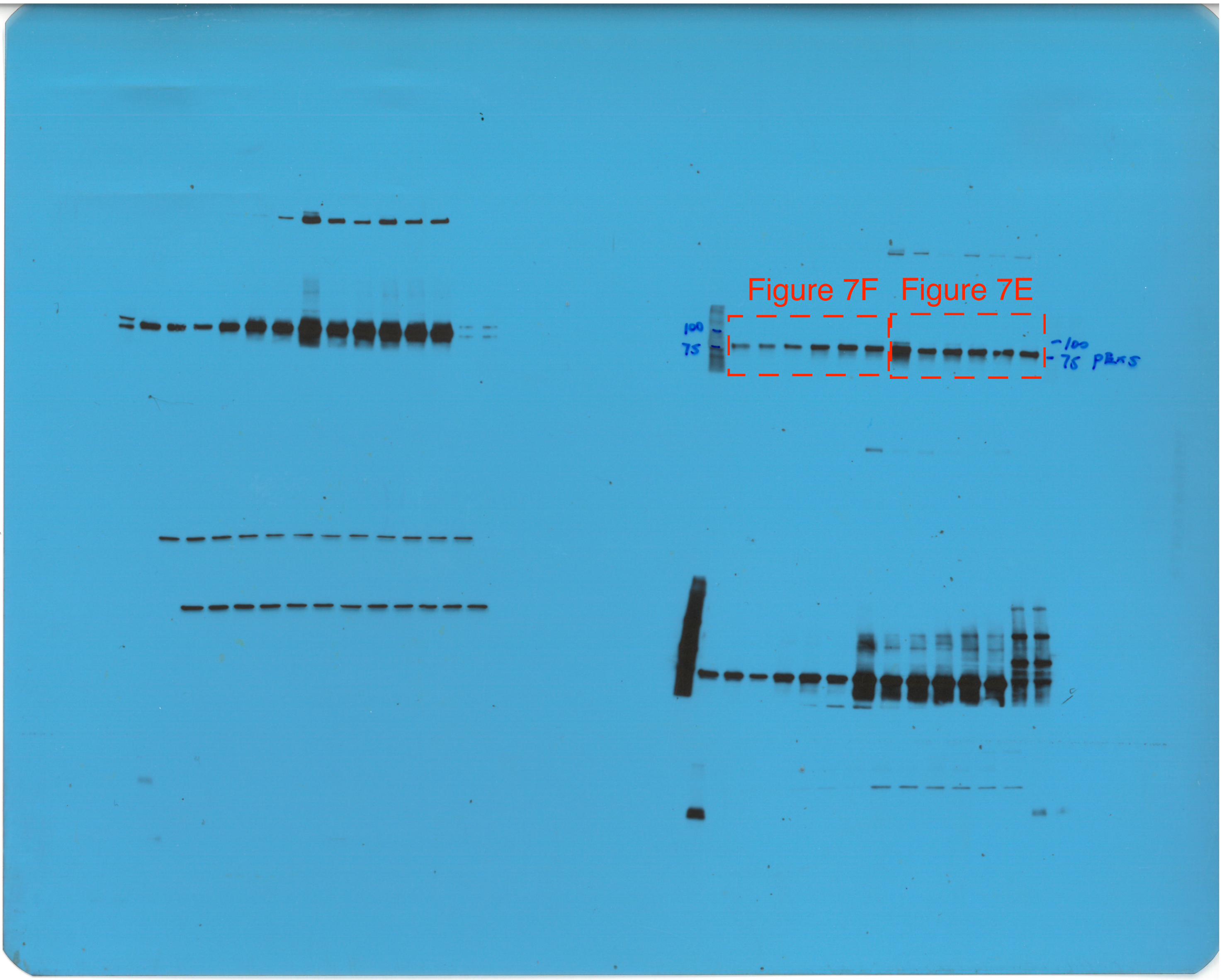
Figure 7F
Figure 7E
100
75
100
75 pERK5

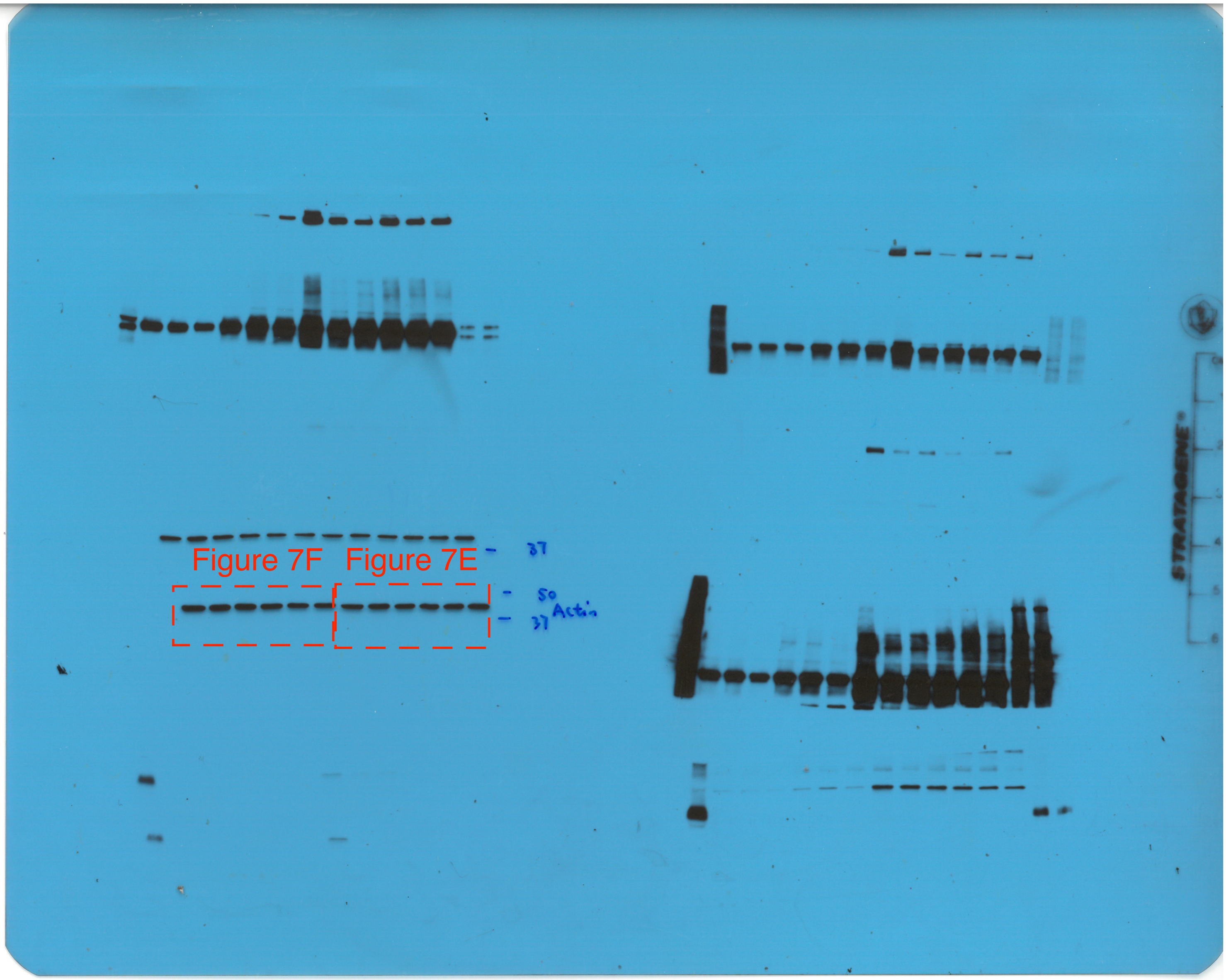
37
Figure 7F
Figure 7E
50
Actin
37

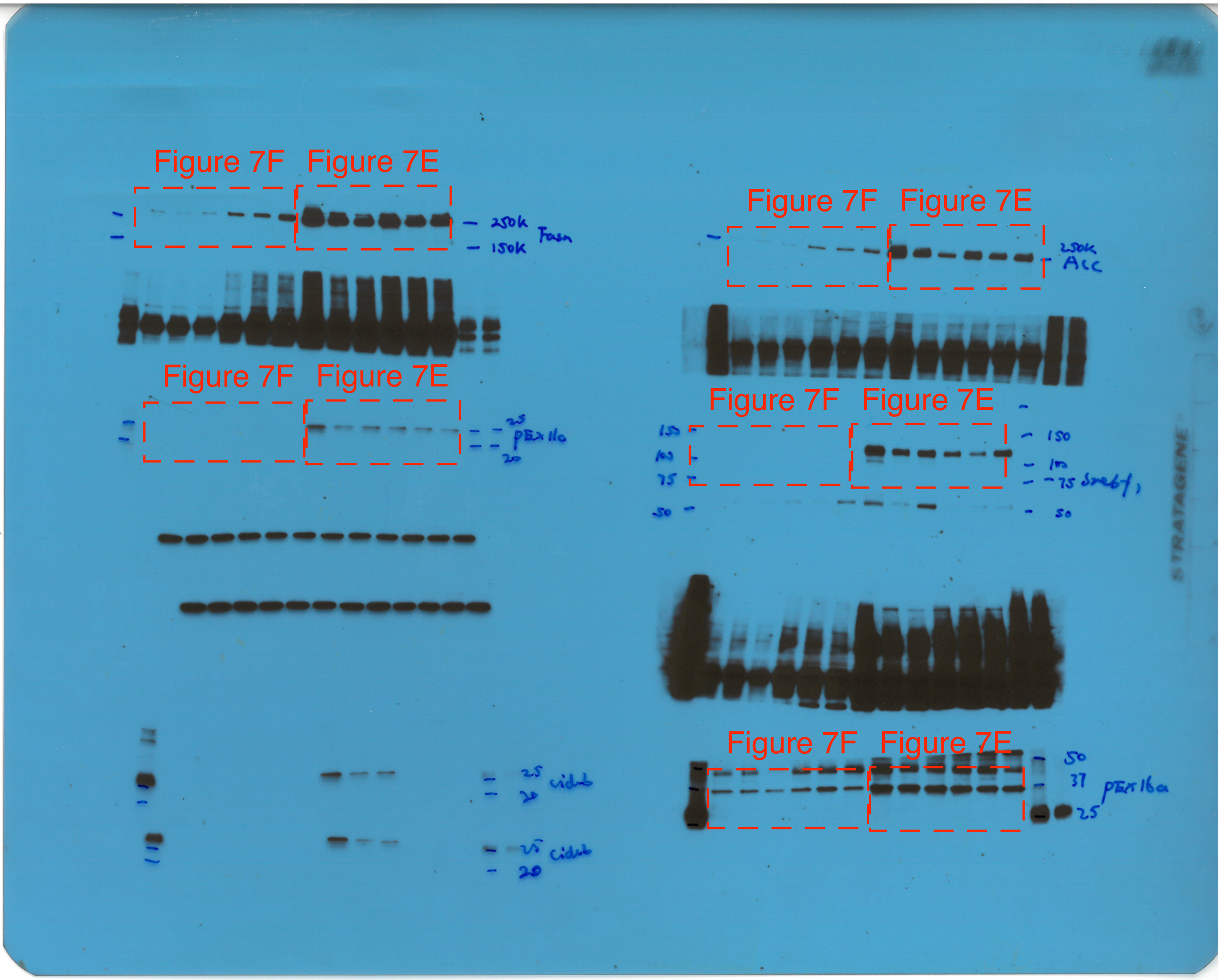
Figure 7F
Figure 7E
250k
150k
Fasn
Figure 7F
Figure 7E
250k
Acc
Figure 7F
Figure 7E
25
20
Figure 7F
Figure 7E
150
100
75
50
Figure 7F
Figure 7E
50
37
25
STRATAGENE

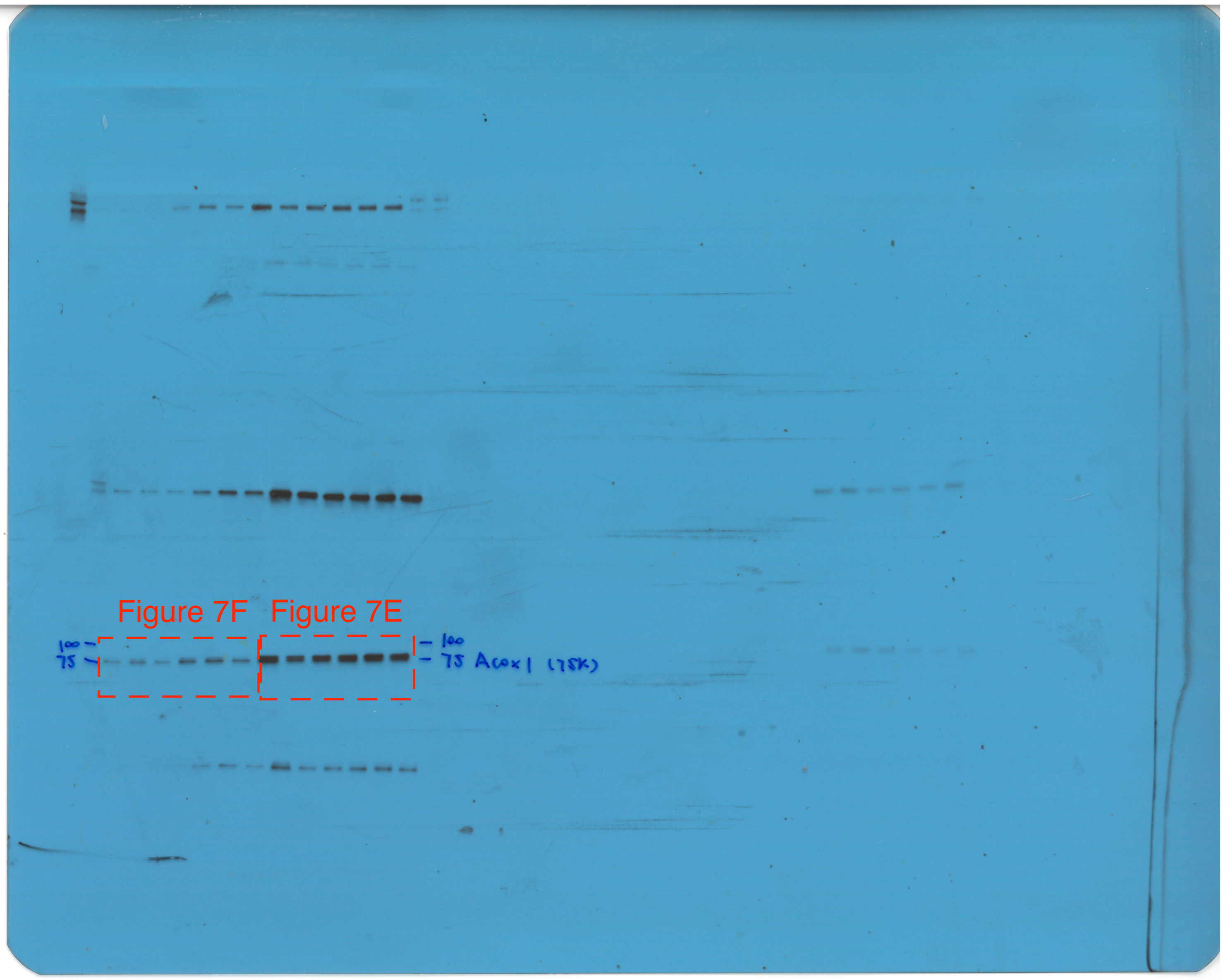
Figure 7F
Figure 7E
100
75
100
75 Acox1 (75K)

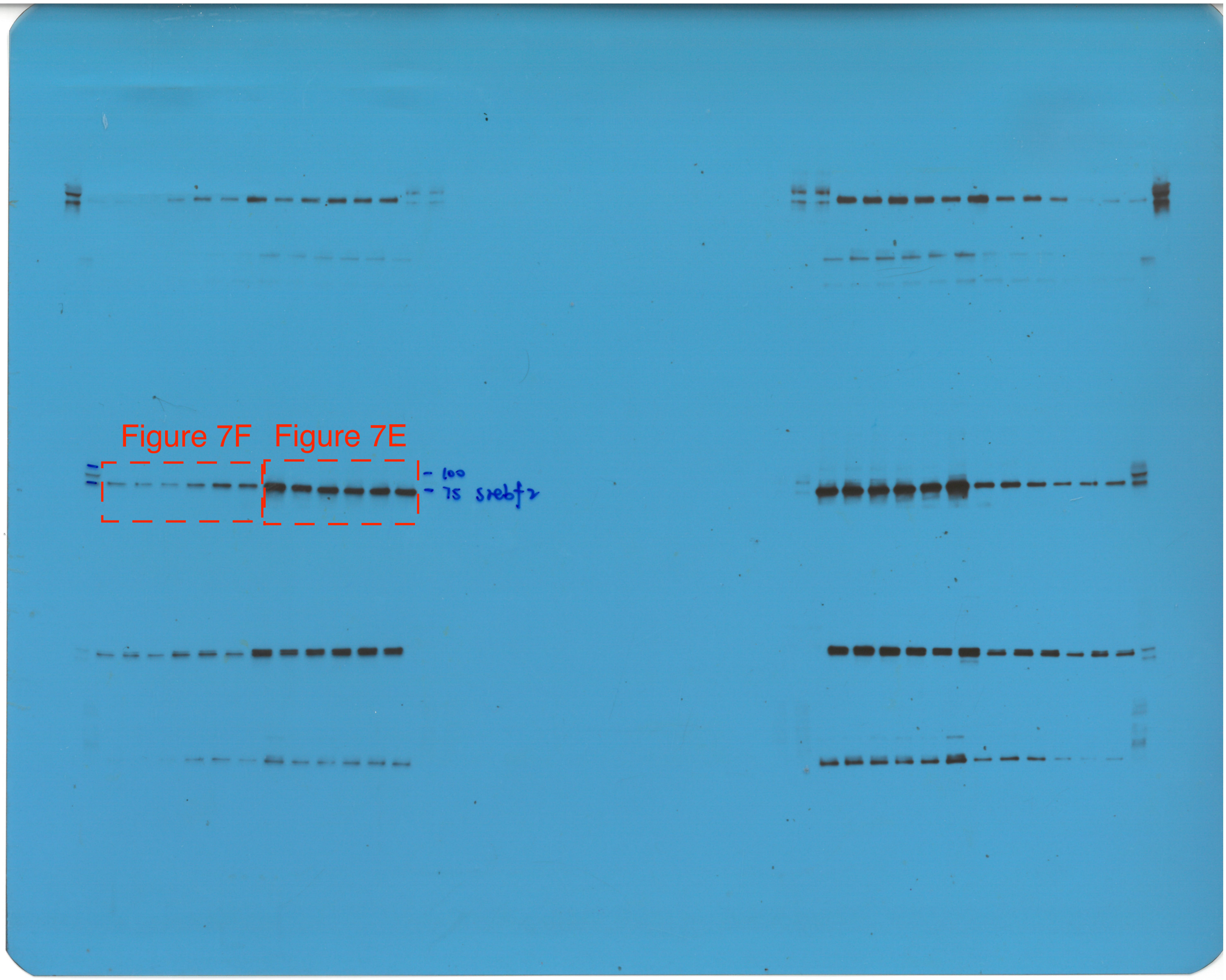

Figure 7F
Figure 7E
100
75
srebf2

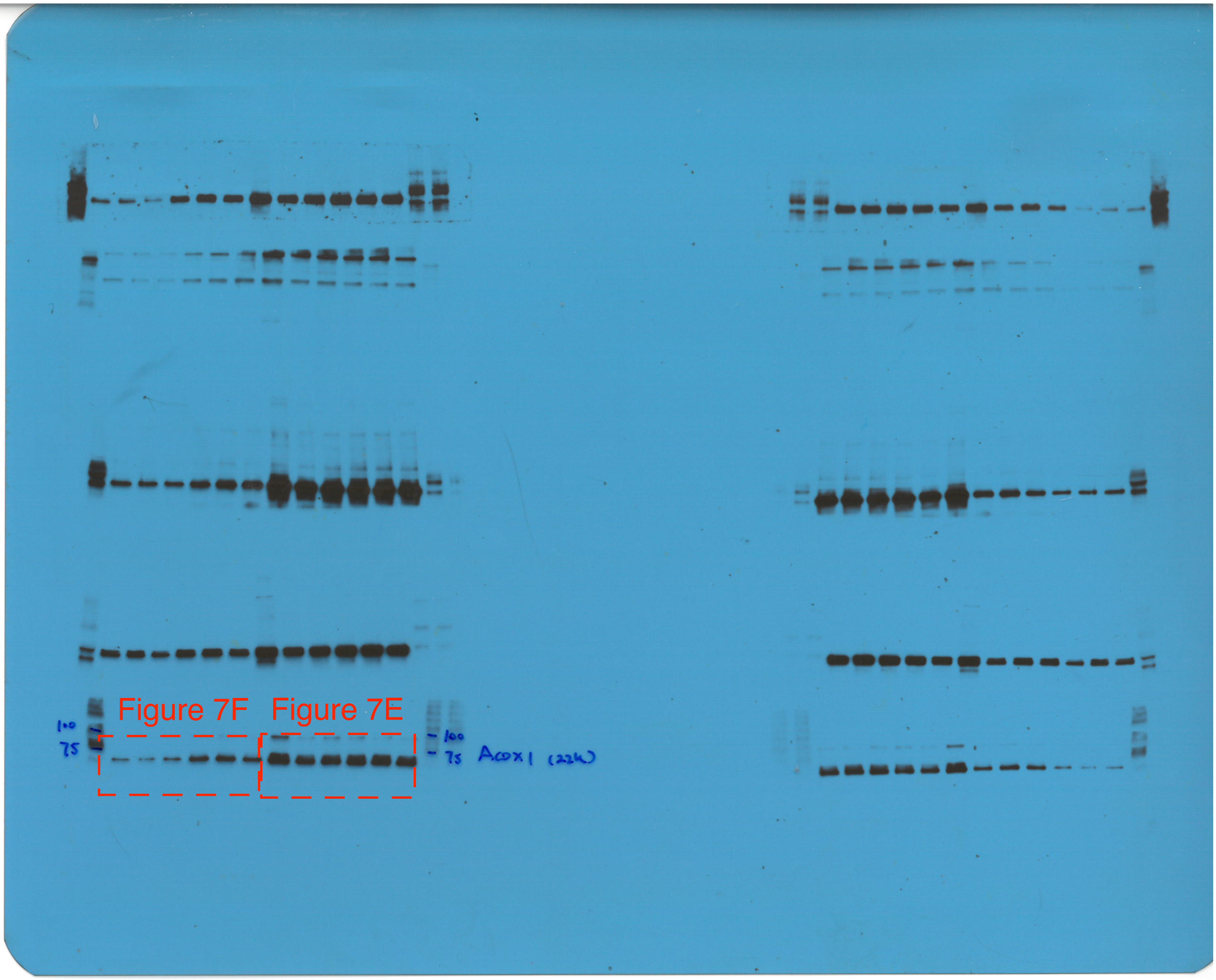

Figure 7F
Figure 7E
100
75
100
75
Acox1 (22k)

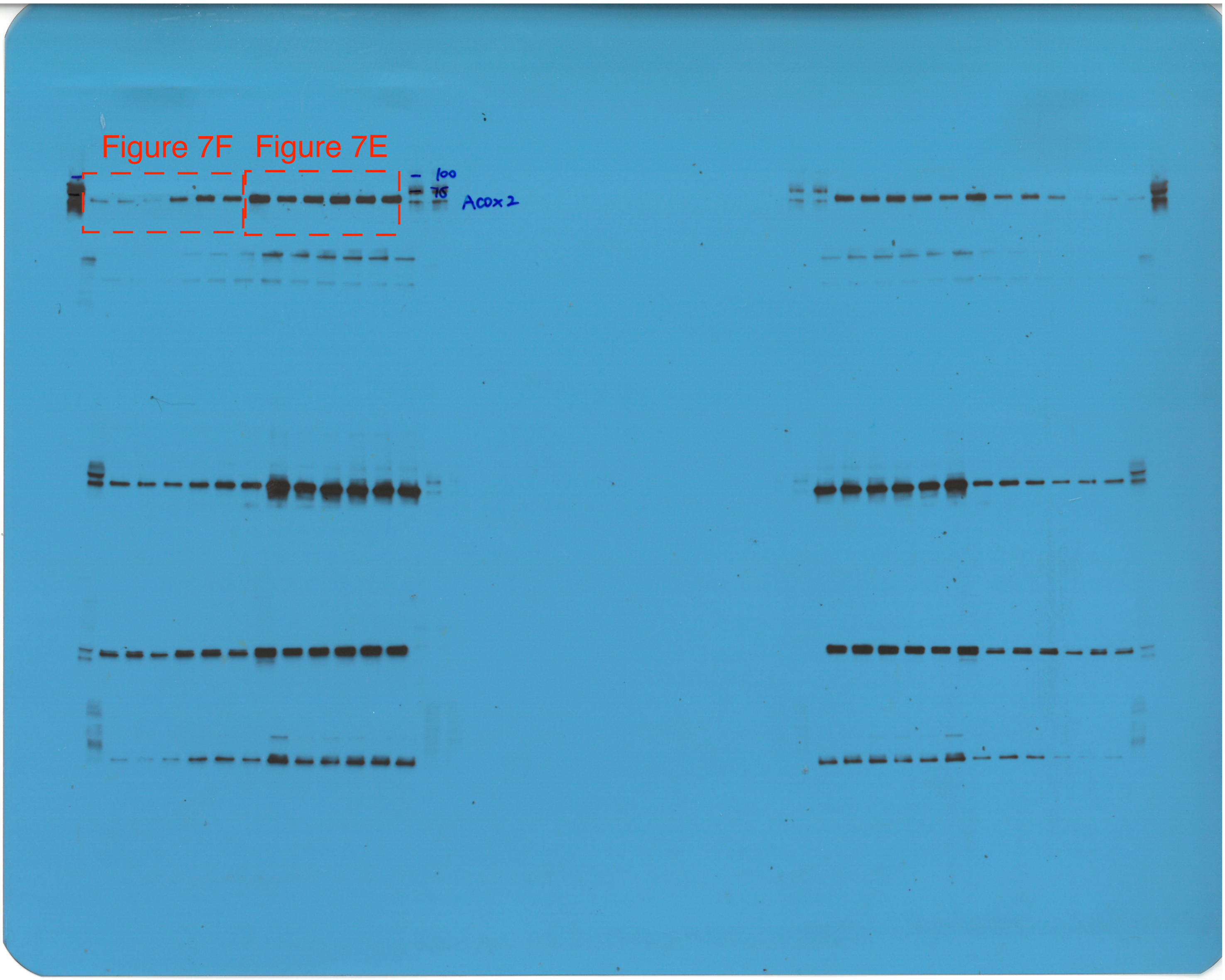

Figure 7F
Figure 7E
100
75
Acox2

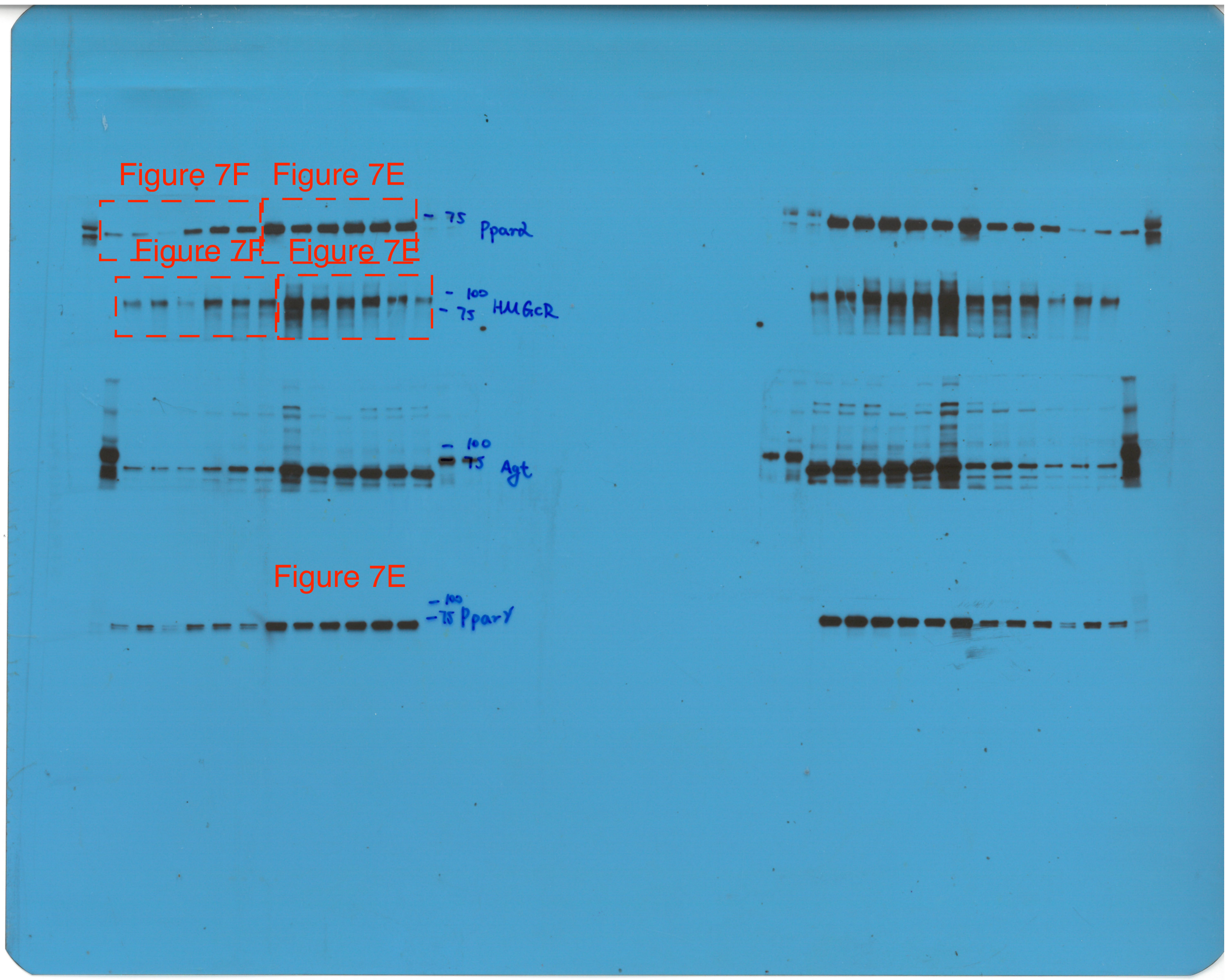
Figure 7F
Figure 7E
75
Pparα
Figure 7F
Figure 7E
100
75
HMGCR
100
75
Agt
Figure 7E
100
75
Pparγ

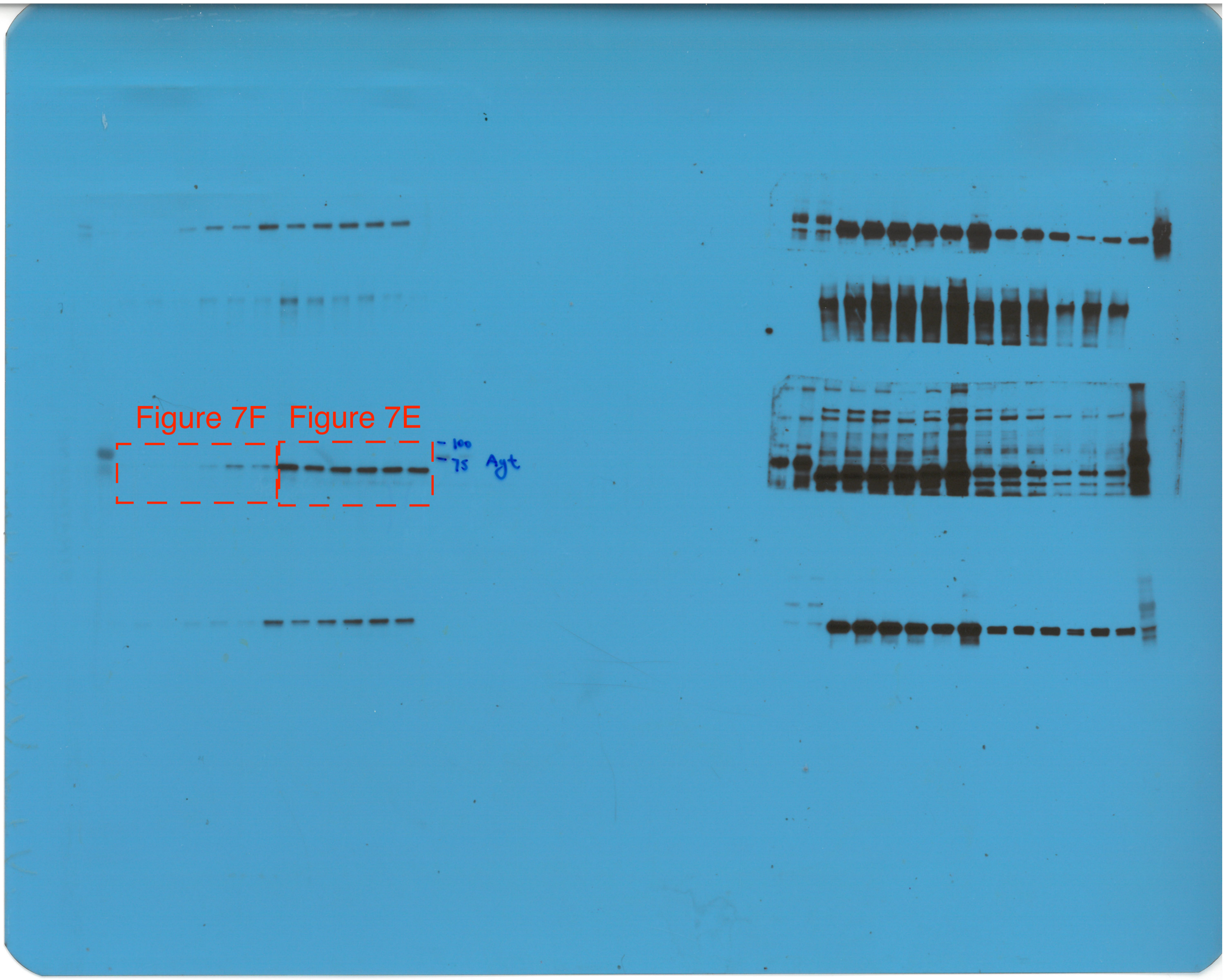
Figure 7F
Figure 7E
100
75
Agt

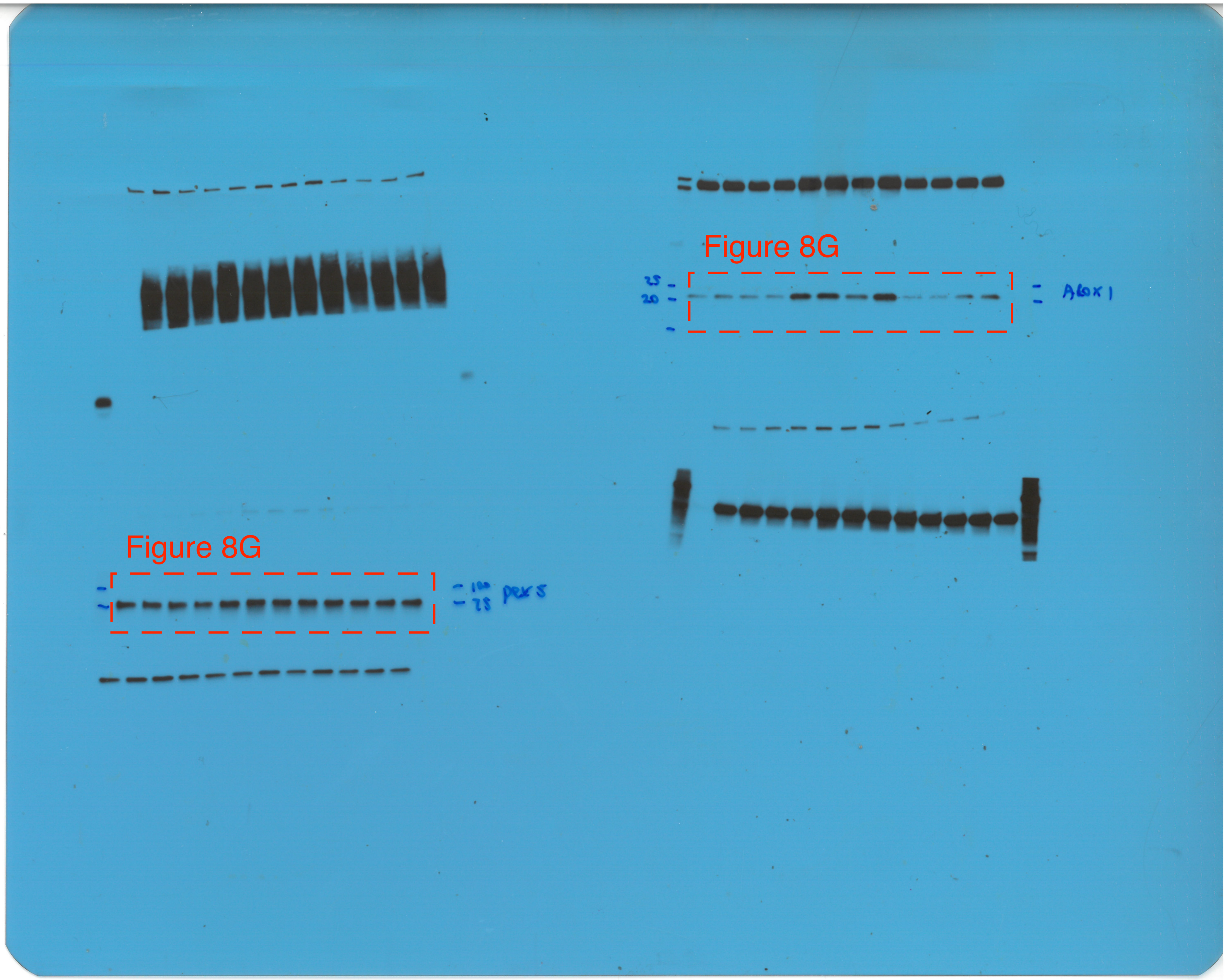
Figure 8G
25
20
AGOX1
Figure 8G
100
75
PEX5

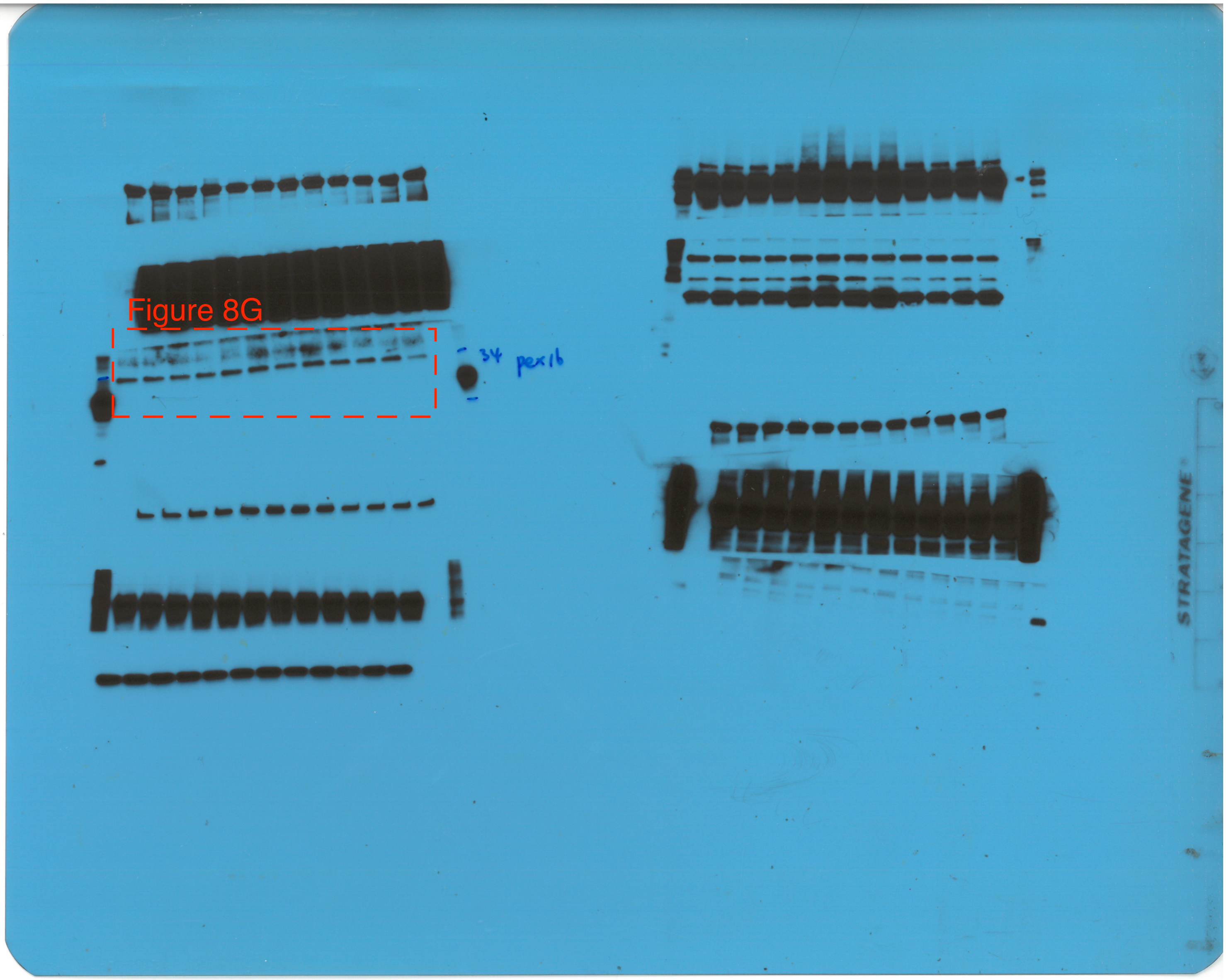
Figure 8G
34
pex16
STRATAGENE

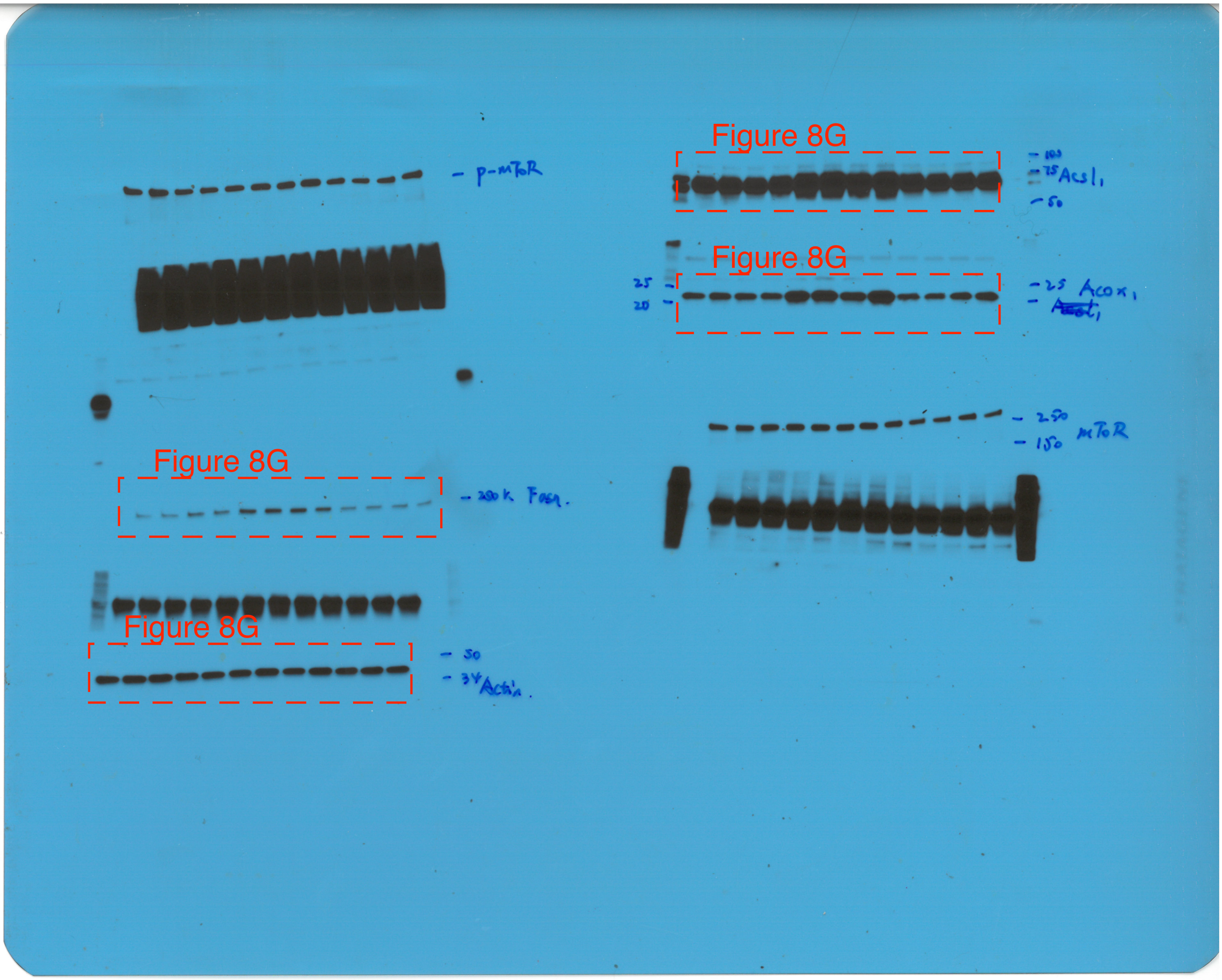
Figure 8G
Figure 8G
Figure 8G
Figure 8G
p-mToR
mToR
Acox1

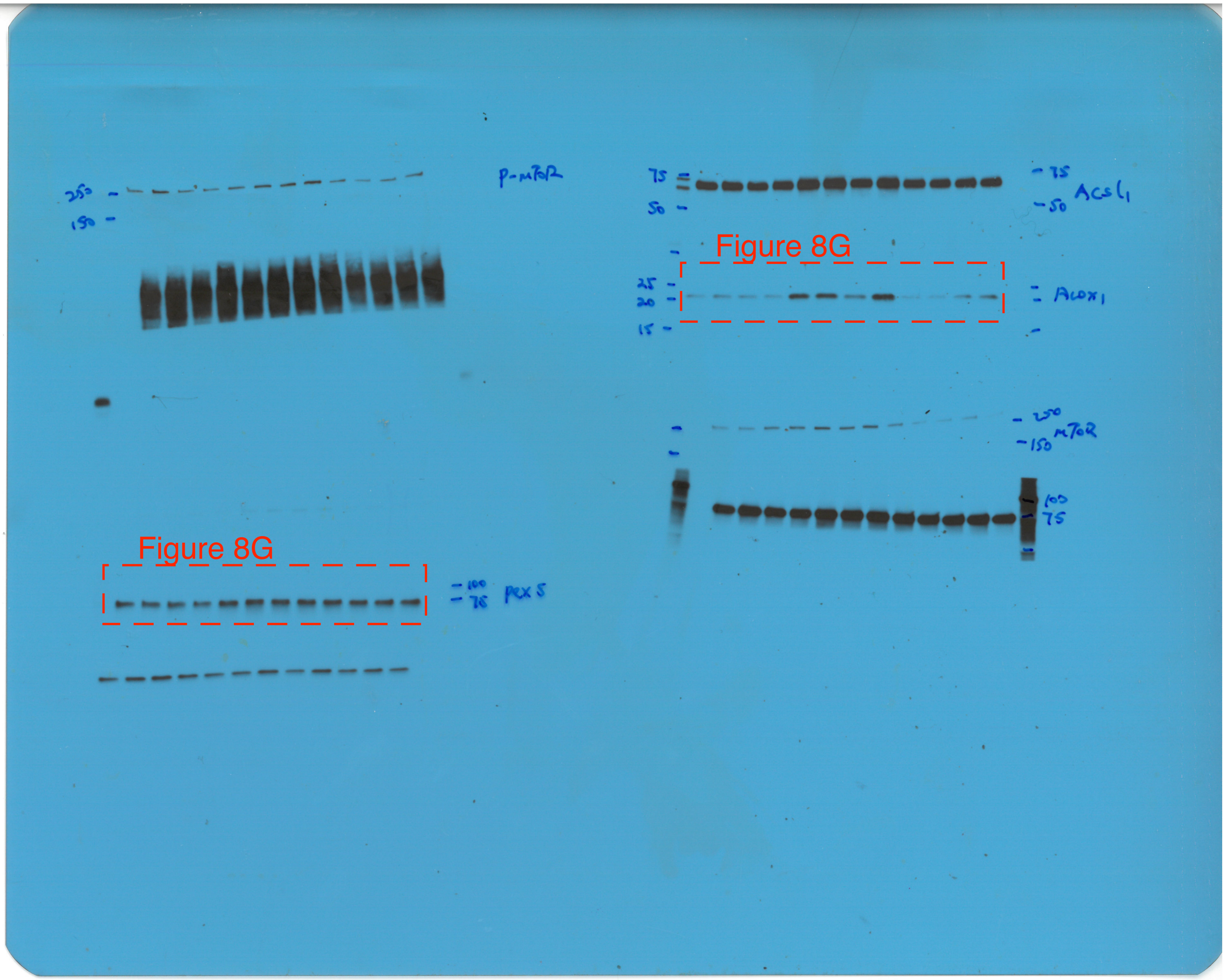

p-mTOR
250
150
75
50
ACSL1
Figure 8G
25
20
15
ALOX1
250
150
mTOR
100
75
Figure 8G
100
75
PEX5

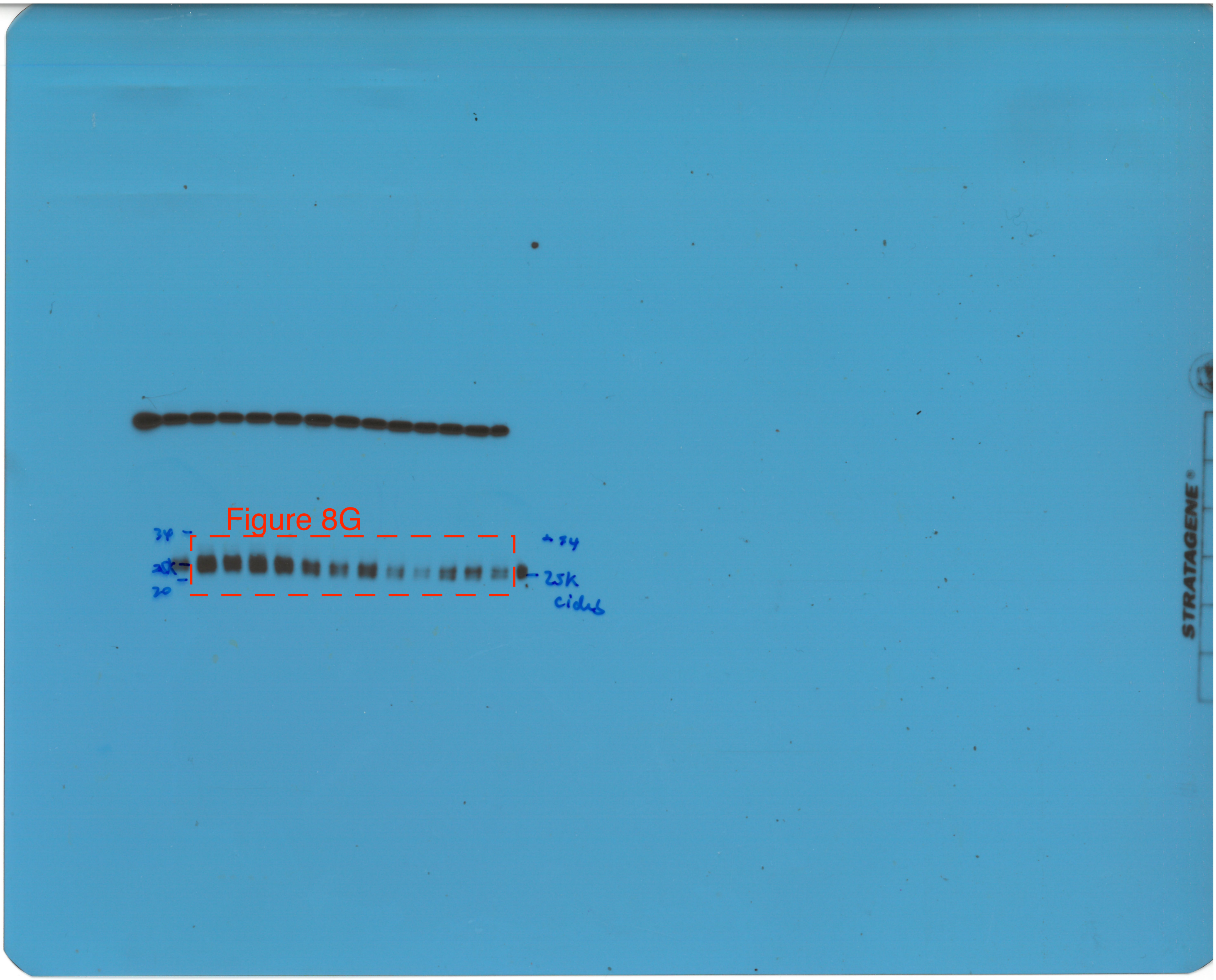
Figure 8G
34
25K
20
34
25K
cidub
STRATAGENE

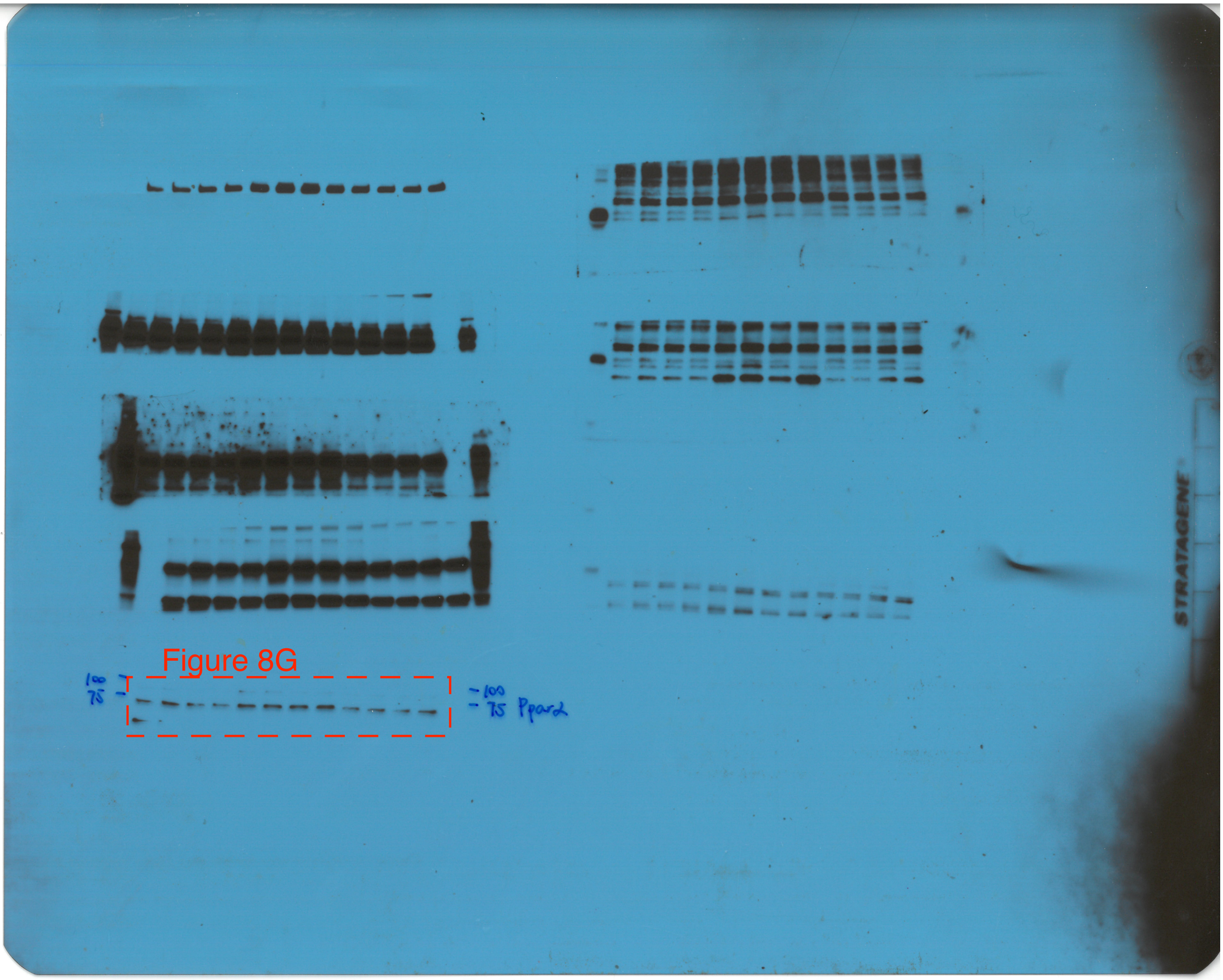
Figure 8G
100
75
-100
-75 Pparδ
STRATAGENE

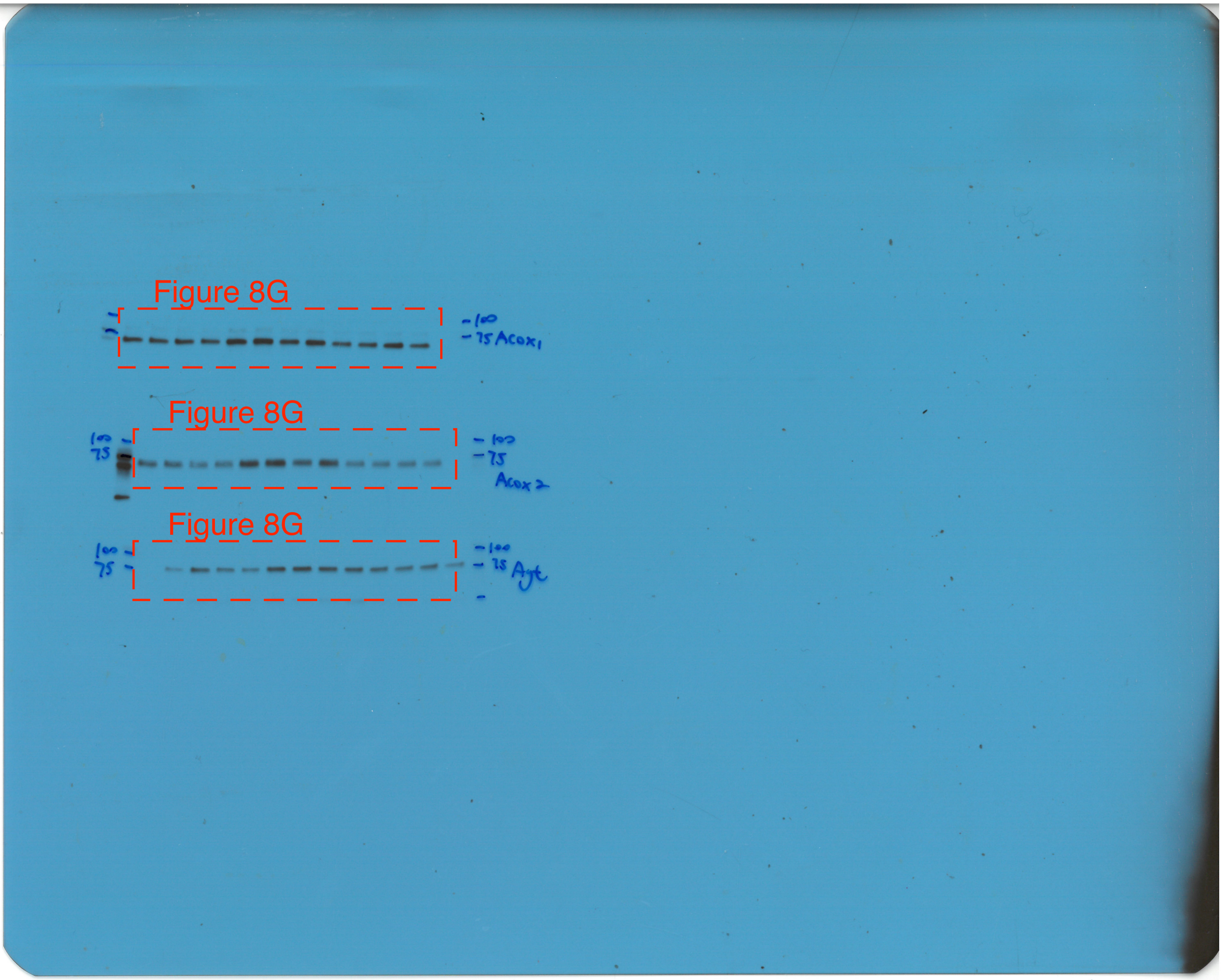
Figure 8G
100
75 Acox1
Figure 8G
100
75
100
75
Acox2
Figure 8G
100
75
100
75 Agt

Figure S2C

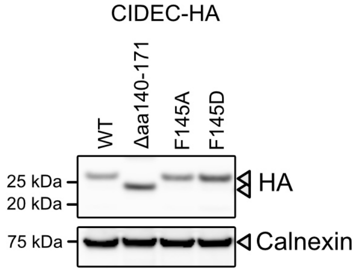

CIDEC-HA
WT
Δaa140-171
F145A
F145D
25 kDa
20 kDa
HA
75 kDa
Calnexin

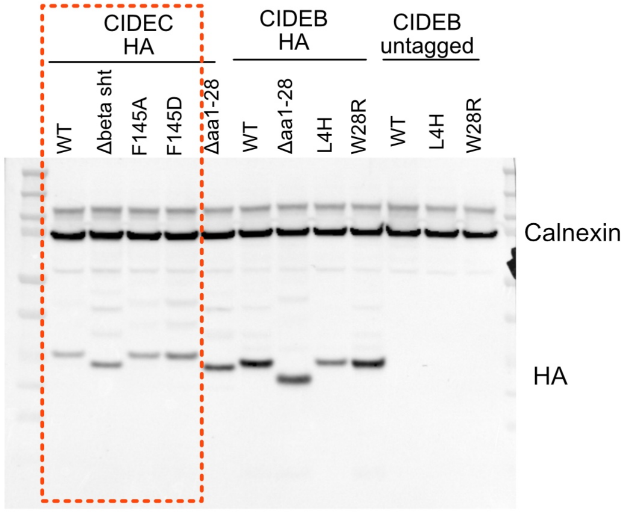

CIDEC
HA
CIDEB
HA
CIDEB
untagged
WT
Δbeta sht
F145A
F145D
Δaa1-28
WT
Δaa1-28
L4H
W28R
WT
L4H
W28R
Calnexin
HA

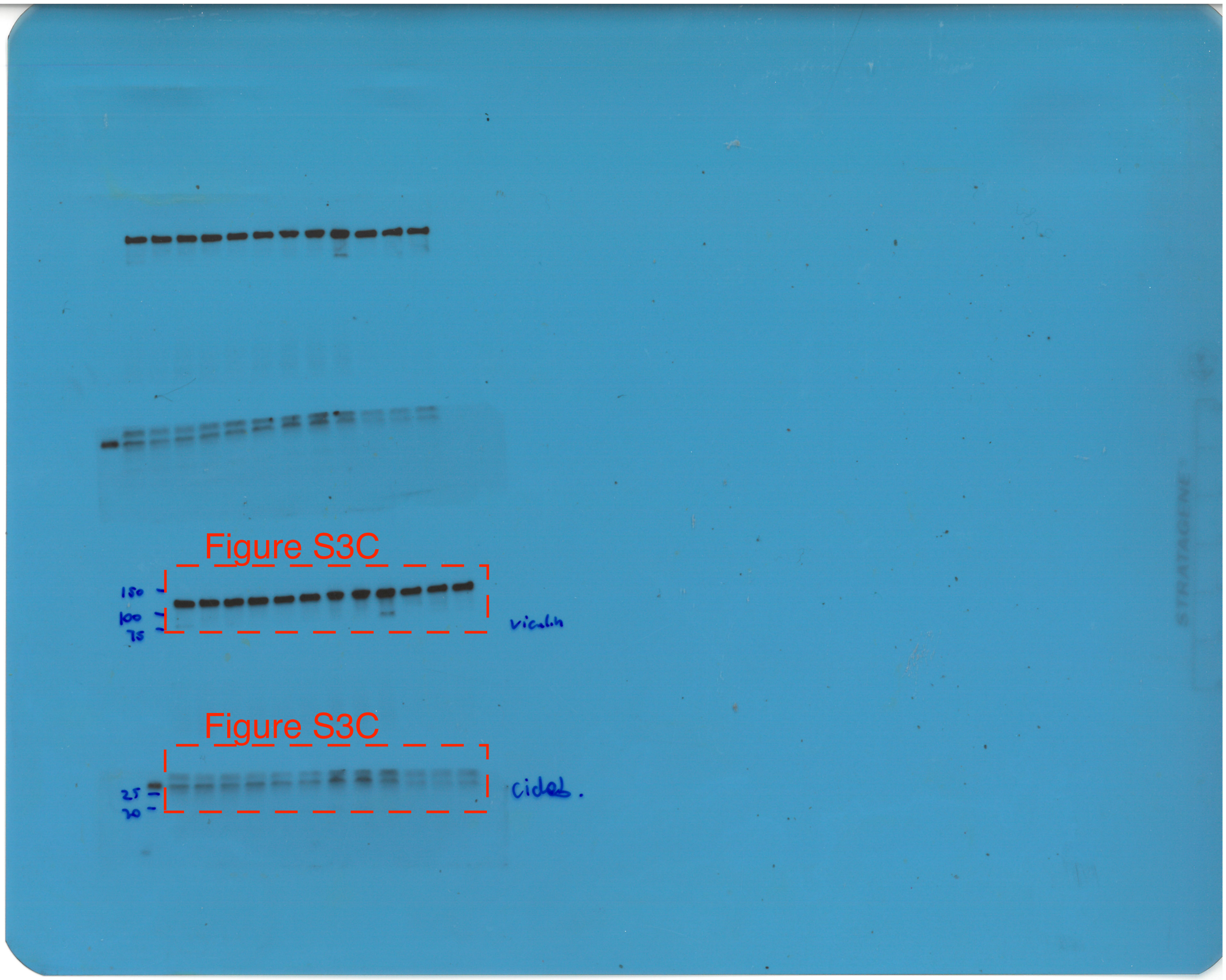

Figure S3C
150
100
75
vinculin
Figure S3C
25
20
cideb.

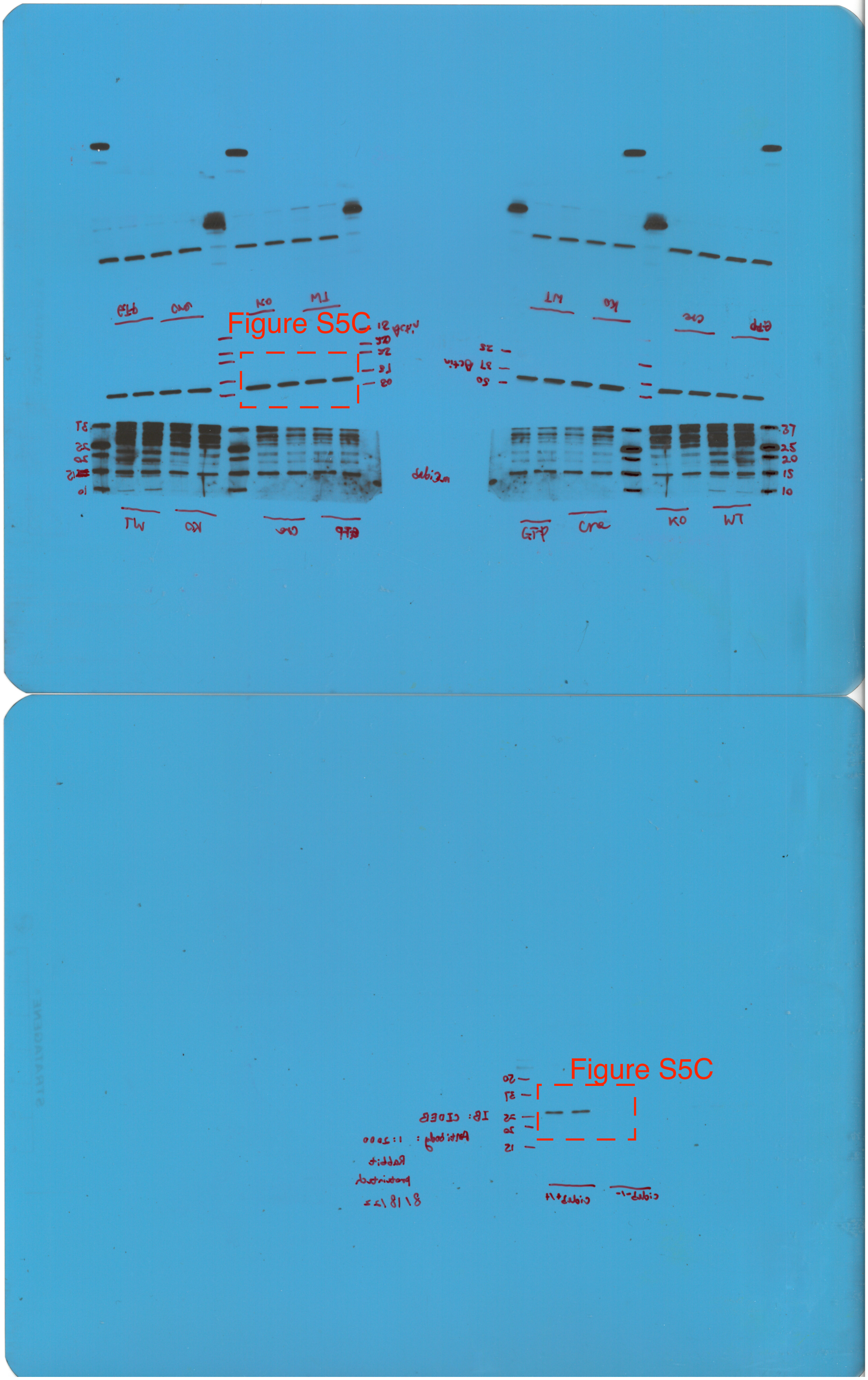

Figure S5C
GFP
Cre
KO
WT
37
25
20
15
10
Figure S5C

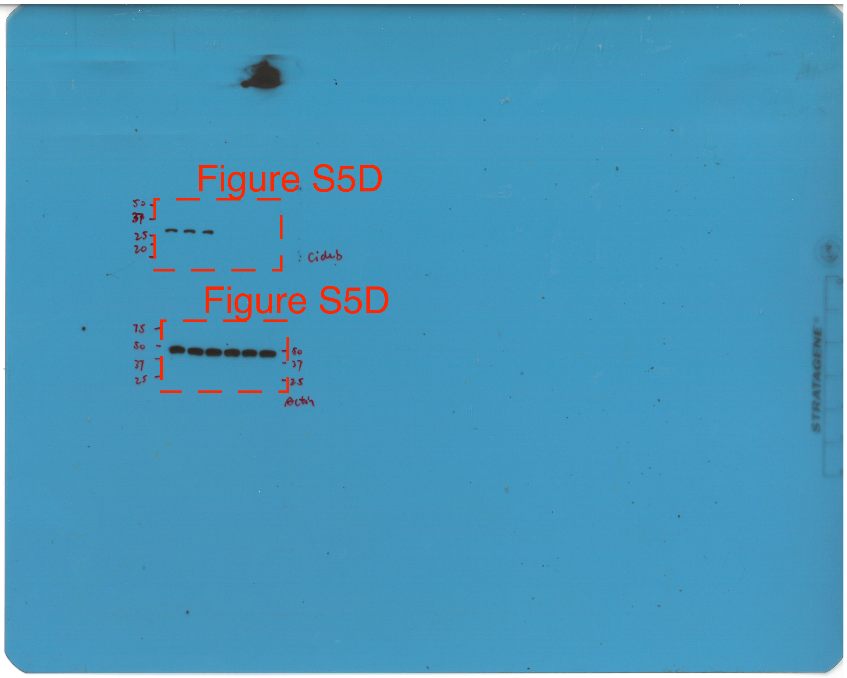
Figure S5D
50
37
25
20
Cideb
Figure S5D
75
50
37
25
50
37
25
Actin
STRATAGENE
